# Stochastic Simulations as a Tool for Assessing Signal Fidelity in Gene Expression in Synthetic Promoter Design

**DOI:** 10.3390/biology10080724

**Published:** 2021-07-29

**Authors:** Elena Righetti, Cansu Uluşeker, Ozan Kahramanoğulları

**Affiliations:** 1 Department of Mathematics, University of Trento, 38123 Trento, Italy; elena.righetti-1@studenti.unitn.it; 2 Department of Chemistry, Bioscience and Environmental Engineering, University of Stavanger, 4036 Stavanger, Norway; ulusekercansu@gmail.com

**Keywords:** synthetic biology, *E. coli*, modelling, stochasticity, simulation, noise, two-component systems, PhoB, PhoR

## Abstract

**Simple Summary:**

Synthetic biology is an emerging discipline, offering new perspectives in many industrial fields, from pharma and row-material production to renewable energy. Developing synthetic biology applications is often a lengthy and expensive process with extensive and tedious trial-and-error runs. Computational models can direct the engineering of biological circuits in a computer-aided design setting. By providing a virtual lab environment, *in silico* models of synthetic circuits can contribute to a quantitative understanding of the underlying molecular pathways before a wet-lab implementation. Here, we illustrate this notion from the point of view of signal fidelity and noise relationship. Noise in gene expression can undermine signal fidelity with implications on the well-functioning of the engineered organisms. For our analysis, we use a specific biological circuit that regulates the gene expression in bacterial inorganic phosphate economy. Applications that use this circuit include those in pollutant detection and wastewater treatment. We provide computational models with different levels of molecular detail as virtual labs. We show that inherent fluctuations in the gene expression machinery can be predicted via stochastic simulations to introduce control in the synthetic promoter design process. Our analysis suggests that noise in the system can be alleviated by strong synthetic promoters with slow unbinding rates. Overall, we provide a recipe for the computer-aided design of synthetic promoter libraries with specific signal to noise characteristics.

**Abstract:**

The design and development of synthetic biology applications in a workflow often involve connecting modular components. Whereas computer-aided design tools are picking up in synthetic biology as in other areas of engineering, the methods for verifying the correct functioning of living technologies are still in their infancy. Especially, fine-tuning for the right promoter strength to match the design specifications is often a lengthy and expensive experimental process. In particular, the relationship between signal fidelity and noise in synthetic promoter design can be a key parameter that can affect the healthy functioning of the engineered organism. To this end, based on our previous work on synthetic promoters for the *E. coli* PhoBR two-component system, we make a case for using chemical reaction network models for computational verification of various promoter designs before a lab implementation. We provide an analysis of this system with extensive stochastic simulations at a single-cell level to assess the signal fidelity and noise relationship. We then show how quasi-steady-state analysis via ordinary differential equations can be used to navigate between models with different levels of detail. We compare stochastic simulations with our full and reduced models by using various metrics for assessing noise. Our analysis suggests that strong promoters with low unbinding rates can act as control tools for filtering out intrinsic noise in the PhoBR context. Our results confirm that even simpler models can be used to determine promoters with specific signal to noise characteristics.

## 1. Introduction

The rapidly growing field of synthetic biology, at the crossroads of molecular biology, genetics and quantitative sciences, aims at developing living technologies by re-engineering the makeup of organisms. The joint effort in this field has been giving rise to methodologies and tools for navigating the highly complex “wiring” of biological systems in a systematic manner. As a result, the recent developments move this field from being trial-and-error-driven towards standardisation as in mature fields of engineering. The applications in this field are designed by channelling the quantitative understanding of the molecular processes to a methodological workflow that can be compared to the use of mechanics in civil engineering. In this respect, very much like computer-aided design became an essential element of mature engineering disciplines, synthetic biology calls for computational methods for containing and accelerating the design process, see, e.g., in [1,2,3,4,5].

Synthetic biology applications are commonly designed by rewiring biological circuits to enhance and benefit from their capacity for certain tasks. The aim, in this setting, is to govern the function of the organism in a logical form by introducing a “computing-like behaviour” [6]. Applications are then obtained by modifying the organisms by targeted interventions that introduce the designed modifications. This vision, which goes back to the production of cheese and alcoholic beverages, extends to more contemporary domains with an industrial ambition, including those with an impact on society. Modified organisms are today envisioned to yield products that ordinarily depend on petrochemicals, e.g., fuels, plastic, and cosmetics. Enhanced biological phosphorus removal (EBPR) is another example, whereby microorganisms such as *E. coli* are used to profit from their inherent regulatory mechanisms that efficiently respond to phosphate starvation [7].

Synthetic biology applications are typically based on introducing a genetic sequence that captures the desired phenotypic variability in the expression of the related genes in the engineered microorganism. Gene promoters are essential elements for gene expression regulation at the transcriptional level. Even a small modification of their nucleotide sequence can significantly change the transcription factor affinity for the promoter and the binding time span. It has been extensively proven that mutations in promoters affect gene expression [8]. Therefore, it comes as no surprise that many synthetic applications are based on mutagenesis to create artificial promoter libraries [6,9,10].

Computational models with quantitative representations of molecular mechanisms are instrumental in the design of these applications. With quantitative models, network dynamics can be explored in an otherwise impossible way. In [11], we have presented one such model of the molecular machinery that regulates the *E. coli* phosphate intake in response to varying environmental conditions. This machinery involves a cascade with a two-component system (TCS) that relays the signal on the external inorganic phosphate concentration to the genetic circuit, thereby maintaining a delicate resource economy for the organism. Our model, based on a chemical reaction network representation, explores the biochemical autoregulation machinery that relays the information on extracellular inorganic phosphate ($P_{i}$) concentration to the genetic components. The ordinary differential equation simulations with our model quantify the dynamic response to varying external $P_{i}$ levels of *E. coli* with which it optimises the expression of the proteins that are involved in the $P_{i}$ intake.

As shown in [11], this model is in agreement with experimental data obtained by employing the Pliar synthetic promoter engineered for $P_{i}$-depletion [12]. As it is based on a deterministic approach, the model captures the mean dynamic behaviour of the system. Gene expression is, however, a stochastic process. Stochasticity has significant effects on the precision of gene regulation. This is due to the few intracellular copies of molecules involved in this process, such as DNA, mRNA and regulatory molecules. Intuitively, when there are large molecule numbers, fluctuations have little effect on the system functions. On the other hand, when the copy numbers are few, variations in even a single molecule can drastically affect the entire system.

Stochasticity in gene expression is partly explained by low molecule numbers [13]. Another aspect is the inherently stochastic nature of biochemical reactions, characterised by rapid and frequent collisions between molecules [14]. In the specific case of gene expression, small numbers of mRNA molecules constantly interact with large amounts of water molecules. This “bombardment” causes a random walk of the reactants. Consequently, the propensity of the reactants to come together is constantly modified [14], and the exact position and velocity of each molecule over time remain unknown. Namely, the process is stochastic. The level of stochasticity in the system is commonly referred to as noise [14]. Cell-to-cell variability results from such fluctuations in gene expression. However, we do not know to what extent variability is inherent or due to external factors. Elowitz et al. [15] suggest that the level of total noise can further be divided into two components, *extrinsic* and *intrinsic*, named after the way they originate.

The distinction between intrinsic and extrinsic noise has consequences on the design of systems with a complex interplay between noise and regulatory systems that need to suppress variability. However, why do some systems tend to reduce noise more than others? Stochasticity can have either beneficial effects or harmful consequences on function. In at least some cases, cells may exploit this source of variability by increasing the fitness of cellular populations and generating long-term heterogeneity [15]; in other cases, stochasticity represents a nuisance or even a barrier to robust functioning [16]. In the specific case of synthetic applications, the interventions can be perceived as tools to control potential sources of error, like intrinsic noise, by reducing or suppressing fluctuations. Transcriptional biosensors, such as the above-mentioned TCS machinery in *E. coli*, with *engineered promoter designs* are primary actors in this setting [6].

The sources of noise have been extensively investigated both theoretically and experimentally [8,15,17,18,19,20,21,22,23]. It remains, however, unclear how intrinsic noise depends on promoter architecture when the regulatory context is considered as in the present paper. Therefore, synthetic promoter designs are subject to analysis for their effect on signal and noise relationship as a result of the transduction of a sensory signal. Our contribution moves in this direction, whereby we make a case for using stochastic simulations as a tool for evaluating the signal and noise relationship in synthetic biology applications within a sensory signal transduction context. For this, building on our results in [24], we first analyse the noise in *E. coli* phosphate economy with the quantitative model in [11]. After reviewing the biological background of the TCS system that regulates the $P_{i}$ uptake, we give an overview of the stochastic framework we use and illustrate it with examples. Reducing the number of variables in our analysis, we then obtain a reduced model by quasi-steady-state approximation. Our reduced model is conservative on the steady-state behaviour. We compare the analysis outcomes with both models by resorting to commonly-used metrics and highlight the similarities. We conclude by discussing our results in the broader context.

Our analysis is based on the notion that computational models are virtual labs with easily manageable experimental settings. We use these features to investigate the sources and consequences of intrinsic noise [25] in the PhoBR TCS context. For this, by means of repeated simulations, at the single-cell level, we relate changes in promoter designs to fluctuations in gene expression levels. Ultimately, our goal consists of identifying phenotypes that can optimise $P_{i}$ intake by scanning a collection of synthetic promoters. We argue that our analysis provides insights that can guide the design of synthetic applications, where the effect of stochasticity can be predicted and controlled.

## 2. Phosphate Economy of *E. coli*

Cells rely on accurate control of signalling systems to adapt to environmental changes. Consistent growth requires proper regulation of the adaptive response to hostile environmental conditions. Inorganic phosphate ($P_{i}$) is an essential metabolite, which is, however, normally in short supply in the environment. *E. coli* has developed a specific mechanism to fine-tune the proper response to $P_{i}$ starvation conditions, i.e., to acquire phosphate with high affinity and store it.

The regulatory mechanisms in *E. coli* that control the $P_{i}$ uptake involve the interplay between two complementary mechanisms. When the external $P_{i}$ concentration is above the millimolar range, $P_{i}$ is transported into the cell mainly by the low-affinity $P_{i}$ transporter (Pit) system, which is constitutively expressed and dependent on the proton motive force [26]. However, when the external $P_{i}$ concentration falls below 0.2 mM, the high-affinity Phosphate specific transport (Pst) system is induced. This triggers the expression of an operon in the Pho regulon that also includes an ABC transporter, which actively transports $P_{i}$ by ATP consumption. Both Pit and Pst are highly specific for $P_{i}$.

Phosphate response, intake and, in general, its economy are regulated by the Pho regulon, which, in return, relies on the PhoBR two-component system (TCS). This seemingly simple machinery is the *hub* of the phosphate signal transduction pathway [27] as it relays the input signal to the genetic circuit. The PhoBR TCS consists of an input-sensitive histidine kinase, PhoR, and a cognate response regulator, PhoB. The Pst system thus involves a positive feedback loop, and it induces its own expression via the TCS consisting of PhoR and PhoB, which is the transcription factor (TF) that PhoR activates. More specifically, PhoBR TCS components can be described as follows:

**PhoR** is a homodimeric histidine kinase capable of autokinase, phosphotransfer and phosphatase activities. It includes a membrane-spanning region, a PAS domain, a CA (i.e., catalytic ATP-binding) domain at its C terminus and a DHp (i.e., dimerisation/histidine phosphorylation) domain. Each domain is associated with at least one protein function. The PAS domain is probably involved in signal perception activities. The DHp domain contains all the residues necessary for PhoR phosphatase activity [28]. The CA domain harbours the enzymatic activity for transferring a phosphoryl group from ATP to the DHp domain [29]. Specifically, PhoR autophosphorylates in *cis* in response to depletion of inorganic phosphate ($P_{i}$) and transfers the phosphate unit to PhoB [30]. On the other hand, in response to $P_{i}$-repleted conditions, PhoR acts as a phosphatase by dephosphorylating PhoBp, thus restoring the response regulator to its original inactive state.

**PhoB**, the response regulator of the PhoBR TCS, relays the incoming sensory stimuli to the genetic components. Specifically, PhoB dimerises upon phosphorylation to form a stable dimer that acts as an active transcription factor. It binds cooperatively to a specific DNA sequence, the *Pho box*, and regulates the Pho regulon [11,27,29,31], thereby enabling the TCS component expression and implementing positive autoregulation.

**Figure 1:**
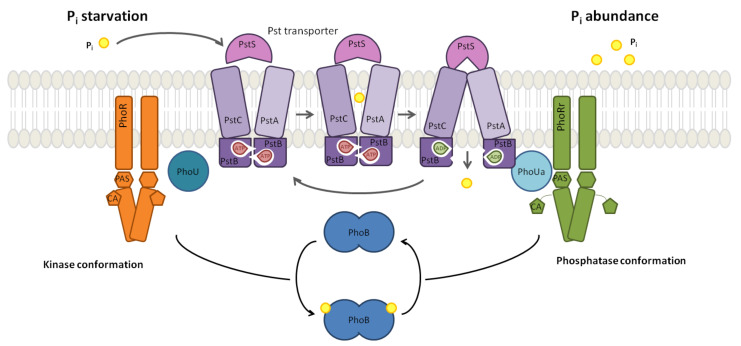
The mechanism of PhoBR TCS response to different environmental conditions. The figure summarises the phosphate response mechanism of the PhoBR TCS. The Pst transporter’s conformational change is based on an ATP-switch model; the outward- and inward-facing conformations signal $P_{i}$ starvation and abundance, respectively. PhoU mediates the interaction between the transporter and PhoR. When external $P_{i}$ is high, PhoU may interact with PhoR through both the PAS and CA domains leading to PhoR phosphatase conformation. Figure adapted from in [29,32].

The mechanistic interplay of PhoB and PhoR, depicted in Figure 1, with their context regulates the adequate response to the sensed environmental $P_{i}$ concentration: $P_{i}$ intake by the Pst system is a negative process, whereby a high external $P_{i}$ concentration turns the system off; the activation is the default state. The current evidence suggests that the TCS mechanism is turned off by the PhoU protein that monitors the ABC transporter activity [29]. In mechanistic terms, when there is sufficient $P_{i}$ flux, PhoU stabilises the PhoR. This mechanism prevents PhoR from relaying the signal to the transcription factor PhoB. By contrast, when the external $P_{i}$ concentration is limited, PhoU does not inhibit the TCS. As a result of the decrease in the external $P_{i}$ concentration, the concentration of PhoR molecules that are not inhibited by PhoU increases. Thus, the autophosphorylation activity of PhoR dimers provides a proxy for the external $P_{i}$ concentration signal as the autophosphorylation of PhoR dimers relays the Pst signal.

The Chemical Reaction Network (CRN) that models this system as in [11] can be found in the Supplementary Materials. The model describes the signal transduction processes downstream of PhoU to the genetic components and the feedback of the gene expression to the Pst system. In this model, the input stimulus corresponding to the external $P_{i}$ concentration is given by a scalar factor for the PhoR autophosphorylation activity: the reactions r01, r02, r03 and r04 model the signal transduction from PhoR, where fc is this factor describing the PhoR activity resulting from the external $P_{i}$ concentration. The fc=1.0 models the full starvation response to the external $P_{i}$ concentration of 0 μM. An increase in the external $P_{i}$ concentration and the resulting inhibition of PhoR by PhoU is modelled by a decrease in the fc. Thus, fc=0 models a $P_{i}$ concentration over 0.2 mM whereby the phosphate intake is handed over to the Pit system.

Apart from the TCS components, our model contains the alkaline phosphatase **PhoA**, which is one of the several Pho regulon proteins involved in phosphorous assimilation [29,33]. PhoA expression is regulated by the phosphorylated PhoB dimer. Because PhoA is not involved in the positive feedback that characterises the PhoBR TCS, PhoA concentration is considered as the yield of the PhoBR system.

Phosphorylated PhoR activates PhoB by phosphotransferase (r05, r06, r07, r08, r09, r10). Phosphorylated PhoB dimerises to constitute an active transcription factor (r11, r12) and binds the promoter region of PhoA and PhoB genes to activate their transcription (r16, r17, r18, r19). The factors bf and uf in reactions r16, r18, r17, and r19 are scaling factors that model the affinity of the active transcription factor to the promoter region. Their default values of 1.0 result in the control model, whereas variations in bf and uf model synthetic promoters that can be stronger or weaker.

The bifunctional role of the histidine kinase PhoR that performs two opposing tasks provides structural robustness for the TCS [34]: on the one hand, PhoR activates the PhoB dimers as described above. On the other hand, it dephosphorylates the phosphorylated PhoB (r13, r14, r15). The activated promoters transcribe the mRNA molecules for the expression of PhoA, PhoB and PhoR (r20, r21, r22, r23, r24), which can be subject to degradation or dilution (r25, r26, r27, r28, r29).

**Figure 2:**
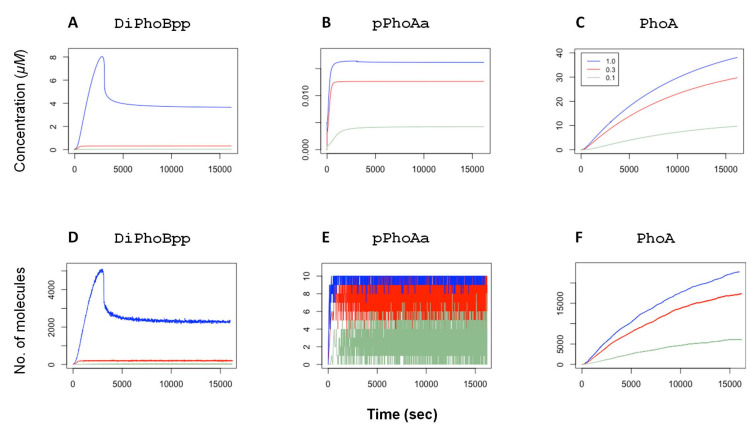
Top. Deterministic time-series plots with ordinary differential equation simulations display the response to the variations in external $P_{i}$ concentrations for (**A**) DiPhoBpp, (**B**) pPhoAa and (**C**) PhoA. The external $P_{i}$ concentration is given with the fold-change fc. A higher external $P_{i}$ concentration is modelled with a smaller factor and vice versa. The different fc values are colour-coded in the legend. Bottom. Stochastic time series plots with different external $P_{i}$ concentrations for (**D**) DiPhoBpp, (**E**) pPhoAa and (**F**) PhoA. The colour codes of the curves are as in the top row. As in the deterministic simulations, a higher external $P_{i}$ concentration is given with a smaller factor fc, colour-coded in the legend. The number of promoters, given by 10 plasmids, gives rise to a greater noise in the number of active promoters pPhoAa in comparison to those in active transcription factor DiPhoBpp and PhoA. PhoA quantifies the yield.

The control model in [11] is parameterised within the biologically feasible range and the parameter values are narrowed down by random restart least-squares optimisation by fitting the model dynamics to experimental data. The deterministic simulation plots in Figure 2 display the concentration dynamics of the active transcription factor dimers DiPhoBpp, the active promoter pPhoAa and the protein PhoA. As described above, the external $P_{i}$ concentration is modelled by the fold change fc applied to the autophosphorylation rate of the reactions r01 and r03 as this rate is a function of the ABC transporter activity. These simulations, as depicted in Figure S1 in the Supplementary Materials, show that our model captures the mean behaviour of the system components in agreement with fluorescence readings in experiments [11,12]. The plots also show that the active transcription factor DiPhoBpp concentration and the active promoter pPhoAa concentration are functions of the external $P_{i}$ concentration.

## 3. Stochastic Dynamics of Chemical Reaction Networks

Deterministic and stochastic simulations reflect the two facets of the Chemical Reaction Networks (CRNs) with respect to the underlying mass action dynamics. Because a stochastic simulation trajectory represents one of the many possible “realisations” of the system, it can capture the fluctuations in species numbers and possible extinctions that may arise due to low species numbers. The deterministic simulations, on the other hand, reflect the mean behaviour of the network. They thus do not capture noise or extinction events. Consequently, the stochastic simulations, at their limit of large numbers, overlap with the deterministic simulations. The stochastic simulation plots in Figure 2 exemplify this idea in comparison with the deterministic simulations. The simulations are performed with Gillespie’s stochastic simulation algorithm (SSA) [35] (see Supplementary Materials).

## 4. Coherence in Response to Phosphate Concentration

In *E. coli*, the TCS mechanism relays the information on external $P_{i}$ concentration to the genetic level. Thus, the activity level of the transcription factor DiPhoBpp provides an internal representation of the external $P_{i}$ concentration. In agreement with experimental observations, the simulations with the differential equations in [11] show that the mean behaviour of our model of the TCS mechanism in response to external $P_{i}$ concentration remains robust to perturbations in many of the system parameters. As shown in the simulations in Figure 2, the system maintains a certain steady state in accordance with the external $P_{i}$ levels also with the feedback provided by the expression of the TCS components that are regulated by this transcription factor: the increased activity of the transcription factor results in the expression of the transcription factor itself as well as the histidine kinase PhoR. Still, the steady-state level of PhoB dimers remains in equilibrium as a function of only the input signal. This phenomenon is a consequence of the bifunctional role of PhoR, which participates in both phosphorylation and dephosphorylation of its cognate response regulator PhoB [34,36,37]. This dual role of PhoR is a mechanism that enhances signal robustness.

**Figure 3:**
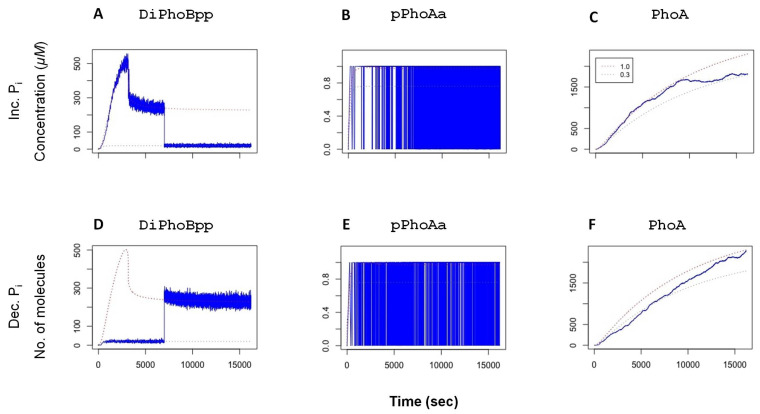
Stochastic time series of simulations. At 7000 simulated seconds, the phosphorylation fold change fc is decreased from 1.0 to 0.3 (top row) for (**A**) DiPhoBpp, (**B**) pPhoAa and (**C**) PhoA. or increased from 0.3 to 1.0 (bottom row) in relation to a change in $P_{i}$ concentration for (**D**) DiPhoBpp, (**E**) pPhoAa and (**F**) PhoA. For comparison, the deterministic trajectories are plotted with dashed lines. The stochastic simulations are scaled down to one-tenth of the *E. coli* volume such that there is a single promoter on a plasmid per simulation. The binding and unbinding effects on the promoter become observable in the plots of the active promoter pPhoAa. An increase in the unbinding events results in the fully painted area in the pPhoAa plot. A decrease introduces gaps to the painted area. The right-most column displays the adjustment of the system’s yield, given by PhoA, in response to the change in external $P_{i}$ levels.

In experiments, it has been shown that the phosphatase activity in the TCS provides a rapid dephosphorylation mechanism that tunes the system when it becomes subject to changes, and thereby restores it to the original state [38]. To verify this observation with our model, we have performed simulations, whereby the system is exposed to a change in external $P_{i}$ after the active transcription factor DiPhoBpp has reached deterministic equilibrium: the model is first instantiated with an autophosphorylation value (fc). In two sets of stochastic simulations, the fc value is then decreased or increased at 7000 simulated seconds, corresponding to a sudden change in the external $P_{i}$ concentration. The stochastic simulation results, depicted in Figure 3, show that after these perturbations, the system tunes its transcription factor levels accordingly. Consequently, the promoter activity and the yield of the system (PhoA) adjust to the modified activity.

## 5. Stochasticity in Promoter Design

As demonstrated by the simulations in Figure 2 and Figure 3, the TCS transcription factor activity, that is, the concentration of the phosphorylated PhoB dimers, serves as a proxy for the external $P_{i}$ concentration, given by the fc value. The resulting active transcription factor signal activates the promoter. This signal feeds back in the form of the expression of the TCS components and other proteins, e.g., PhoA. This process thus provides the specific adaptation of gene expression dependent on the external $P_{i}$ response stimuli by providing the appropriate promoter activity. In this setting, the promoter activity, pPhoAa and pPhoBa, is proportional to the transcription factor DiPhoBpp affinity to the promoter as well as the concentration of DiPhoBpp.

The binding rate of the active transcription factor to the promoter is determined by the specific nucleotide sequence of the promoter, which also determines how long the promoter remains bound, thus activated, after binding. A mutation in a single nucleotide can result in a drastic modification of the binding and unbinding rates [10,39,40]. Many applications in synthetic biology are based on exploiting such mechanisms by introducing random mutations to the promoter sequence and, this way, generating libraries of promoters with desired strengths.

**Figure 4:**
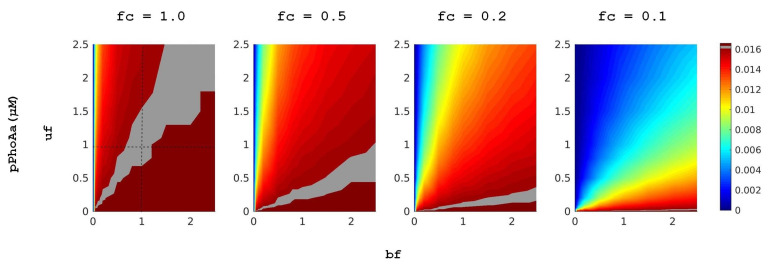
Heatmaps for the activity of various promoter designs as in [11]. The heatmaps are ordered according to the external $P_{i}$ concentration given by the fold changes fc applied to the PhoR autphosphorylation reactions. The left most column with 1.0 as the fc value is the starvation condition with 0 μM external $P_{i}$. Each heatmap scans 100 simulations by applying 10 different fold change values to the promoter binding rates, given with bf in r16 and r18, as well as 10 different fold change values to the promoter unbinding rates, given with uf in r17 and r18 in the full model in the Supplementary Materials. The heatmaps display the resulting steady-state levels of the active promoter pPhoAa in deterministic ordinary differential equation simulations. The intersection of the dashed lines in the left column delivers the experimentally observed regime reported in [11]. The levels of this regime that display the starvation response are highlighted in all the heatmaps.

In [11], after an evaluation of the model with respect to the experimental data, we have performed a class of deterministic simulations to explore the effect of variations in promoter strength on protein expression. In these simulations, we have measured the PhoA protein yield of the system in conditions of different external $P_{i}$ concentrations. For each external $P_{i}$ concentration, we have scanned 100 different promoter designs by varying DiPhoBpp binding rates, associated with reactions r16 and r18, and unbinding rates, associated with reactions r17 and r19. For this, we have applied the dimensionless scaling factors uf and bf, respectively, in a spectrum of 10 different values for each, thus modulating the binding and unbinding rates in a spectrum from 0.25- to 2.5-fold of the control values. These variations model the modulations due to specific promoter nucleotide sequence, whereby the number of active TF can vary within several orders of magnitude in the case of PhoBR TCS. Thus, small changes in promoter strength can affect gene expression and, consequently, $P_{i}$ intake. A representative heatmap for these simulations that shows the mean promoter activity pPhoAa as well as the emerging trend due to the variations in fold change values as in [11] is depicted in Figure 4. These simulations show that to obtain the starvation response in the conditions with higher external $P_{i}$ concentration, promoter binding rates need to be increased and unbinding decreased via the appropriate nucleotide sequence.

Cells with the same genetic make up can exhibit phenotypic variation in the expression of their different proteins. Some of this variation is attributed to noise that is extrinsic to the protein expression machinery, characterised as the fluctuations in other cellular components. On the other hand, the biochemical process of gene expression can be a source of significant intrinsic noise that results in loss of coherence in the output signal, especially in the context of low molecule numbers [15,41]. The differential equation simulations capture the mean deterministic behaviour that would emerge within a population that employs such mechanisms. However, they do not capture the extent of fluctuations in individuals and the possible variation within the population.

To detect the intrinsic noise in gene expression in the system described by our model, we have run a set of repeated stochastic simulations under three external $P_{i}$ concentration conditions, modelled as fc values 1.0, 0.3 and 0.1. In accordance with the simulations in [11], we have then varied the binding and unbinding rates of the transcription factor and the promoter by applying the factors bf∈{0.5,1.0,1.5} for the reactions r16, r18 and uf∈{0.5,1.0,1.5} for the reactions r17, r19. Here, note that although 5 simulations may seem too small a sample, given that each of the simulations contains an abundance of sample points, these simulations provide a sufficient pool for assessing the noise in the system.

In accordance with Figure 4, in these simulations, we observe that a concomitant increase in binding rates and a decrease in unbinding rates provide higher mean levels of the active promoter, given with pPhoAa. However, a fine-tuned balance of these rates is required for the system in order not to overshoot the mean gene expression levels in lower external $P_{i}$ concentration conditions, given with fc values closer to 1.0. From a biological point of view, such an overshoot can have implications on function very much like a constitutive promoter or the deletion of the promoter would introduce a selective pressure on the organism.

To assess the noise in the system, we resorted to the commonly-used metrics based on stationary mean and variance to determine cell-to-cell variability [21,42]: (i) the stationary variance or coefficient of variation (CV) of the probability distribution of mRNA or protein copy number per cell, CV =σ/μ, i.e., the ratio of the standard deviation, σ, to the mean, μ, which is a “straightforward estimate for the overall population variability” [21]; (ii) the noise strength or Fano factor (FF), FF $=\sigma^{2}/\mu$, which “reports the fold change in CV${}^{2}$ with respect to the Poisson process” [8], FF $={\mathrm{CV}}^{2}/{\mathrm{CV}}_{\mathrm{Poisson}}^{2}$. That is, it indicates the deviation of regulated gene expression from the Poissonian distribution generally associated with the constitutive gene expression [8,19,22,23].

**Figure 5:**
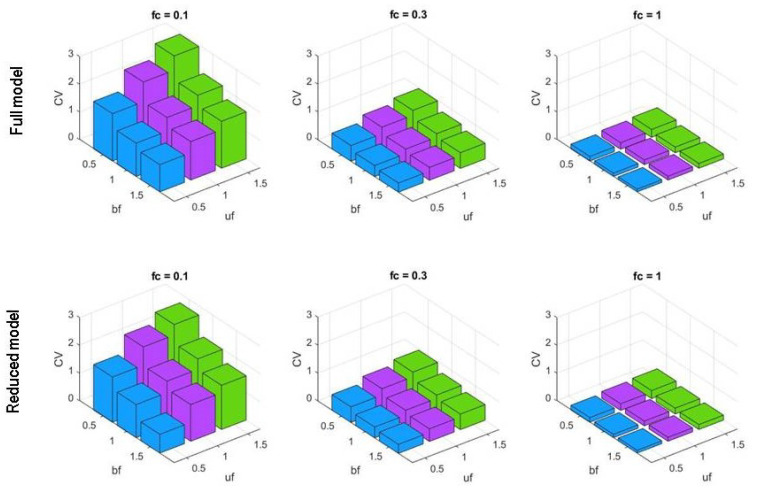
Bar graphs of pPhoAa coefficients of variation obtained by varying uf and bf with different external $P_{i}$ concentrations, fc ∈{0.1,0.3,1.0}, in both full and reduced models. The lowest intrinsic noise levels correspond to low unbinding factors and high binding factors for the pPhoAa promoter.

As shown in Figure 5 and Figure S2, the measurements of these values for pPhoAa on simulations with our model, which we call *the full model*, demonstrate an appreciable increase in noise with an increase in unbinding rates (uf) and a decrease in noise with an increase in binding rates (bf) together with a considerable decrease with an increase in fc. In all the external $P_{i}$ concentration regimes, these values for pPhoAa increased together with an increase in the unbinding factor uf. Similarly, an increase in the binding factor bf consistently reduced the noise in promoter activity in all the regimes. The highest fidelity in the promoter activity signal was obtained with bf=1.5 and uf=0.5. For the mRNAa signal, however, as depicted in Figure S3, a significant consistent change in CV value as a result of a change in unbinding factor uf is observable only with fc values 0.3 and 0.1, corresponding to higher $P_{i}$ concentrations. Similarly, an increase in binding factor bf resulted in a decrease in noise in terms of CV for the mRNAa signal only at higher $P_{i}$ concentrations.

The highest fidelity in terms of noise is obtained with bf=1.5 and uf=0.5. However, a more optimal design that prevents an overshoot in promoter activity is obtained with uf>0.5. This indicates that there is a trade-off between the design of promoters that are capable of exhibiting a starvation response in higher external $P_{i}$ concentration conditions and the noise in the synthetic system.

## 6. Reducing the PhoBR TCS Model

More refined models often lead to highly complex systems with large numbers of variables and parameters. In some cases, a high degree of complexity can be managed by increasingly accurate computational tools, but, in most cases, complexity sets barriers to the model analysis. Reduced models, for their part, lower the complexity level by abstracting away from many system parameters and removing variables and reactions with little impact upon the system outcomes. This way, they make the fitting procedure more feasible. Moreover, they allow us to establish a more straightforward relationship between the system variables and experimental measurements by removing the species that are difficult to measure. With the use of adequate methods, the resulting model provides a manageable instrument to study the features of the specific system.

**Figure 6:**
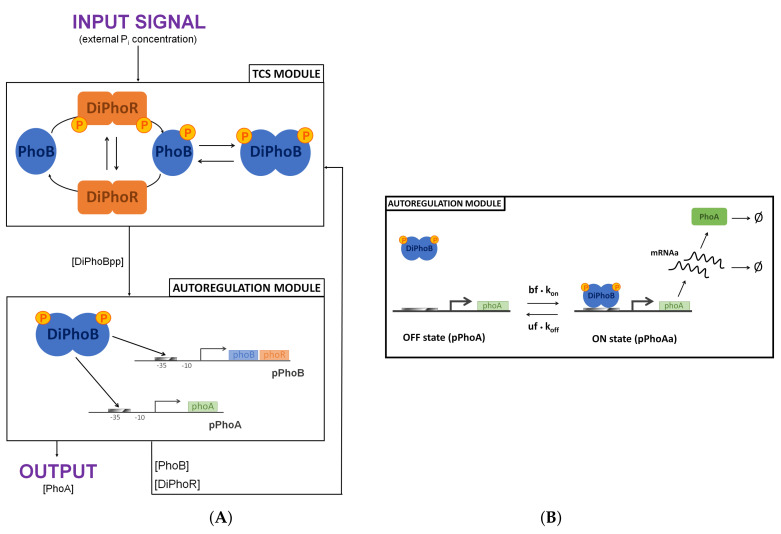
The two interconnected regulatory modules of the PhoBR TCS. (**A**) The TCS module includes the interactions between the histidine kinase and the response regulator, that is, PhoR autophosphorylation and the reverse reaction, PhoB phosphorylation by phosphotransfer and PhoB dephosphorylation by PhoR acting as a phosphatase. The full model takes into account the PhoR and PhoB dimeric nature together with the intermediate complexes formed by interacting proteins. In contrast, the reduced model abstracts away from the dimeric structures of TCS components and neglects the protein complexes. The autoregulation module receives DiPhoBpp concentration as input to transcription control. It includes transcription and translation processes for the expression of *phoBR* and *phoA* genes and protein degradation/dilution. The reduced model represents transcription and translation in a single step. The system output can be quantified by the active transcription factor (DiPhoBpp) level as it regulates the gene products of the PhoBR operon, including PhoB and PhoR as well as others [29]. In turn, PhoB and PhoR concentrations are inputs for the TCS module together with the external $P_{i}$ concentration. (**B**) Close-up view of the two-state promoter model for the *phoA* promoter in the autoregulation module. The dimensionless scaling factor uf and bf are applied to DiPhoBpp binding rate $k_{on}$ (μm${}^{-1}$ s${}^{-1}$), associated with reactions r16 and r18, and unbinding rate $k_{off}$ (s${}^{-1}$), associated with reactions r17 and r19, to scan different binding affinities identified by the dissociation constant $K_{d}=k_{off}/k_{on}^{0}$; $k_{on}$ depends on DiPhoBpp concentration, $k_{on}=k_{on}^{0}\;\left[\mathrm{DiPhoBpp}\right]$.

By applying specific reduction procedures that eventually lower the number of variables in our analysis, we obtained a reduced model of the PhoBR TCS, which is conservative in terms of the response dynamics and steady-state behaviour. As shown in Figure 6, the chemical reaction network setup for the reduced model can be divided into two interconnected regulatory modules: the TCS module and the autoregulation module. The CRN module outputs the phosphorylated PhoB dimer, which is the input of the autoregulation module, which, in return, feeds back to the TCS module. The reactions of this CRN are detailed in the Supplementary Materials as well as its parameterisation following [11]. We have mapped this CRN by using the standard translation based on stoichiometries and the law of mass action. The result is a 10 ordinary differential equation (ODE) model, which can be reduced to 8 ODEs together with 2 mass conservation laws related to the promoter concentrations. We have implemented the ODE model in MATLAB and run numerical simulations with the ode15s and ode23s solvers.

We have performed a reduction of the autoregulation module via a *quasi-steady-state approximation* (QSSA) from singular perturbation theory (see in [43] for a mathematically rigorous treatment). This standard procedure is based on separation of timescales, according to which the system is partitioned into fast and slow components. The quasi-steady-state assumption is often used in the specific case of gene expression, where there are low mRNA and gene copy numbers and an abundance of proteins [18,44,45,46]. Specifically, mRNA concentration is assumed to exhibit *fast* dynamics in comparison with the other *slow* CRN species, which sense mRNA steady-state level (see the Supplementary Materials for further details). Therefore, in our reduced model, transcription and translation are represented in a single step. As a general feature, this approximation converges to the original model at steady state, while exhibiting a time error over the transient behaviour. Specifically, QSSA speeds up the system response time.

The reduced model abstracts away from PhoR homodimeric structure. That is, we reduce the histidine kinase to a two-state variable:

- the inactive state, in which PhoR can either autophosphorylate or act as a phosphatase dephosphorylating PhoBp, and
- the active state, PhoRp, in which the histidine kinase acts as a phosphotransferase by moving a phosphoryl group to PhoB.

In contrast, the detailed model explicitly represents PhoR dimeric nature. Two active states are considered—the phosphorylated homodimer DiPhoRpp and the heterodimer DiPhoRp, both having phosphotransferase activity. This reduction does not alter the system at the steady state. However, it affects the system dynamics by abolishing PhoBp and DiPhoBpp initial peaks in the time series dynamics.

Further reduction of the detailed model consists in neglecting the intermediate species, i.e., the protein complexes that result from the interaction between PhoR and PhoB in all their forms. This is a common procedure adopted in phosphorelays and TCS models. The absence of the intermediates affects the system dynamics and consequently delays the peaks that characterise the species in the detailed model and increases their amplitude. The peaks plummet rather than slowly decreasing. This observation suggests that the role of the intermediates consists in delaying both accumulation and consumption. On the other hand, it has been formally proven in [47] that, if synthesis and degradation of all *core* species are considered, the absence of intermediate species does not affect the steady-state features of the system. In the PhoBR TCS case, two mass conservation laws hold for *phoBR* and *phoA* promoters. Therefore, omitting the intermediate species affects the steady-state level of the system output by enhancing it. By doubling PhoBp dephosphorylation rate constant $k_{3}$, the reduced model matches the PhoBp and DiPhoBpp steady-state levels.

Overall, the reduced model is a valuable tool for analysing the deterministic steady-state behaviour of the PhoB/PhoR TCS due to its agreement with the full model. While preserving an accurate description of the system’s biochemical mechanisms, this model allows us to deal with model complexity. Indeed, it is easier to handle from an analytical point of view. In the following section, we show that this model can retain an accurate predictive capacity also in terms of intrinsic noise and signal fidelity. However, the reduction comes with the cost of information loss on the phenotypic detail in time series dynamics, depicted in Figure S1 in the Supplementary Materials, as well as the explicit representation of the excluded model species.

## 7. Signal Fidelity and Noise in the Reduced Model

To assess the signal fidelity of the reduced model, we have performed stochastic simulations and computed the CV and FF values. Varying the TF binding and unbinding rates leads to different steady states and response times. Therefore, we have chosen the deterministic system state at t = 16,200 s as a starting point for the stochastic simulations; at this time point, the system has reached an equilibrium in all the cases under investigation. We ran all stochastic simulations for a time period that is sufficient to provide an abundant sample of time points, i.e., 1000 s. We considered different environmental conditions, ranging from $P_{i}$ starvation to $P_{i}$ abundance. To represent various input stimuli, as before, we applied a fold change fc ∈{0.1,0.3,1} to the rate of PhoR autophosphorylation activity. An fc =1 corresponds to the system starvation response when external $P_{i}$ concentration is 0 μm whereas a decrease in fc represents an increase in external $P_{i}$ level.

Again, to model different synthetic promoters with various strengths, we applied the scaling factors uf ∈{0.5,1,1.5} and bf ∈{0.5,1,1.5} to DiPhoBpp binding and unbinding rates, respectively. These factors modulate the active transcription factor affinity to the two promoters: pPhoA and pPhoB. The default values bf=1 and uf=1 correspond to the control model with parameters in Table S1 Variations from the default values can indicate both stronger and weaker synthetic promoters. We ran 5 stochastic simulations for each of the 27 cases that result from combining different uf, bf, and fc, which provided an abundant sample of time points. In the control model, calibrated in $P_{i}$ starvation conditions at a single-cell scale at 0.1 fold of the *E. coli* volume, the initial promoter numbers are set to 10 for each mRNA, and *E. coli* volume is set to 1 μm${}^{3}$.

Even though this model has a lower level of molecular detail than the full model, it provides comparable insights into the dependence of intrinsic noise on promoter strength. In particular, Figure 5 and Figure S2 show that

- pPhoAa CV and FF metric values increase with a decrease in the fold change fc. That is, fluctuations increase with an increase in external $P_{i}$ concentration.
- In the same environmental conditions given by the same fc:
  - pPhoAa metric values decrease with a decrease in the promoter unbinding factor uf. Thus, slow unbinding rates result in a considerable reduction of fluctuations.
  - pPhoAa metric values slightly decrease with an increase in the promoter binding factor bf. Thus, fast binding rates result in a limited reduction of intrinsic noise.

In these simulations, the lowest CV value pair with the lowest uf (=0.5) and the highest bf (=1.5), that is, the strongest promoter among those considered. Therefore, *the highest signal fidelity is given by low unbinding rates and high binding rates*. Overall, even though both binding and unbinding rates affect gene expression fluctuations, an appreciable reduction of intrinsic noise occurs only by employing synthetic promoter designs with low unbinding rates and greater binding rates.

These results confirm the observations with the *full* model for *E. coli* PhoBR TCS. This further supports the soundness of the *reduced* model. However, reducing a detailed model always leads to losing some information. The mRNA species corresponding to PhoA expression (mRNAa) does not appear in our model. This is due to the reduction of the autoregulation module using the separation of timescales through a quasi-steady-state approximation. On the other hand, the full model enables us to quantify the fluctuations for the mRNA molecules.

## 8. Discussion

We have presented an analysis of the noise in the biochemical machinery in the phosphate economy of *E. coli* based on stochastic simulations with two models that differ in molecular detail. Our analysis builds on previous work in [11,24] with a computer-aided design point of view for synthetic biology applications, whereby developed technologies are envisioned to go through an *in silico* verification before they are built in the laboratories. In this respect, our models provide the means for assessing the dynamic phenotype of promoters with various strengths. For the case of PhoBR TCS, in high external $P_{i}$ levels, a starvation response results from synthetic promoters with high binding rates and low unbinding rates. However, stronger promoters may also result in “malfunctioning” as they would deprive the organisms of metabolic resources by shifting them to introduce selective pressure.

A related mechanism that has been investigated in relation to noise in the gene expression machinery is the competition for specific transcription factors between multiple promoter sites as well as decoy sites [48,49,50]. Although prokaryotic TFs can differentiate between regulatory and decoy binding sites rather easily due to the binding free energy of their targets [51,52], these sites can play a noise buffering role for the system [48,49,50]. In a model, the availability of such sites will have the same effect as a reduction in the binding rate of TF to the target promoter. A more detailed analysis of such sites in the context of PhoBR TCS by simulations will involve the inclusion of the corresponding reactions, which we leave for future work.

Fine-tuning for the right promoter strength that matches the design specifications of the engineered applications can involve a consideration of many biochemical requirements. The signal fidelity and noise relationship in synthetic biology designs is one such parameter that may determine the healthy functioning of the engineered organism. In this respect, our analysis illustrates that even simpler models as the reduced model we have proposed are informative for the key phenotypic properties of these systems. In synthetic biology applications, where these models serve as blueprints of the designed systems, the models can be used as virtual labs within a computer-aided design setting. The models can be further interrogated by pairing sets of model parameters and experimental measurements with sets of synthetic promoter designs.

The CV and the Fano factor (FF) we use in our analysis are measures that are commonly used to quantify noise in gene expression. However, a consensus on the merits of a measure or another is lacking in the literature. Although CV is a dimensionless value that is beneficial for comparisons that involve widely different means, it may fail to provide a consistent quantification of noise for the transcription and translation events as the accuracy of mean increases with the sample size, whereas CV may remain unaffected.

FF is commonly used as an alternative measure for biological processes since it takes the Poissonian process as the reference point. It is thus indicative of a deviation from this baseline (see, e.g., [22,53,54]). When the FF is smaller than 1, the system is considered under-dispersed. It is considered over-dispersed when it is greater than 1. The transcription events for a constitutive promoter are characterised by a Poisson distribution, whereas the number of proteins expressed for every mRNA molecule follows a geometric distribution [55]. The transcription and translation processes are characterised as bursts, driven by a promoter that switches between active and inactive states as a consequence of the regulatory signals. Because bursty transcription causes higher noise than Poissonian transcription [56], FF provides a quantification of the dispersion in relation to this baseline.

Consistent with the considerations above, our results indicate that the FF for mRNA for PhoA expression, mRNAa, has three different phases: Poissonian, sub-Poissonian and super-Poissonian phases, within the interval of 0.9 to 1.34 (Figure S3). However, the quantification of noise by FF does not follow a regular trend for the fc=0.1, and it is affected by the mean (Figure S4). The deviation in the trend may be due to multivariate random processes underlying gene expression. In this respect, in [57], Paulsson argues that FF works well for univariate discrete random processes, where the variance remains proportional to the mean with a proportionality constant that reflects the overall nature of the process, whereas multivariate random processes may render FF misleading. Although very small FF values for gene expression are not intuitive, in [53], Sharon et al. report FF values of the order $10^{-1}$.

The promoter activity signal, given by pPhoAa in our model, provides a consistent quantification of noise for both CV and FF measures. With a single promoter pPhoA, it is impossible for the FF to take a value greater than 1 for the active promoter pPhoAa. This aspect renders the Poisson reference of FF irrelevant for the analysis on pPhoAa with respect to the noise trend in the gene expression machinery. This trend, which can be observed in the “Stochastic Simulation Time Series Samples” section in the Supplementary Materials, is clearly identified also for a large spectrum of values show in Figure S5.

More general questions related to noise with an impact on synthetic biology applications include those such as “where does noise arise in the cell?” and “by what means do regulatory networks attenuate this noise?” [25]. Elowitz et al. [15] identified the two sources of fluctuations in gene expression by differentiating stochasticity into intrinsic and extrinsic; intrinsic noise highly depends on promoters. Swain et al. [17] continued the analysis and presented a mathematical framework for interpreting experimental noise measures. They suggested that transcription governs the intrinsic noise when the translation efficiency is high, i.e., when translational bursting occurs. This is believed to be a common property in *E. coli*, by which translation of proteins from mRNA occurs in pulses. In this respect, Özbudak et al. [19] investigated to what extent intrinsic noise is affected by transcription and translation rates. Their work predicts an inverse correlation between noise and the rate of transcription. On the other hand, noise is slightly affected by the rate of translation because of translational bursts. The authors identified transcription as the main source of intrinsic noise in prokaryotes. Their results support the predictions previously formulated in [18], where Thattai and van Oudenaarden proposed a simple analytical model of transcription and translation as well as regulatory gene interactions and analysed the moments of mRNA and protein distributions. Their conclusions proved that cell-to-cell variability can be governed by varying genetic parameters.

In addition to transcriptional and translational control, there are several ways to modulate noise levels in a genetic circuit: the presence of both negative and positive autoregulation [18,44,58,59], the regulation of gene copy number [21], and the fluctuations transmitted by upstream genes [60]. Our results align with these results as we analyse transcriptional control exercised by synthetic promoters with different strengths. Our results demonstrate how the signal propagated in the PhoBR TCS resulting in the TF activity and the consequent TF binding affinity affect the noise levels in the system.

Another factor that can impact the variability in gene expression, thus the noise levels in the system, is the number and location of promoter sites, the occurrence of DNA looping, and the presence of multiple competing or cooperative transcription factors. Sanchez et al. implemented a theoretical approach based on the master-equation of stochastic gene expression to systematically study these different promoter architectures [22]. By analyzing the FF associated with the mRNA and protein distributions, the authors showed that promoter strength is central in determining cell-to-cell variability. For both simple activation and repression, strong promoters are expected to produce large noise levels due to slow promoter state fluctuations. On the other hand, weak promoters associated with fast TF dissociation rates result in smaller mRNA fluctuations.

Munsky et al. found that long periods of both promoter activity and inactivity lead to high FF values and bimodal mRNA distribution. Specifically, three distinct modes of phenotypic variability are identified by experimental and computational analyses; varying binding and unbinding rates ensures the switch between two different behavioural classes. Fast binding rates if compared to mRNA degradation and unbinding rates result in low FF levels and a graded unimodal distribution similar to that of constitutive gene expression. Similarly, our analysis on *phoA* promoter suggests that mutations in promoter sequences resulting in strong promoters with low dissociation constants provide lower noise levels and thus higher signal fidelity in all $P_{i}$ environmental conditions. In particular, variations of TF unbinding rate lead to a stronger decrease in fluctuations. In this respect, So et al. provided supporting evidence for the control of promoter unbinding rate to be a common pattern in the regulation of mRNA expression [23,61].

The role of slow unbinding rates in alleviating intrinsic noise is found also for titration-based oscillators in [62]. Nevertheless, in our analysis, this is not immediately clear for mRNA distribution: for $P_{i}$-repleted conditions, the trend is more easily detectable if compared to phosphate starvation conditions. Note that the modular model we have proposed for the PhoB/PhoR TCS extends the two-state promoter model by including the TCS phosphorylation cycle, implementing a positive autoregulation mechanism. Therefore, the effect of the TCS context of the gene expression machinery and the consequent increased model complexity reflects on the results of our analysis.

In a more recent work [8], Jones et al. experimentally tested the predictions on the impact of promoter architecture on intrinsic noise by using a collection of promoters with different strengths and the mRNA fluorescence in situ hybridisation (FISH). This way, the authors directly related changes in promoter architecture to changes in specific genetic knobs. Our work moves in this direction as our results imply that synthetic promoters can be exploited as control tools for filtering out intrinsic noise, thus ensuring a certain level of signal fidelity.

## 9. Conclusions

Inherent fluctuations in gene expression can hamper the healthy functioning of synthetic biology devices by causing a loss of coherence in the system’s output. The relationship between signal transmission fidelity and intrinsic noise relies upon a delicate balance. Even a small change in the nucleotide sequence of a promoter, that is, a small variation in the promoter strength, can increase the noise level in gene expression, thus reducing output fidelity to the input signal. Within a computational framework, we have simulated the effect of synthetic promoters with different strengths on *E. coli* phosphate response to investigate the interplay between signal fidelity and intrinsic noise. Both the *full* and reduced model associate the highest fidelity in the promoter activity level with strong promoters characterised by low unbinding rates. Our results suggest that synthetic promoters display a trade-off between providing a starvation response and maintaining low noise levels. Overall, our models provide blueprints for designing more stable synthetic devices to suppress fluctuations in the context of *E. coli* phosphate signal transduction. Generally, synthetic biology benefits from the quantitative understanding of the complex networks underlying cellular and molecular physiology. In this regard, new insights into $P_{i}$ intake mechanisms in *E. coli* should take synthetic biotechnology applications such as those for wastewater treatment a step further.

The PhoBR TCS that is central to our study belongs to one of the largest and most diverse families of sensory components in biology. The TCS machinery, which relays the signal on environmental changes to the genetic components for tuning protein expression, is preserved in all life. Because of their highly conserved and well-characterised structure, TCS are suitable candidates for engineering intervention. Our work provides a template for studying other signalling circuits with TCS integrated to them. In this respect, the modular makeup of our models makes it possible to plug them into other models with similar features. A compositional setting where models can be integrated as building blocks of larger systems will likely contribute to our understanding of the machinery of life for engineering living technologies.

## Supplementary Materials

The following are available online at https://www.mdpi.com/article/10.3390/biology10080724/s1, Text 1: Stochastic Simulation Algorithm (SSA), Text 2: The Full Model, Text 3: Quasi-Steady-State Approximation, Text 4: The Reduced CRN, Table S1: Parameter values and initial conditions for the reduced PhoBR TCS model, Table S2: pPhoAa CV for the full model, Table S3: pPhoAa CV for the reduced model, Table S4: pPhoAa FF for the full model, Table S5: pPhoAa FF for the reduced model, Table S6: mRNAa CV for the full model, Table S7: mRNAa FF for the full model, Figure S1: Experimental data from fluorescence readings and deterministic time-series plots, Figure S2: Bar graphs of pPhoAa Fano factors, Figure S3: Bar graphs of CV and FF values of mRNAa, Figure S4: Bar graphs of mean mRNAa levels at equilibrium, Figure S5: Heatmaps of pPhoAa Fano factors for pPhoAa, Figure S6: Stochastic Simulation Time Series Samples

## References

[1] Wilson E.H., Groom J.D., Sarfatis M.C., Ford S.M., Lidstrom M.E., Beck D.A.C. (2021). A Computational Framework for Identifying Promoter Sequences in Nonmodel Organisms Using RNA-seq Data Sets. ACS Synth. Biol..

[2] Pedone E., de Cesare I., Zamora-Chimal C.G., Haener D., Postiglione L., Regina A.L., Shannon B., Savery N.J., Grierson C.S., di Bernardo M. (2021). Cheetah: A Computational Toolkit for Cybergenetic Control. ACS Synth. Biol..

[3] Mısırlı G., Nguyen T., McLaughlin J.A., Vaidyanathan P., Jones T.S., Densmore D., Myers C., Wipat A. (2019). A Computational Workflow for the Automated Generation of Models of Genetic Designs. ACS Synth. Biol..

[4] Matsuura T., Hosoda K., Shimiz Y. (2018). Robustness of a Reconstituted *Escherichia coli* Protein Translation System Analyzed by Computational Modeling. ACS Synth. Biol..

[5] Marchisio M.A. (2015). Computational Methods in Synthetic Biology. Methods in Molecular Biology.

[6] Khalil A.S., Collins J.J. (2010). Synthetic biology: Applications come of age. Nat. Rev. Genet..

[7] Mosa K., Saadoun I., Kumar K., Helmy M., Dhankher O. (2016). Potential biotechnological strategies for the cleanup of heavy metals and metalloids. Front Plant Sci..

[8] Jones D.L., Brewster R.C., Phillips R. (2014). Promoter architecture dictates cell-to-cell variability in gene expression. Science.

[9] Wang Y., Wang H., Wei L., Li S., Liu L., Wang X. (2020). Synthetic promoter design in *Escherichia coli* based on a deep generative network. Nucleic Acids Res..

[10] Jensen P.R., Hammer K. (1998). The sequence of spacers between the consensus sequences modulates the strength of prokaryotic promoters. Appl. Environ. Microbiol..

[11] Uluşeker C., Torres-Bacete J., García J.L., Hanczyc M.M., Nogales J., Kahramanoğulları O. (2019). Quantifying dynamic mechanisms of auto-regulation in *Escherichia coli* with synthetic promoter in response to varying external phosphate levels. Sci. Rep..

[12] Torres-Bacete J., Luís García J., Nogales J. (2021). A portable library of phosphate-depletion based synthetic promoters for customable and automata control of gene expression in bacteria. Microbial Biotechnology.

[13] Youk H. (2020). AP3162: Gene-Regulatory Circuits: Stochastic Dynamics. https://www.youklab.org/teaching/QBio/Lecture3_notes_Qbio.pdf.

[14] Swain P.S. (2016). Lecture notes on stochastic models in systems biology. arXiv.

[15] Elowitz M.B., Levine A.J., Siggia E.D., Swain P.S. (2002). Stochastic gene expression in a single cell. Science.

[16] Raj A., Van Oudenaarden A. (2008). Nature, nurture, or chance: Stochastic gene expression and its consequences. Cell.

[17] Swain P.S., Elowitz M.B., Siggia E.D. (2002). Intrinsic and extrinsic contributions to stochasticity in gene expression. Proc. Natl. Acad. Sci. USA.

[18] Thattai M., Van Oudenaarden A. (2001). Intrinsic noise in gene regulatory networks. Proc. Natl. Acad. Sci. USA.

[19] Özbudak E., Thattai M., Kurtser I., Grossman A.D., Van Oudenaarden A. (2002). Regulation of noise in the expression of a single gene. Nat. Genet..

[20] Raser J.M., O’Shea E.K. (2004). Control of stochasticity in eukaryotic gene expression. Science.

[21] Raser J.M., O’shea E.K. (2005). Noise in gene expression: Origins, consequences, and control. Science.

[22] Sanchez A., Garcia H.G., Jones D., Phillips R., Kondev J. (2011). Effect of promoter architecture on the cell-to-cell variability in gene expression. PLoS Comput. Biol..

[23] Munsky B., Neuert G., Van Oudenaarden A. (2012). Using gene expression noise to understand gene regulation. Science.

[24] Kahramanoğulları O., Uluşeker C., Hancyzc M.M. (2019). Stochastic Mechanisms of Information Flow in Phosphate Economy of *Escherichia coli*. Numerical Computations: Theory and Algorithms NUMTA 2019. Lecture Notes in Computer Science.

[25] Rao C.V., Wolf D.M., Arkin A.P. (2002). Control, exploitation and tolerance of intracellular noise. Nature.

[26] Harris R.M., Webb D.C., Howitt S.M., Cox G.B. (2001). Characterization of PitA and PitB from *Escherichia coli*. J. Bacteriol..

[27] Gardner S.G., Johns K.D., Tanner R., McCleary W.R. (2014). The PhoU protein from *Escherichia coli* interacts with PhoR, PstB, and metals to form a phosphate-signaling complex at the membrane. J. Bacteriol..

[28] Carmany D.O., Hollingsworth K., McCleary W.R. (2003). Genetic and biochemical studies of phosphatase activity of PhoR. J. Bacteriol..

[29] Gardner S.G., McCleary W.R. (2019). Control of the phoBR Regulon in *Escherichia coli*. EcoSal Plus.

[30] Ashenberg O., Keating A.E., Laub M.T. (2013). Helix bundle loops determine whether histidine kinases autophosphorylate in cis or in trans. J. Mol. Biol..

[31] Peterson C.N., Mandel M.J., Silhavy T.J. (2005). *Escherichia coli* starvation diets: Essential nutrients weigh in distinctly. J. Bacteriol..

[32] Vuppada R.K., Hansen C.R., Strickland K.A., Kelly K.M., McCleary W.R. (2018). Phosphate signaling through alternate conformations of the PstSCAB phosphate transporter. BMC Microbiol..

[33] Torriani-Gorini A. (1996). History of the Pho System. Regulation of Gene Expression in Escherichia coli.

[34] Shinar G., Milo R., Matinez M.R., Alon U. (2007). Input output robustness in simple bacterial signaling systems. Proc. Natl. Acad. Sci. USA.

[35] Gillespie D.T. (1977). Exact stochastic simulation of coupled chemical reactions. J. Phys. Chem..

[36] Miyashiro T., Goulian M. (2008). High stimulus unmasks positive feedback in an autoregulated bacterial signaling circuit. Proc. Natl. Acad. Sci. USA.

[37] Tiwari A., Ray J.C.J., Narula J., Igoshin O.A. (2011). Bistable responses in bacterial genetic networks: Designs and dynamical consequences. Math. Biosci..

[38] Mukhopadhyay A., Gao R., Lynn D.G. (2004). Integrating input from multiple signals: The VirA/VirG two-component system of *Agrobacterium tumefaciens*. Chembiochem.

[39] Hawley D.K., McClure W.R. (1983). Compilation and analysis of *Escherichia coli* promoter DNA sequences. Nucleic Acids Res..

[40] Jensen P.R., Hammer K. (1998). Artificial promoters for metabolic optimization. Biotechnol. Bioeng..

[41] Leveau J.H., Lindow S.E. (2001). Predictive and interpretive simulation of green fluorescent protein expression in reporter bacteria. J. Bacteriol..

[42] Paulsson J. (2004). Summing up the noise in gene networks. Nature.

[43] Segel L.A., Slemrod M. (1989). The quasi-steady-state assumption: A case study in perturbation. SIAM Rev..

[44] Czuppon P., Pfaffelhuber P. (2018). Limits of noise for autoregulated gene expression. J. Math. Biol..

[45] Bokes P., King J.R., Wood A.T., Loose M. (2012). Multiscale stochastic modelling of gene expression. J. Math. Biol..

[46] Ball K., Kurtz T.G., Popovic L., Rempala G. (2006). Asymptotic analysis of multiscale approximations to reaction networks. Ann. Appl. Probab..

[47] Feliu E., Wiuf C. (2013). Simplifying biochemical models with intermediate species. J. R. Soc. Interface.

[48] Soltani M., Bokes P., Fox Z., Singh A. (2015). Nonspecific transcription factor binding can reduce noise in the expression of downstream proteins. Phys. Biol..

[49] Das S., Choubey S. (2020). Tunability enhancement of gene regulatory motifs through competition for regulatory protein resources. Phys. Rev. E.

[50] Dey S., Soltani M., Singh A. (2020). Enhancement of gene expression noise from transcription factor binding to genomic decoy sites. Sci. Rep..

[51] Gerland U., Moroz J.D., Hwa T. (2002). Physical constraints and functional characteristics of transcription factor-DNA interaction. Proc. Natl. Acad. Sci. USA.

[52] Burger A., Walczak A.M., Wolynes P.G. (2012). Influence of decoys on the noise and dynamics of gene expression. Phys. Rev. E.

[53] Sharon E., van Dijk D., Kalma Y., Keren L., Manor O., Yakhini Z., Segal E. (2014). Probing the effect of promoters on noise in gene expression using thousands of designed sequences. Genome Res..

[54] Charles A.S., Park M., Weller J.P., Horwitz G.D., Pillow J.W. (2018). Dethroning the Fano Factor: A Flexible, Model-Based Approach to Partitioning Neural Variability. Neural Comput..

[55] Yu J., Xiao J., Ren X., Lao K., Xie X.S. (2006). Probing Gene Expression in Live Cells, One Protein Molecule at a Time. Science.

[56] Sanchez A., Golding I. (2013). Genetic Determinants and Cellular Constraints in Noisy Gene Expression. Science.

[57] Paulsson J. (2005). Models of stochastic gene expression. Phys. Life Rev..

[58] Becskei A., Serrano L. (2000). Engineering stability in gene networks by autoregulation. Nature.

[59] Hornung G., Barkai N. (2008). Noise propagation and signaling sensitivity in biological networks: A role for positive feedback. PLoS Comput. Biol..

[60] Pedraza J.M., van Oudenaarden A. (2005). Noise propagation in gene networks. Science.

[61] So L.H., Ghosh A., Zong C., Sepúlveda L.A., Segev R., Golding I. (2011). General properties of transcriptional time series in Escherichia coli. Nat. Genet..

[62] Karapetyan S., Buchler N.E. (2015). Role of DNA binding sites and slow unbinding kinetics in titration-based oscillators. Phys. Rev. E.

