# Supplementary material for: Stochastic Simulations as a Tool for Assessing Signal Fidelity in Gene Expression in Synthetic Promoter Design
Source: Biology (Basel). 2021 Jul 29;10(8):724. doi: 10.3390/biology10080724 (PMC8389217; doi:10.3390/biology10080724)
Supplement: Supplementary file 1 [file biology-10-00724-s001.zip › biology-1242537-supplementary.pdf]

Supplementary Materials: Stochastic Simulations as a Tool for Assessing Signal Fidelity in Gene Expression in Synthetic Promoter Design

Elena Righetti [1,‡], Cansu Uluşeker [2,‡] and Ozan Kahramanoğulları [1,‡,*]

1. Stochastic Simulation Algorithm (SSA)

Stochastic simulations with CRNs are commonly performed by using one of the various versions of Gillespie's stochastic simulation algorithm (SSA) [1]. Given an initial state as a vector of species quantities, the algorithm constructs a trajectory of the network with respect to the underlying continuous time Markov chain semantics. At each simulation step, the algorithm performs a Monte Carlo procedure to sample from the probability distribution of the possible reaction instances at that state to pick a reaction and its time. The algorithm updates the state and continues in the same way until the end-time is reached. The simulation terminates after logging the trajectory to a file. As described above, the SSA generates a stochastic simulation trajectory by sequentially sampling a reaction instance one after another from the distribution of available reactions. The time between two reaction instances is obtained by sampling from an exponential distribution, which is a function of the reaction propensities available at that state. Each reaction instance modifies the system state. The algorithm then continues to pick a reaction instance until it reaches the end-time. The algorithm logs the reaction instances, which provides the time series of the simulations.

2. The Full Model

The CRN in [2] that models the auto-regulation mechanism of *E. coli* in response to varying external phosphate concentrations. The time unit of the reactions is in seconds. The unit of the second order reaction rate constants is $M^{-1}s^{-1}$. The fold-change `fc` factor in reactions `r01` and `r03` models the variations in external P_i concentration. The `fc` $= 1.0$ value corresponds to the starvation condition and a lower `fc` value corresponds to a higher external P_i concentration. The binding factor `bf` in reactions `r16`, `r18` and unbinding factor `uf` in reactions `r17`, `r19` are scalar factors. They represent the affinity of the active transcription factor to the promoter region. In the control model, the default values of `bf` $= 1.0$ and `uf` $= 1.0$ are used.

```
reactions
r01 : DiPhoR              ->   DiPhoRp          , 25.3658*fc;
r02 : DiPhoRp             ->   DiPhoR           , 8.1165;
r03 : DiPhoRp             ->   DiPhoRpp         , 25.3658*fc;
r04 : DiPhoRpp            ->   DiPhoRp          , 8.1165;
r05 :   DiPhoRpp + PhoB   ->   DiPhoRpp_PhoB    , 100;
r06 : DiPhoRpp_PhoB       -> DiPhoRpp + PhoB    , 44.9411;
r07 : DiPhoRpp_PhoB       -> DiPhoRp + PhoBp    , 21.3718;
r08 :   DiPhoRp + PhoB    ->   DiPhoRp_PhoB     , 100;
r09 : DiPhoRp_PhoB        -> DiPhoRp + PhoB     , 94.9411;
r10 : DiPhoRp_PhoB        -> DiPhoR + PhoBp     , 21.3718;
r11 :   PhoBp + PhoBp     ->   DiPhoBpp         , 100;
r12 : DiPhoBpp            -> PhoBp + PhoBp      , 24.9411;
r13 :   DiPhoR + PhoBp    ->   DiPhoR_PhoBp     , 100;
r14 : DiPhoR_PhoBp        -> DiPhoR + PhoBp     , 34.9411;
r15 : DiPhoR_PhoBp        -> DiPhoR + PhoB      , 12.95;
r16 :   DiPhoBpp + pPhoA  ->   pPhoAa           , 10000*bf;
r17 : pPhoAa              -> DiPhoBpp + pPhoA   , 1000*uf;
r18 :   DiPhoBpp + pPhoB  ->   pPhoBa           , 10000*bf;
r19 : pPhoBa              -> DiPhoBpp + pPhoB   , 1000*uf;
r20 : pPhoAa              -> pPhoAa + mRNAa     , 0.0540;
r21 : mRNAa               -> mRNAa + PhoA       , 0.0302;
r22 : pPhoBa              -> pPhoBa + mRNAb     , 0.130;
r23 : mRNAb               -> mRNAb + PhoB       , 0.036;
r24 : mRNAb               -> mRNAb + DiPhoR     , 0.0302;
r25 : PhoA                ->                    , 0.0001;
r26 : PhoB                ->                    , 0.0001;
r27 : DiPhoR              ->                    , 0.0001;
r28 : mRNAa               ->                    , 0.0055;
r29 : mRNAb               ->                    , 0.0055;

initial state
0.22 DiPhoR;     0.22 PhoB;     0.0166 pPhoA;     0.0166 pPhoB;
```

3. Quasi-Steady-State Approximation

In chemical reaction networks, one or more species can have an intrinsic faster timescale: mRNA is the fast species in comparison with the proteins in the system. Namely, mRNA reaches its steady-state level more rapidly than the protein concentrations. Given the original differential equation-based description of mRNA concentration behaviour,

$$[\text{mRNAa}]' = \alpha[\text{active promoter}] - \beta[\text{mRNAa}]\ ,$$

mRNA concentration is approximated by the following expression

$$[\text{mRNAa}]_{qss} = \frac{\alpha}{\beta}[\text{active promoter}]\ .$$

In other words, we assume that mRNA concentration "instantaneously reaches the steady state that it would attain if all other variables were constant" [3]. Since mRNA rapidly reaches the equilibrium, the other variables are essentially constant "from mRNA's point of view" [3]. Eventually, replacing mRNA concentration quasi-steady-state approximations in the system equations returns a reduced ODE model.

4. The Reduced CRN

The TCS module includes all the reactions involved in PhoB/PhoR interaction: PhoR dimer autophosphorylation and the reverse reaction, phosphotransfer from phosphorylated PhoR dimer to the response regulator PhoB, and PhoBp by PhoR dimer. It includes phosphorylated PhoB dimerization and the reverse reaction. This module returns phosphorylated PhoB dimer (DiPhoBpp) concentration, which acts as an interface to the autoregulation module.

$$[\text{PhoR}] \xrightarrow{k_1} [\text{PhoRp}] \qquad (r_1)$$

$$[\text{PhoRp}] \xrightarrow{k_1'} [\text{PhoR}] \qquad (r_1')$$

$$[\text{PhoRp}] + [\text{PhoB}] \xrightarrow{k_2} [\text{PhoR}] + [\text{PhoBp}] \qquad (r_2)$$

$$[\text{PhoR}] + [\text{PhoBp}] \xrightarrow{k_3} [\text{PhoR}] + [\text{PhoB}] \qquad (r_3)$$

$$[\text{PhoBp}] + [\text{PhoBp}] \xrightarrow{k_d} [\text{DiPhoBpp}] \qquad (r_d)$$

$$[\text{DiPhoBpp}] \xrightarrow{k_{-d}} [\text{PhoBp}] + [\text{PhoBp}]\ . \qquad (r_{-d})$$

The system input is the external P_i concentration. The growth-limiting condition with low external P_i levels is the high input-signal regime with a high PhoR autophosphorylation rate. To implement varying environmental conditions, we apply a fold change $\text{fc} \in (0, 1\}$ to k_1, i.e., PhoR autophosphorylation rate constant. An fc$= 1$ corresponds to P_i-starvation conditions, whereas an fc $\to 0$ represents increasing external P_i concentrations.

The autoregulation module receives DiPhoBpp concentration as input to transcription control. It includes transcription and translation processes for the expression of *phoBR* and *phoA* genes and protein degradation/dilution. The system output can be quantified by the active transcription factor (DiPhoBpp) level as it regulates the gene products of the PhoBR operon, including PhoB and PhoR as well as others [4]. In turn, PhoB and PhoR concentrations are inputs for the TCS module together with the external P_i concentration.

DiPhoBpp binds the Pho box in pPhoB promoter, which, in turn, activates (pPhoBa) and produces PhoR and PhoB at different rates. Eventually, PhoR and PhoB degrade.

$$[\text{DiPhoBpp}] + [\text{pPhoB}] \xrightarrow{k_4} [\text{pPhoBa}] \quad (r_4)$$

$$[\text{pPhoBa}] \xrightarrow{k_5} [\text{DiPhoBpp}] + [\text{pPhoB}] \quad (r_5)$$

$$[\text{pPhoBa}] \xrightarrow{k_6} [\text{pPhoBa}] + [\text{DiPhoR}] \quad (r_6)$$

$$[\text{pPhoBa}] \xrightarrow{k_7} [\text{pPhoBa}] + [\text{PhoB}] \quad (r_7)$$

$$[\text{DiPhoR}] \xrightarrow{k_{deg}} \emptyset \quad (r_8)$$

$$[\text{PhoB}] \xrightarrow{k_{deg}} \emptyset\,. \quad (r_9)$$

DiPhoBpp binds the Pho box in pPhoA promoter, which, in turn, activates (pPhoAa) and produces PhoA. Eventually, PhoA degrades.

$$[\text{DiPhoBpp}] + [\text{pPhoA}] \xrightarrow{k_{10}} [\text{pPhoAa}] \quad (r_{10})$$

$$[\text{pPhoAa}] \xrightarrow{k_{11}} [\text{DiPhoBpp}] + [\text{pPhoA}] \quad (r_{11})$$

$$[\text{pPhoAa}] \xrightarrow{k_{12}} [\text{pPhoAa}] + [\text{PhoA}] \quad (r_{12})$$

$$[\text{PhoA}] \xrightarrow{k_{deg}} \emptyset\,. \quad (r_{13})$$

The parameter values and initial conditions are obtained from those in [2].

```
reactions
r1 : DiPhoR           -> DiPhoRpp          , 25.3658;
r2 : DiPhoRpp         -> DiPhoR            , 8.1165;
r3 : DiPhoRpp + PhoB  -> DiPhoR + PhoBp    , 21.3718;
r4 : PhoBp + PhoBp    -> DiPhoBpp          , 100;
r5 : DiPhoBpp         -> PhoBp + PhoBp     , 24.9411;
r6 : DiPhoR + PhoBp   -> DiPhoR + PhoB     , 26.4478;
r7 : DiPhoBpp + pPhoA -> pPhoAa            , 10000;
r8 : pPhoAa           -> DiPhoBpp + pPhoA  , 1000;
r9 : DiPhoBpp + pPhoB -> pPhoBa            , 10000;
r10 : pPhoBa          -> DiPhoBpp + pPhoB  , 1000;
r11 : pPhoAa          -> pPhoAa + PhoA     , 0.29651;
r12 : pPhoBa          -> pPhoBa + PhoB     , 0.82727;
r13 : pPhoBa          -> pPhoBa + DiPhoR   , 0.71382;
r14 : PhoA            ->                   , 0.0001;
r15 : PhoB            ->                   , 0.0001;
r16 : DiPhoR          ->                   , 0.0001;

initial state
0.22 DiPhoR;
0.00000004 DiPhoRpp;
0.22 PhoB;
0.00000006 DiPhoBpp;
0.0166 pPhoA;
0.0166 pPhoB;
```

5. Tables

Table S1

Parameter values and initial conditions for the reduced PhoBR TCS model

Rate constant	Fit Value
k_1	$25.3658\ s^{-1}$
k'_1	$8.1165\ s^{-1}$
k_2	$21.3718\ \mu M^{-1}s^{-1}$
k_3	$26.4478\ \mu M^{-1}s^{-1}$
k_d	$100\ \mu M^{-1}s^{-1}$
k_{-d}	$24.9411\ s^{-1}$
k_4, k_{10}	$10^4\ \mu M^{-1}s^{-1}$
k_5, k_{11}	$10^3\ s^{-1}$
k_6	$0.71382\ s^{-1}$
k_7	$0.82727\ s^{-1}$
k_{12}	$0.296510\ s^{-1}$
k_{deg}	$0.0001\ s^{-1}$

(a) **Deterministic rate constants** [2]

Species	Value
$[PhoR]_0$	$0.22\ \mu M$
$[PhoB]_0$	$0.22\ \mu M$
$[PhoRp]_0$	$4 \cdot 10^{-8}\ \mu M$
$[PhoBp]_0$	$6 \cdot 10^{-8}\ \mu M$
$[pPhoA]_0$	$0.0166\ \mu M$
$[pPhoB]_0$	$0.0166\ \mu M$

(b) **Initial concentrations** [2]

Parameter values and initial conditions for the reduced PhoBR TCS model consisting of CRN reactions r_1-r_{13}. The initial concentrations of the species not included in Table (b) are all set to 0 μM. *E. coli* volume is set to 1 μm^3.

Table S2

pPhoAa CV for the full model

fc = 1.0

bf\uf	0.5	1	1.5
0.5	0.1613	0.2338	0.2846
1	0.1241	0.1659	0.2051
1.5	**0.0973**	0.1392	0.1682

fc = 0.3

bf\uf	0.5	1	1.5
0.5	0.5640	0.8048	1.0167
1	0.4030	0.5655	0.7028
1.5	**0.3197**	0.4624	0.5657

fc = 0.1

bf\uf	0.5	1	1.5
0.5	1.7474	2.3892	2.8424
1	1.2249	1.6972	2.0553
1.5	**0.9842**	1.3860	1.7062

Table S3

pPhoAa CV for the reduced model

fc = 1.0

bf\uf	0.5	1	1.5
0.5	0.1689	0.2401	0.2906
1	0.1206	0.1718	0.2113
1.5	**0.1018**	0.1431	0.1754

fc = 0.3

bf\uf	0.5	1	1.5
0.5	0.5579	0.7866	0.9557
1	0.3911	0.5658	0.6866
1.5	**0.3219**	0.4569	0.5585

fc = 0.1

bf\uf	0.5	1	1.5
0.5	1.6326	2.2858	2.6654
1	1.1934	1.6114	1.9831
1.5	**0.6768**	1.3239	1.5927

Table S4

pPhoAa FF for the full model

fc = 1.0

bf\uf	0.5	1	1.5
0.5	0.0254	0.0518	0.0749
1	0.0152	0.0268	0.0404
1.5	**0.0094**	0.0190	0.0275

fc = 0.3

bf\uf	0.5	1	1.5
0.5	0.2414	0.3931	0.5083
1	0.1397	0.2423	0.3306
1.5	**0.0927**	0.1762	0.2424

fc = 0.1

bf\uf	0.5	1	1.5
0.5	0.7534	0.8511	0.8901
1	0.6001	0.7424	0.8087
1.5	**0.4921**	0.6577	0.7444

Table S5

pPhoAa FF for the reduced model

fc = 1.0

bf\uf	0.5	1	1.5
0.5	0.0277	0.0545	0.0779
1	0.0143	0.0287	0.0427
1.5	**0.0103**	0.0201	0.0299

fc = 0.3

bf\uf	0.5	1	1.5
0.5	0.2374	0.3822	0.4774
1	0.1327	0.2425	0.3204
1.5	**0.0939**	0.1717	0.2378

fc = 0.1

bf\uf	0.5	1	1.5
0.5	0.7273	0.8396	0.8769
1	0.5875	0.7220	0.7974
1.5	**0.4783**	0.6368	0.7173

Table S6

mRNAa CV for the full model

fc = 1.0

bf\uf	0.5	1	1.5
0.5	0.3031	0.3566	0.2926
1	0.2994	0.3203	0.3154
1.5	0.3120	0.3169	0.3711

fc = 0.3

bf\uf	0.5	1	1.5
0.5	0.3695	0.3839	0.4712
1	0.3602	0.3709	0.4134
1.5	0.3368	0.3489	0.3858

fc = 0.1

bf\uf	0.5	1	1.5
0.5	0.6411	0.9659	0.9682
1	0.4659	0.5812	0.9279
1.5	0.4783	0.5849	0.6578

Table S7

mRNAa FF for the full model

fc = 1.0

bf\uf	0.5	1	1.5
0.5	0.9034	1.2132	0.7552
1	0.9321	1.0207	0.9178
1.5	0.9288	1.0542	1.1753

fc = 0.3

bf\uf	0.5	1	1.5
0.5	1.0220	0.9499	1.1603
1	1.1160	1.0149	1.1365
1.5	1.0598	0.9278	1.0977

fc = 0.1

bf\uf	0.5	1	1.5
0.5	0.9827	1.1006	0.8970
1	0.8986	0.8830	1.3428
1.5	1.0886	1.2614	1.0644

6. Supplementary Figures

Figure S1: Experimental data from fluorescence readings and deterministic time-series plots

Experimental data from fluorescence readings in [2] and deterministic time-series plots with ordinary differential equation simulations with the reduced and the full models.

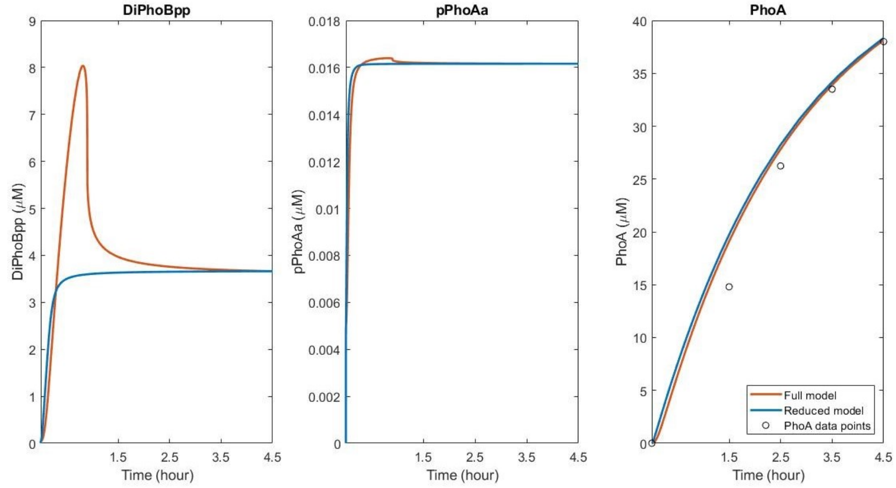

Figure S2: Bar graphs of pPhoAa Fano factors

Bar graphs of pPhoAa Fano factors obtained by varying uf and bf with different external P_i concentrations, fc $\in \{0.1, 0.3, 1.0\}$, in both full and reduced models. *The range of the vertical axis is different in each plot, which should highlight the difference between individual cases.*

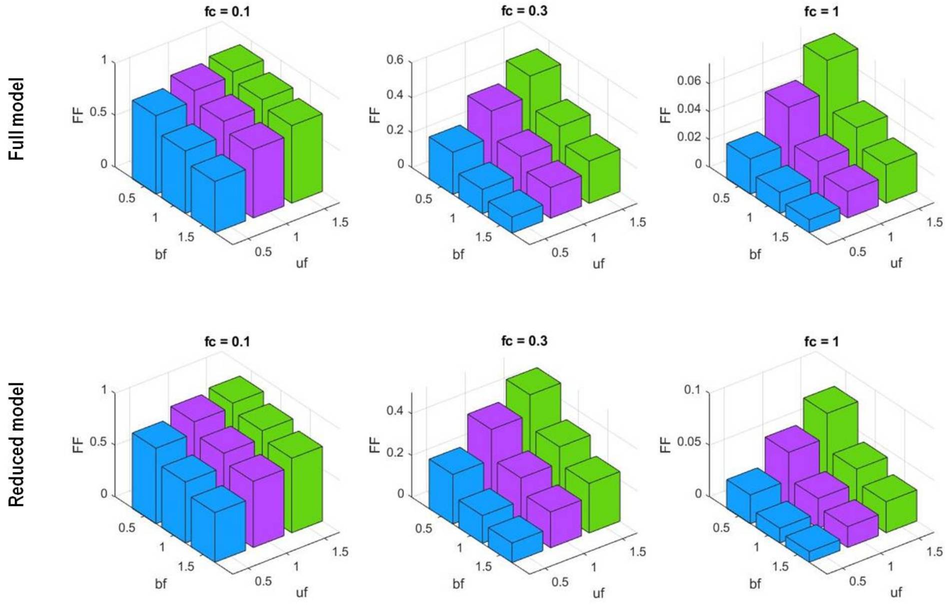

Figure S3: Bar graphs of CV and FF values of mRNAa

Bar graphs of CV and FF values of mRNAa obtained by varying uf and bf with different external P_i concentrations, fc $\in \{0.1, 0.3, 1.0\}$, in the **full model**. *The range of the vertical axis is different in each plot, which should highlight the difference between individual cases.* The lowest intrinsic noise levels correspond to low unbinding factors and high binding factors for the pPhoAa promoter.

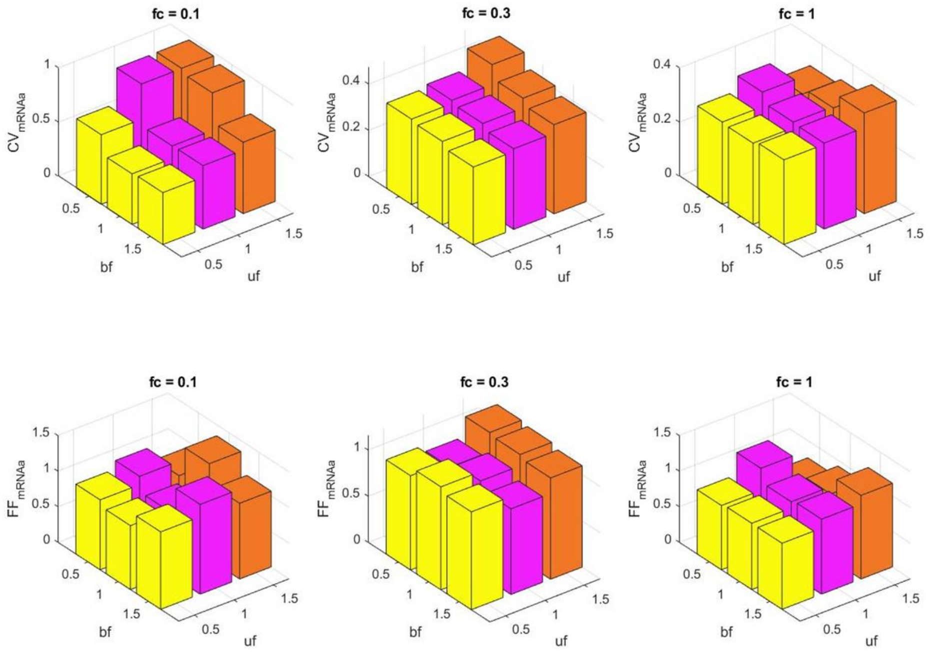

Figure S4: Bar graphs of mean mRNAa levels at equilibrium

Bar graphs of mean mRNAa levels (μ_{mRNAa}) at equilibrium obtained by varying uf and bf with different external P_i concentrations, fc $\in \{0.1, 0.3, 1.0\}$, in the **full model**. *The range of the vertical axis is different in each plot, which should highlight the difference between individual cases.*

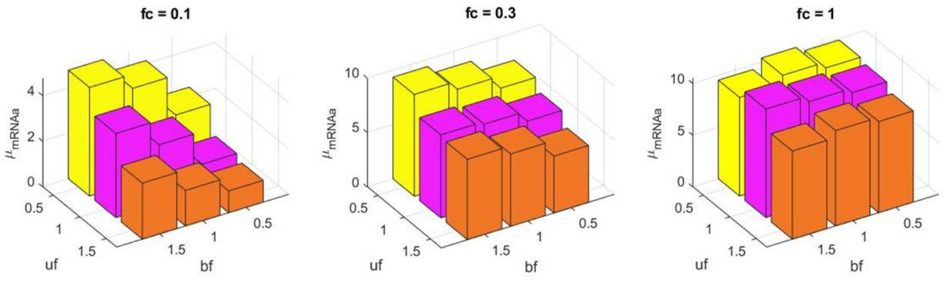

Figure S5: Heatmaps of pPhoAa Fano factors for pPhoAa

Heatmaps of pPhoAa Fano factors for pPhoAa obtained by varying uf, bf $\in \{0.01, 0.1, 1, 10, 100\}$ with different external P_i concentrations with fc $\in \{0.1, 0.3, 1.0\}$ in the reduced model.

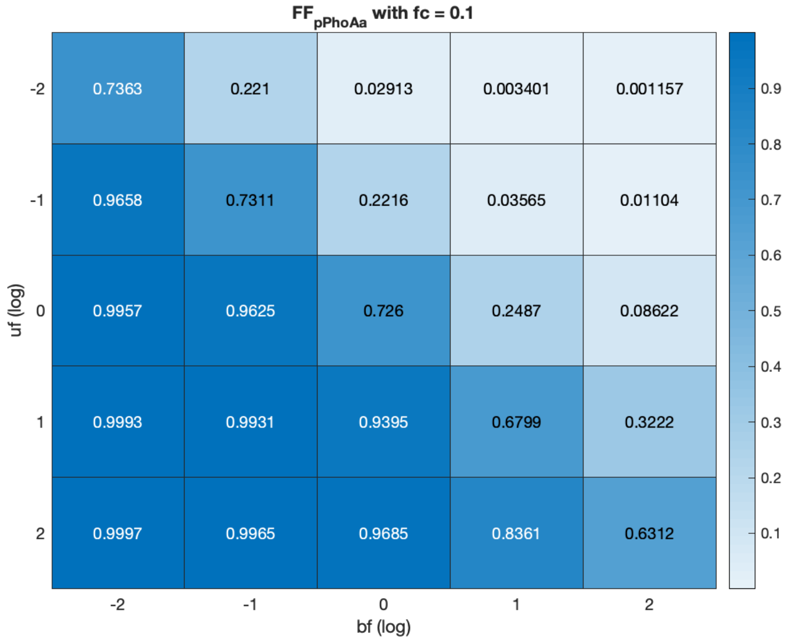

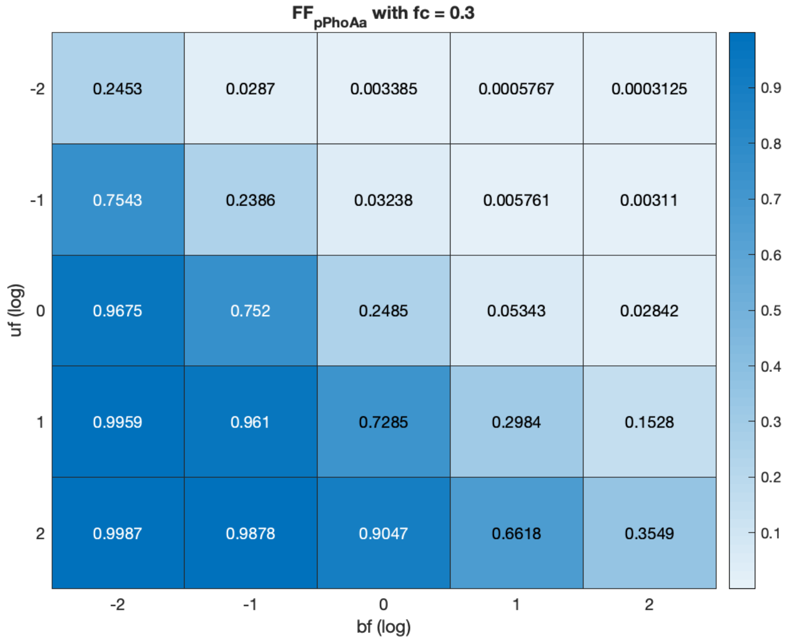

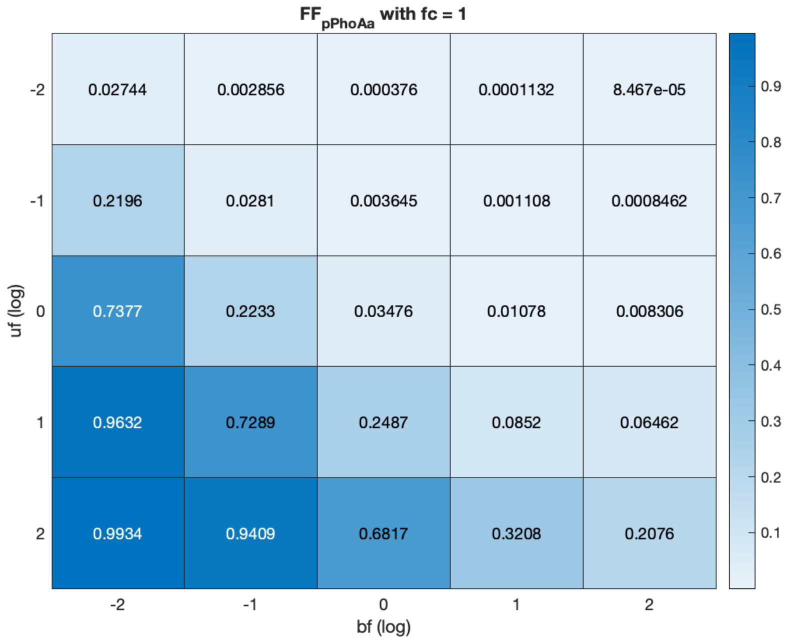

FF pPhoAa with fc = 1
uf (log)
bf (log)
-2 -1 0 1 2
0.02744 0.002856 0.000376 0.0001132 8.467e-05
0.2196 0.0281 0.003645 0.001108 0.0008462
0.7377 0.2233 0.03476 0.01078 0.008306
0.9632 0.7289 0.2487 0.0852 0.06462
0.9934 0.9409 0.6817 0.3208 0.2076
0.9 0.8 0.7 0.6 0.5 0.4 0.3 0.2 0.1

Figure S6: Stochastic Simulation Time Series Samples

$$\mathrm{fc} = 0.1, \mathrm{bf} = 0.01, \mathrm{uf} = 0.01$$

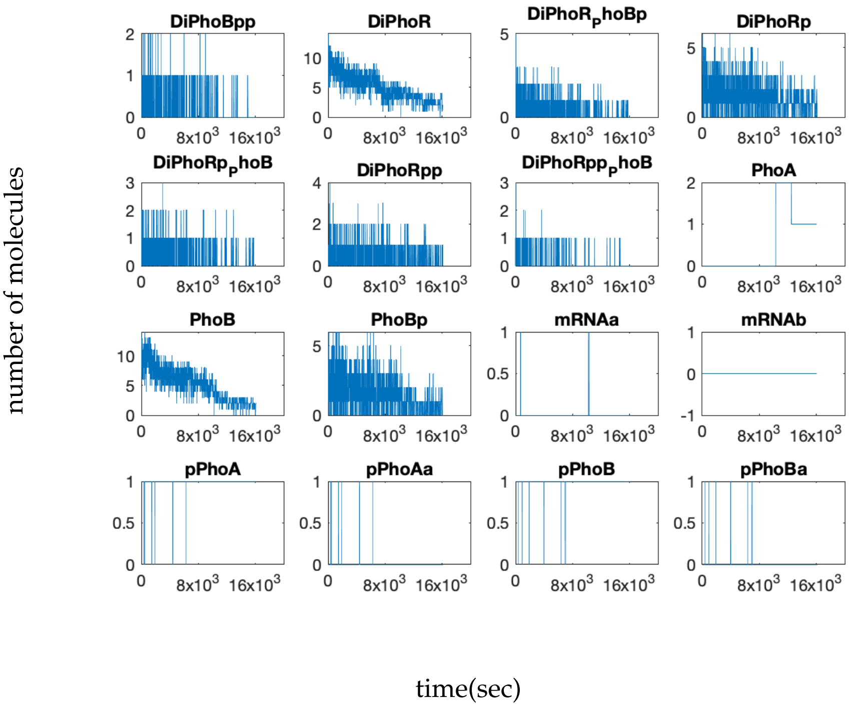

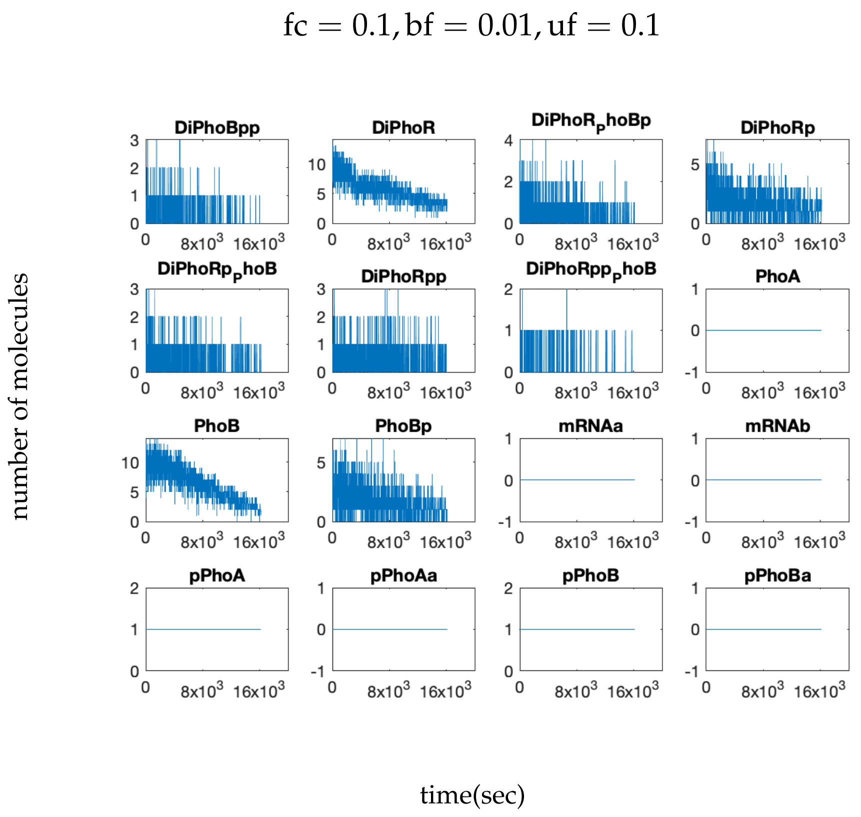
fc = 0.1, bf = 0.01, uf = 0.1
number of molecules
DiPhoBpp
DiPhoR
DiPhoR_PhoBp
DiPhoRp
DiPhoRp_PhoB
DiPhoRpp
DiPhoRpp_PhoB
PhoA
PhoB
PhoBp
mRNAa
mRNAb
pPhoA
pPhoAa
pPhoB
pPhoBa
0
8x10^3
16x10^3
time(sec)

$$fc = 0.1, bf = 0.01, uf = 1$$

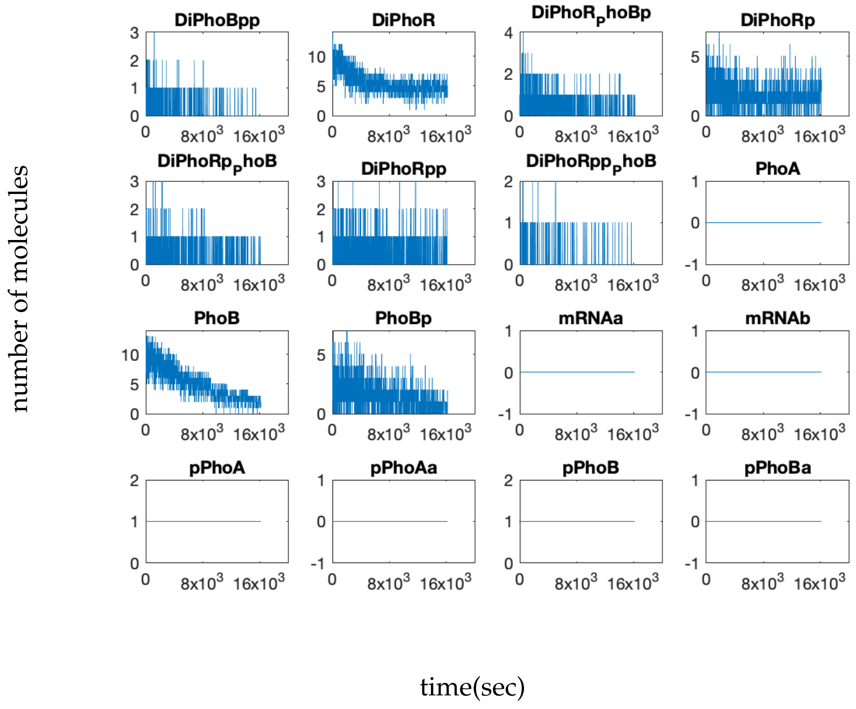

$$\mathrm{fc} = 0.1, \mathrm{bf} = 0.01, \mathrm{uf} = 10$$

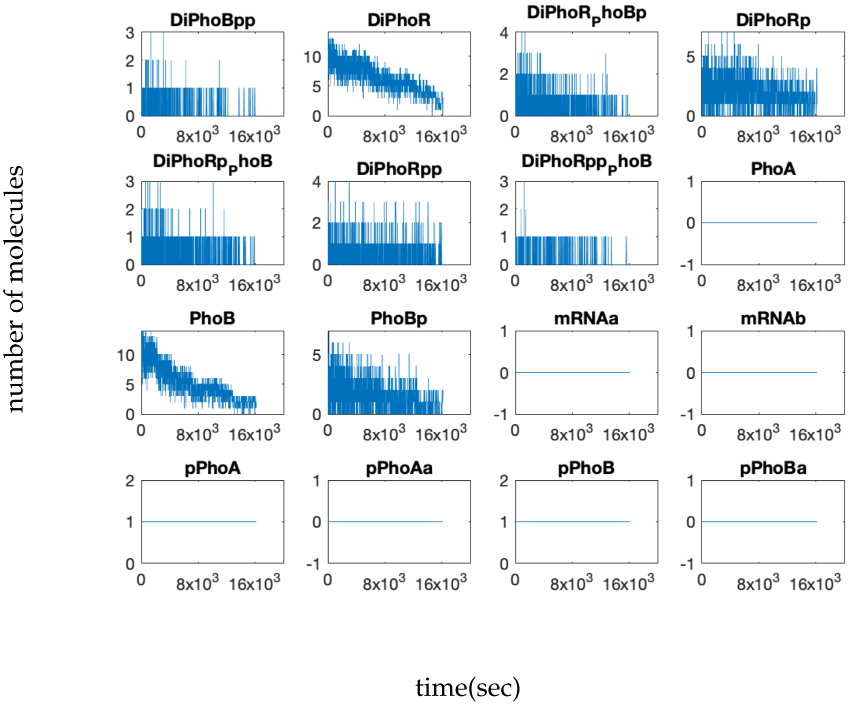

$$\mathrm{fc} = 0.1, \mathrm{bf} = 0.01, \mathrm{uf} = 100$$

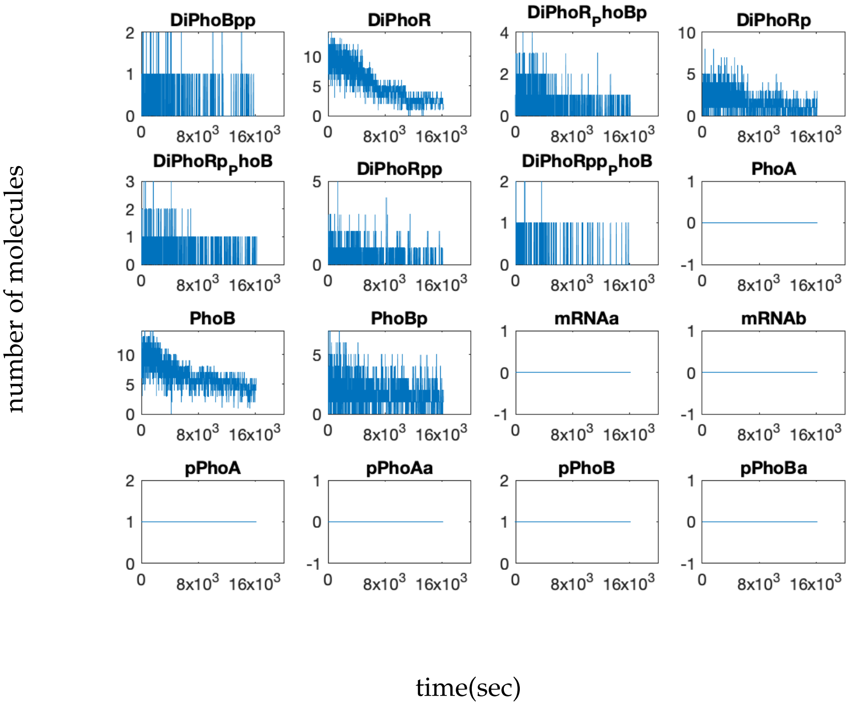

$$\mathrm{fc} = 0.1, \mathrm{bf} = 0.1, \mathrm{uf} = 0.01$$

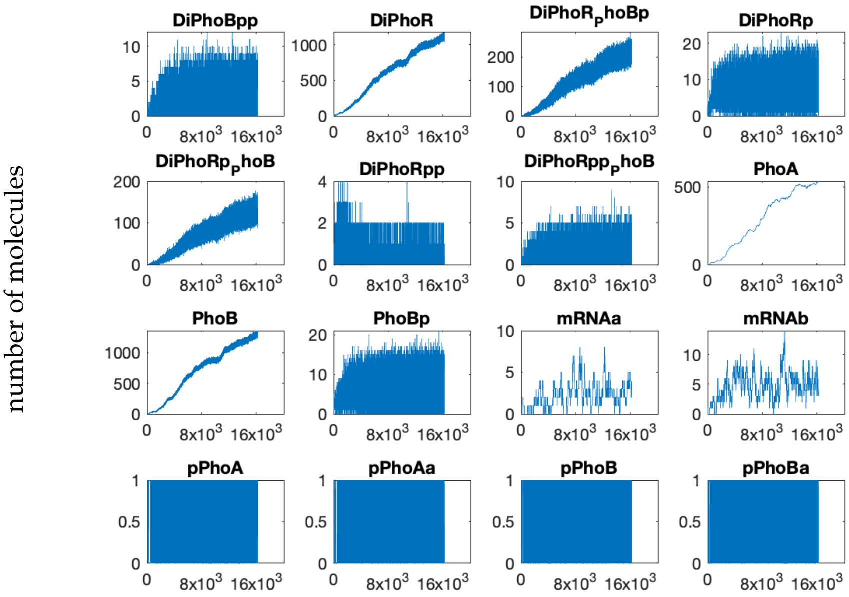

time(sec)

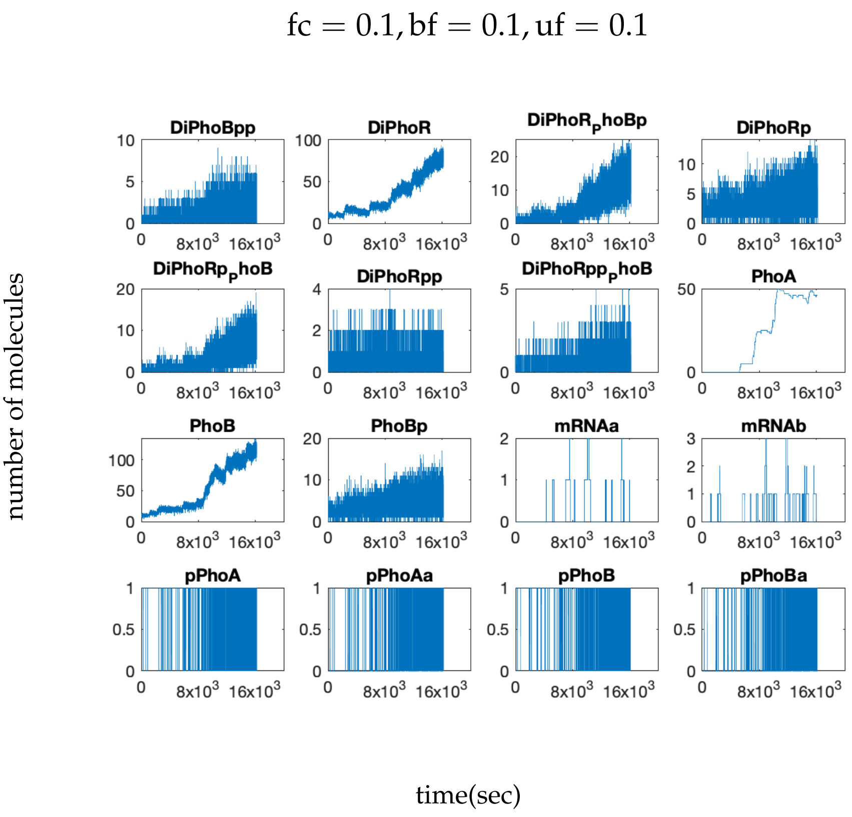
fc = 0.1, bf = 0.1, uf = 0.1
DiPhoBpp
DiPhoR
DiPhoR_PhoBp
DiPhoRp
DiPhoRp_PhoB
DiPhoRpp
DiPhoRpp_PhoB
PhoA
PhoB
PhoBp
mRNAa
mRNAb
pPhoA
pPhoAa
pPhoB
pPhoBa
number of molecules
time(sec)
8x10^3
16x10^3

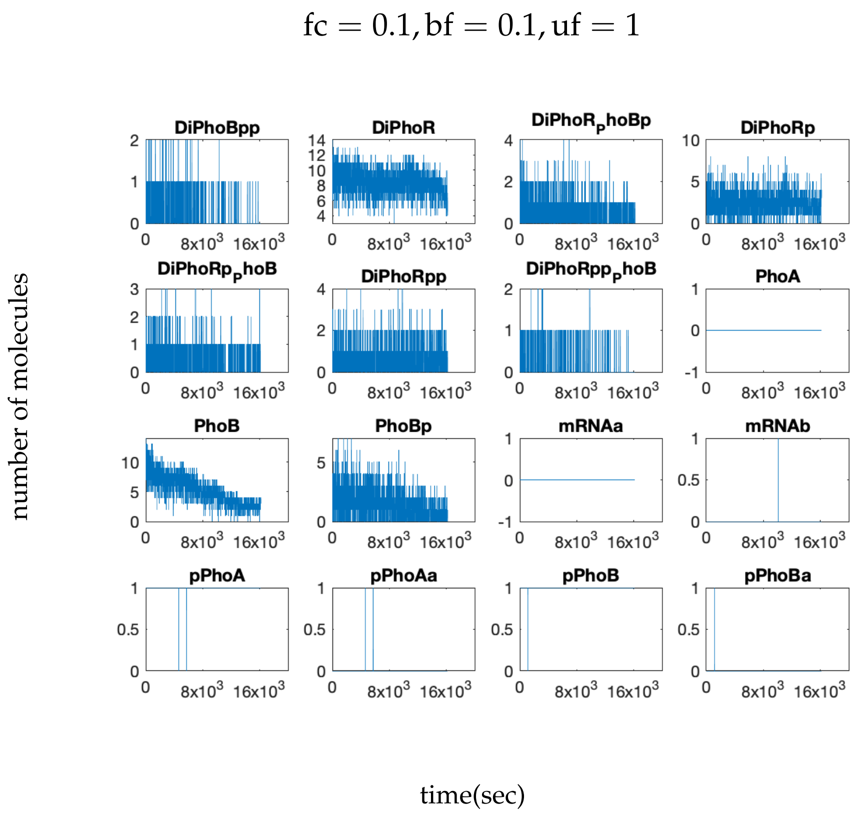
fc = 0.1, bf = 0.1, uf = 1
DiPhoBpp
DiPhoR
DiPhoR$_P$hoBp
DiPhoRp
DiPhoRp$_P$hoB
DiPhoRpp
DiPhoRpp$_P$hoB
PhoA
PhoB
PhoBp
mRNAa
mRNAb
pPhoA
pPhoAa
pPhoB
pPhoBa
8x10^3
16x10^3
number of molecules
time(sec)

$$\mathrm{fc} = 0.1, \mathrm{bf} = 0.1, \mathrm{uf} = 10$$

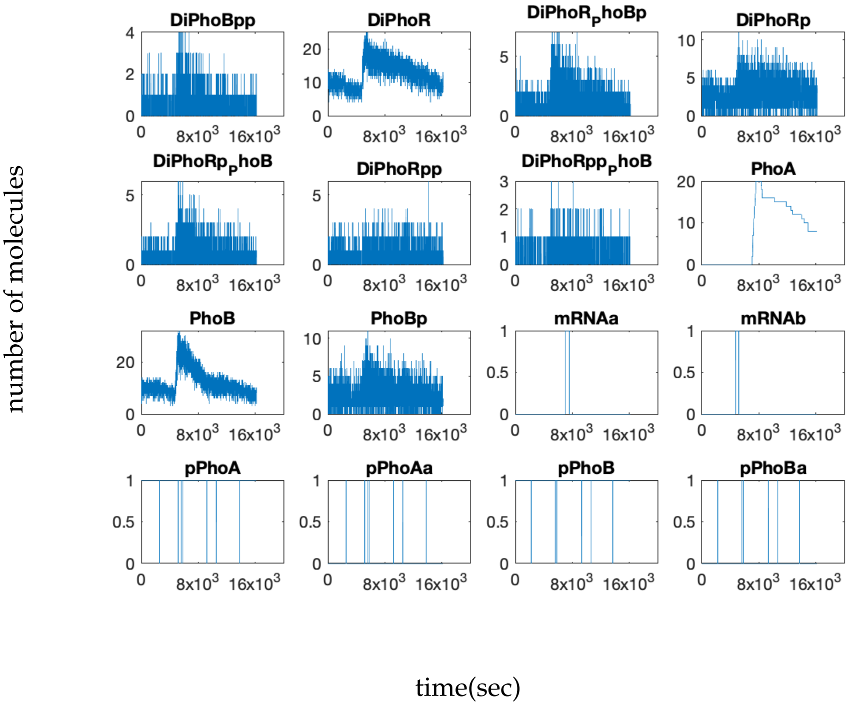

$$fc = 0.1, bf = 0.1, uf = 100$$

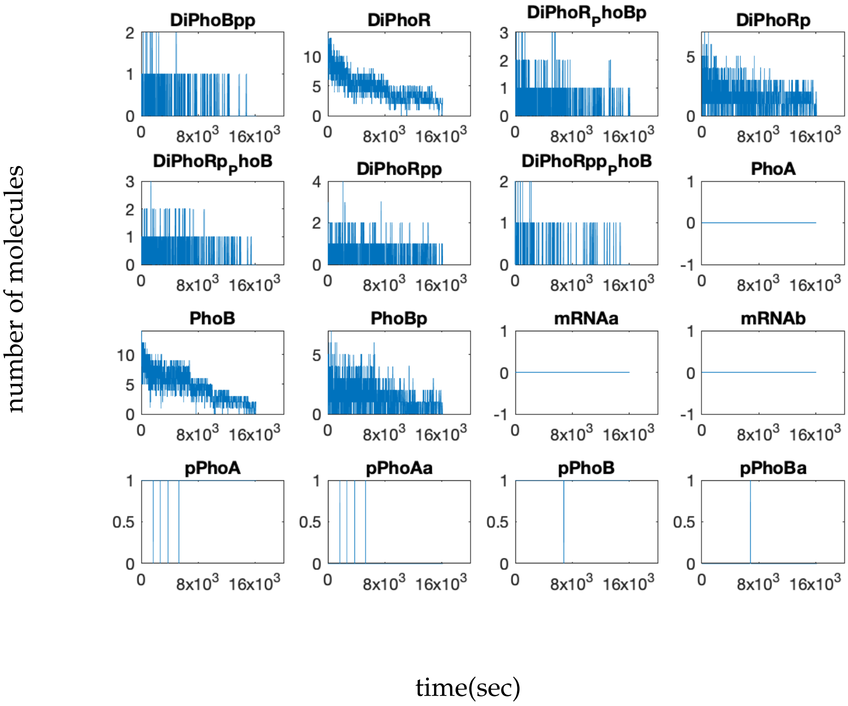

$$\mathrm{fc} = 0.1, \mathrm{bf} = 1, \mathrm{uf} = 0.01$$

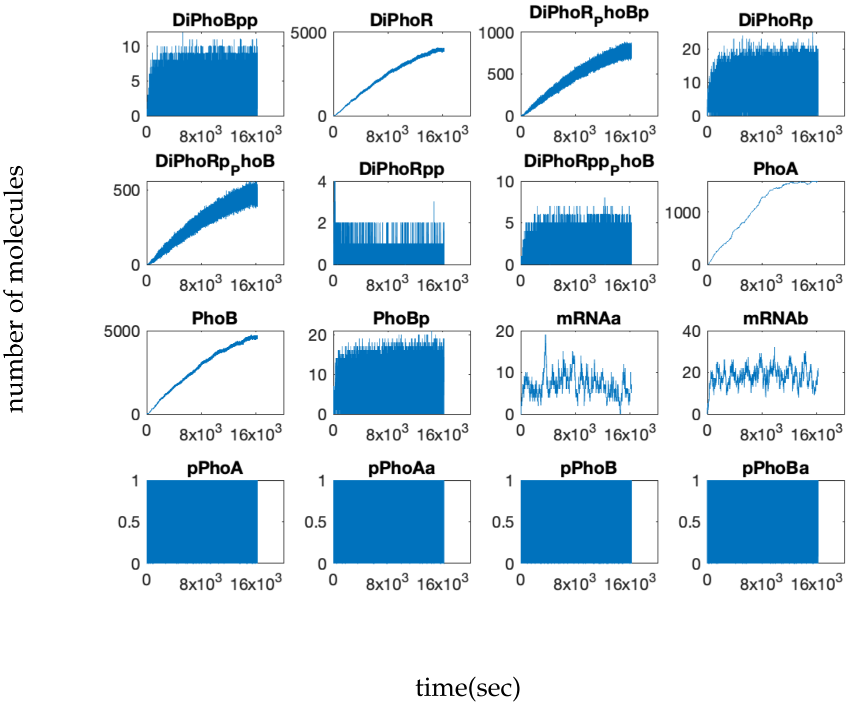

$$\mathrm{fc} = 0.1, \mathrm{bf} = 1, \mathrm{uf} = 0.1$$

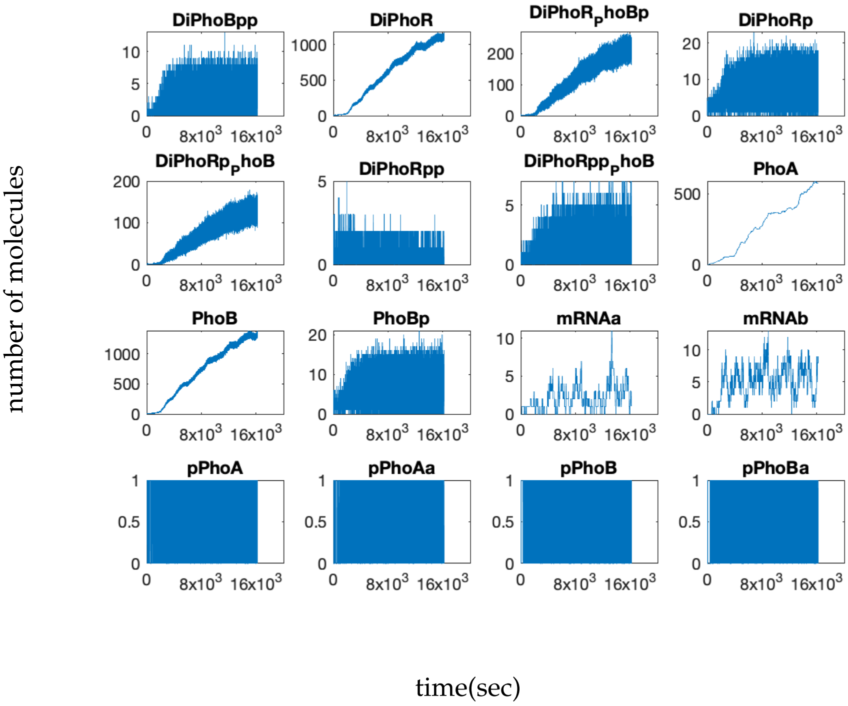

$$\mathrm{fc} = 0.1, \mathrm{bf} = 1, \mathrm{uf} = 1$$

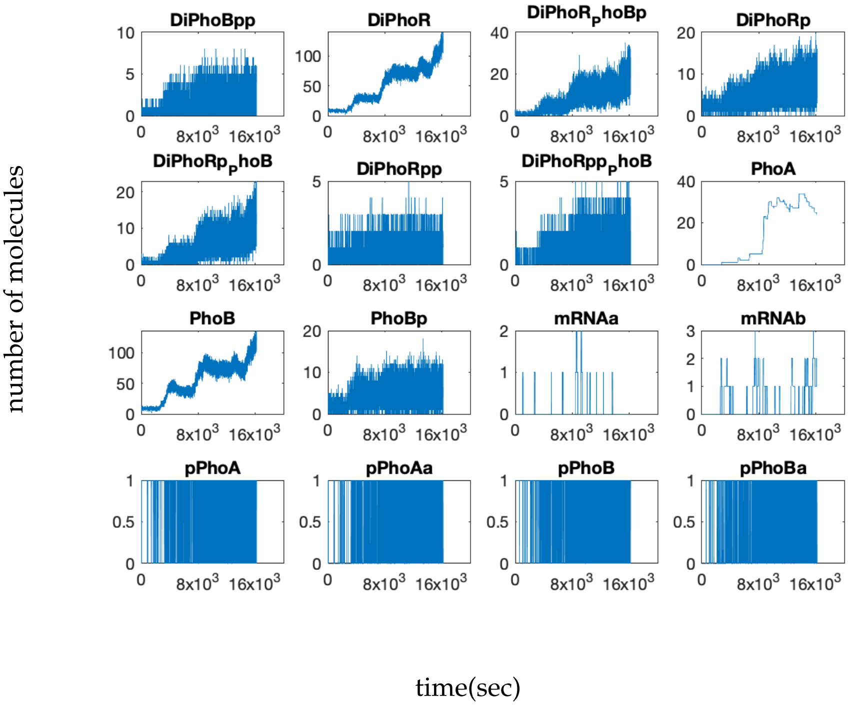

$$fc = 0.1, bf = 1, uf = 10$$

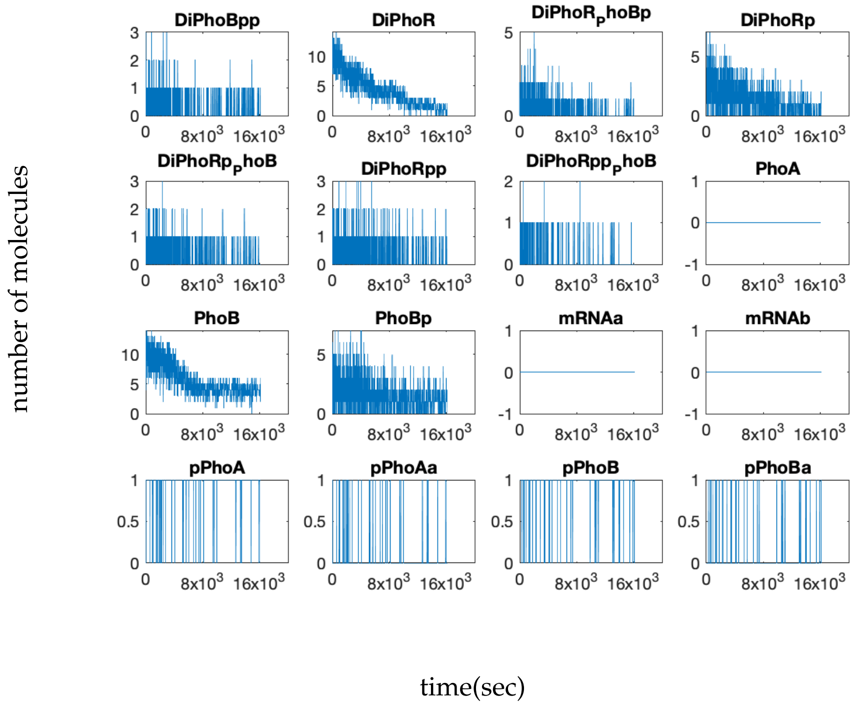

$$\mathrm{fc} = 0.1, \mathrm{bf} = 1, \mathrm{uf} = 100$$

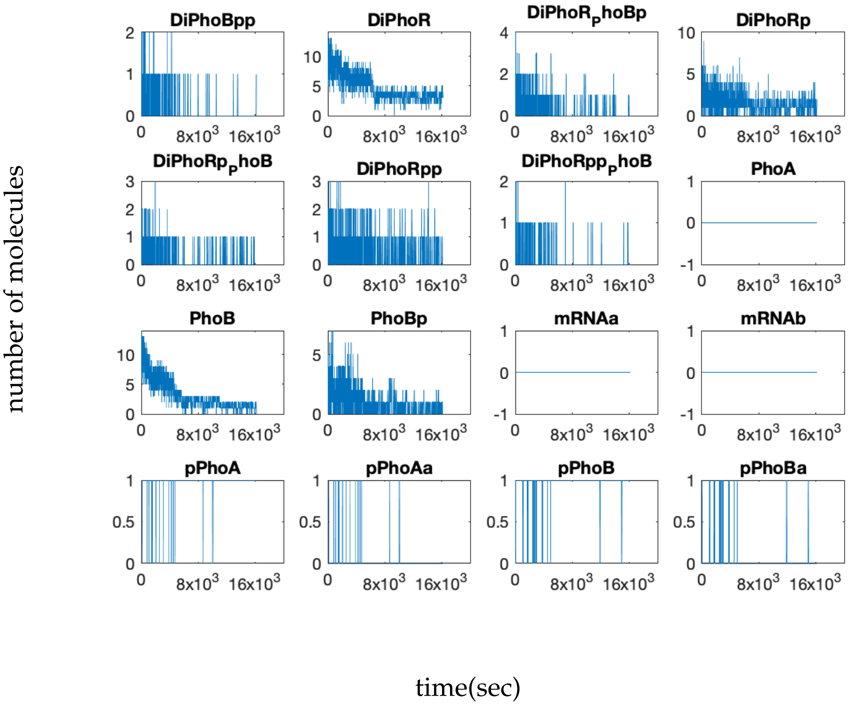

$$\mathrm{fc} = 0.1, \mathrm{bf} = 10, \mathrm{uf} = 0.01$$

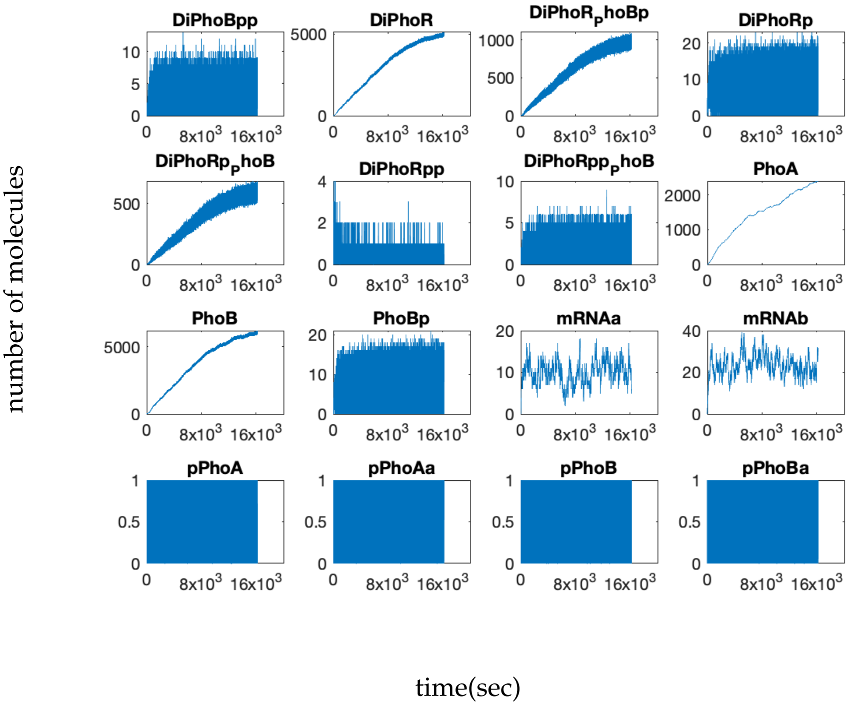

$$\mathrm{fc} = 0.1, \mathrm{bf} = 10, \mathrm{uf} = 0.1$$

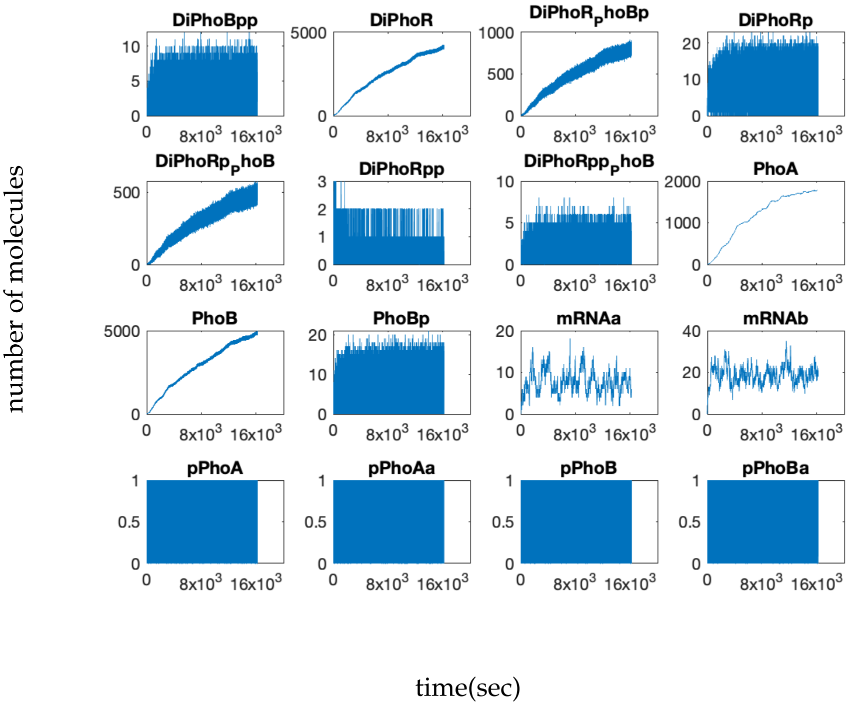

$$\mathrm{fc} = 0.1, \mathrm{bf} = 10, \mathrm{uf} = 1$$

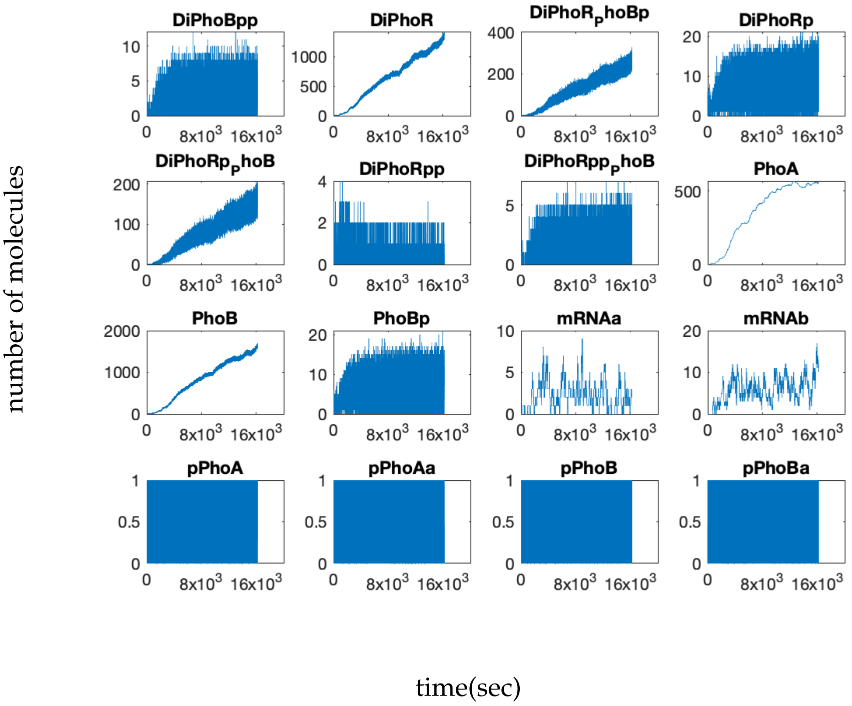

$\text{fc} = 0.1, \text{bf} = 10, \text{uf} = 10$

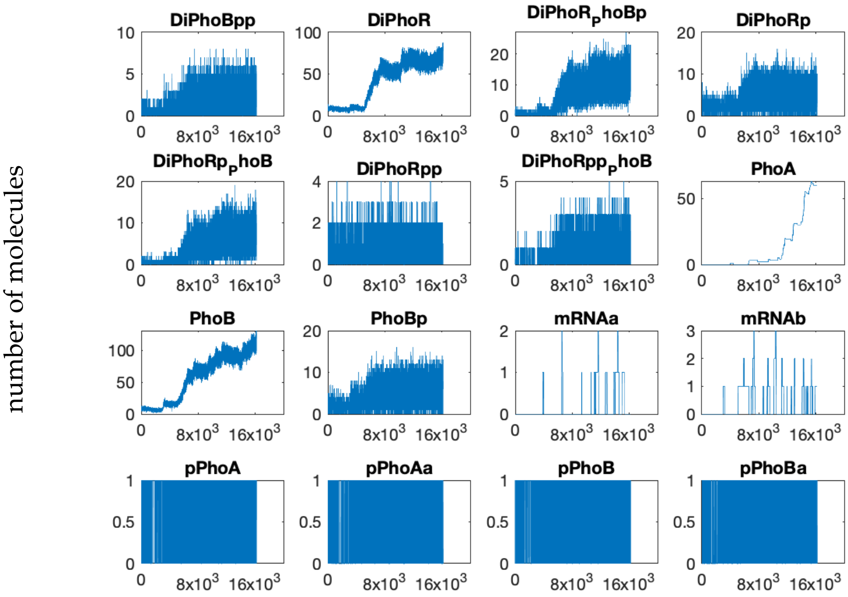

time(sec)

$\mathrm{fc} = 0.1, \mathrm{bf} = 10, \mathrm{uf} = 100$

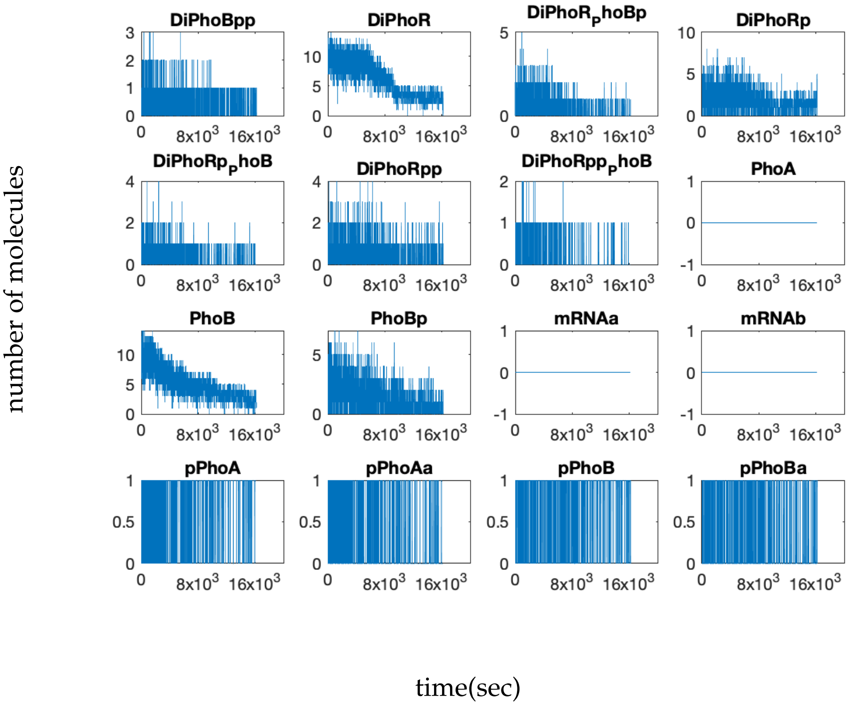

$$\mathrm{fc} = 0.1, \mathrm{bf} = 100, \mathrm{uf} = 0.01$$

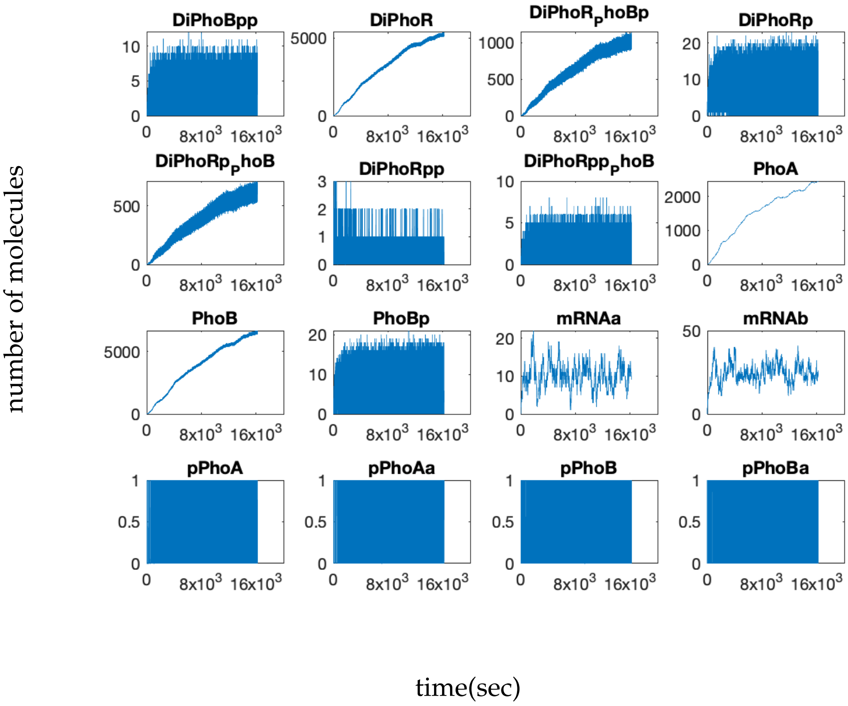

$$\mathrm{fc} = 0.1, \mathrm{bf} = 100, \mathrm{uf} = 0.1$$

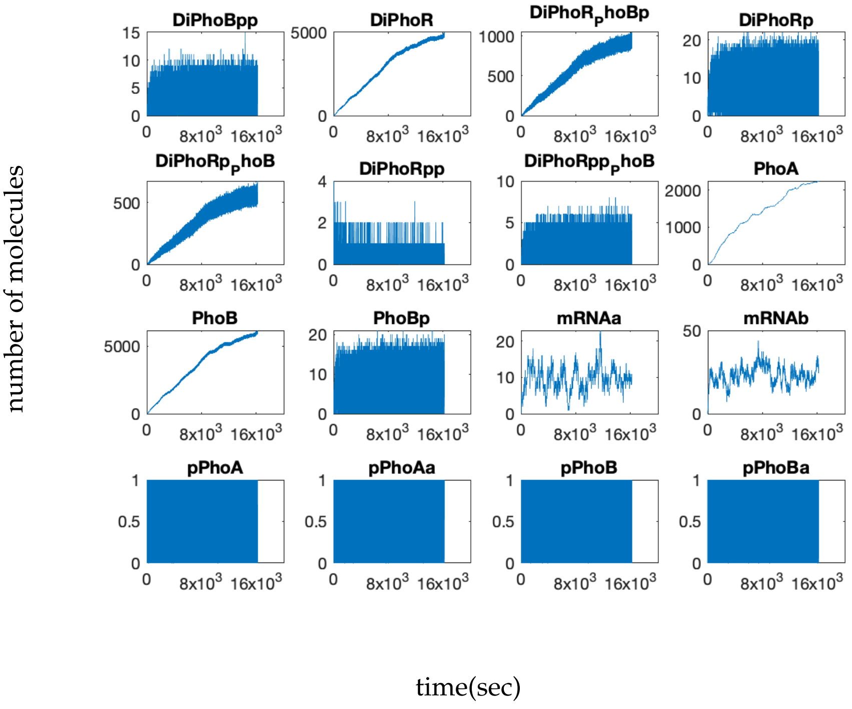

$$\mathrm{fc} = 0.1, \mathrm{bf} = 100, \mathrm{uf} = 1$$

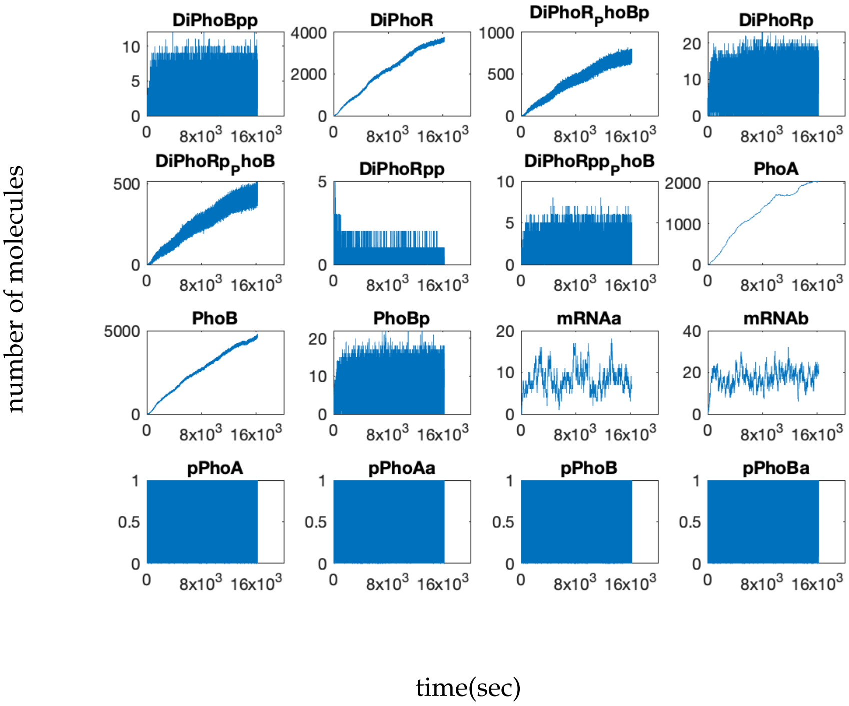

$$\mathrm{fc} = 0.1, \mathrm{bf} = 100, \mathrm{uf} = 10$$

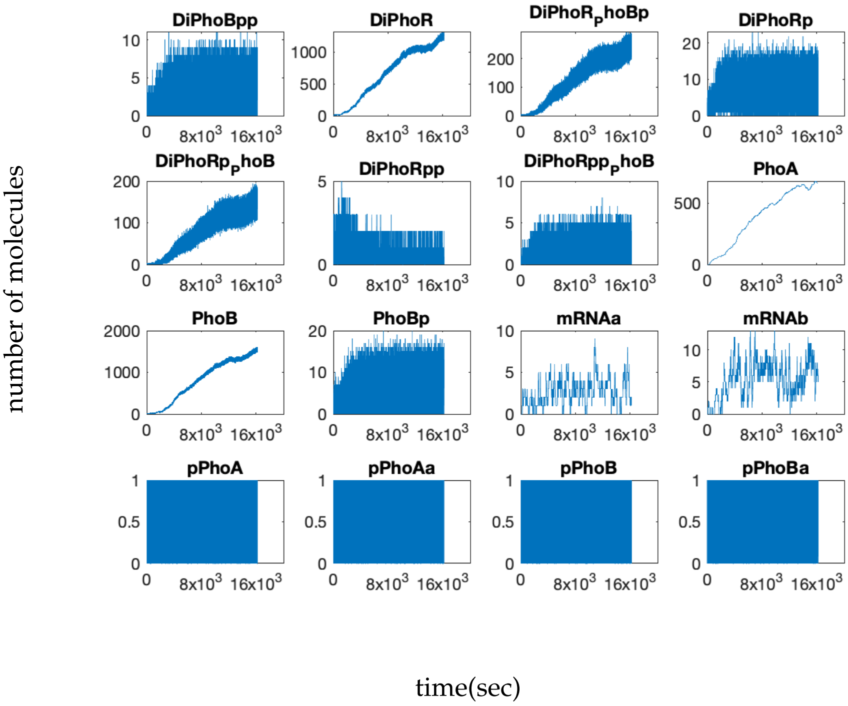

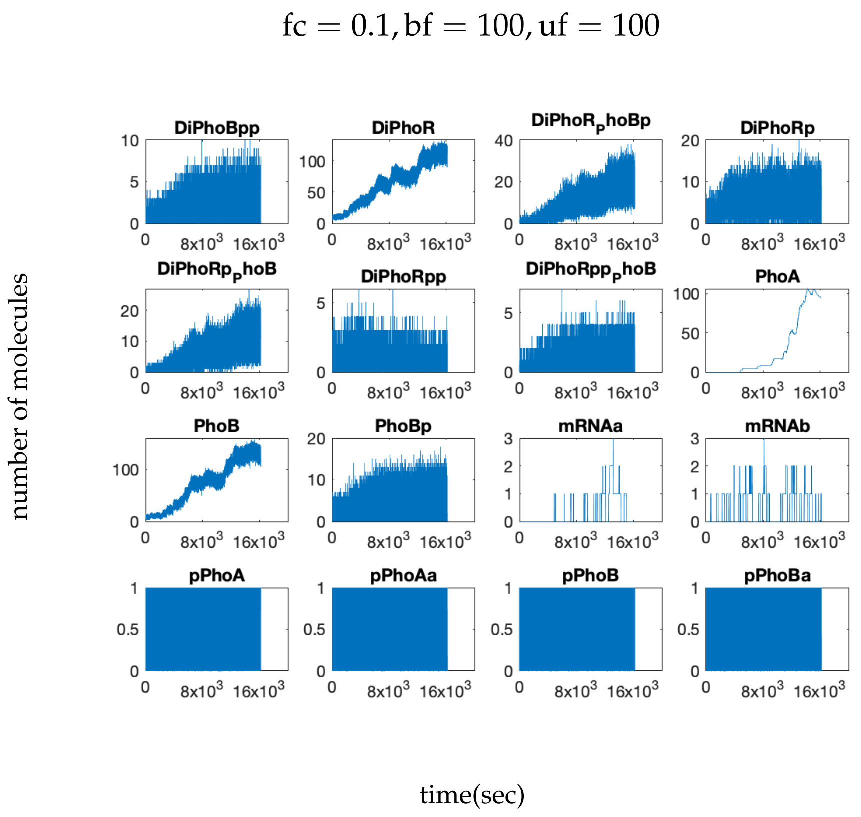
fc = 0.1, bf = 100, uf = 100
number of molecules
time(sec)
DiPhoBpp
DiPhoR
DiPhoR_PhoBp
DiPhoRp
DiPhoRp_PhoB
DiPhoRpp
DiPhoRpp_PhoB
PhoA
PhoB
PhoBp
mRNAa
mRNAb
pPhoA
pPhoAa
pPhoB
pPhoBa

$$\mathrm{fc} = 0.3, \mathrm{bf} = 0.01, \mathrm{uf} = 0.01$$

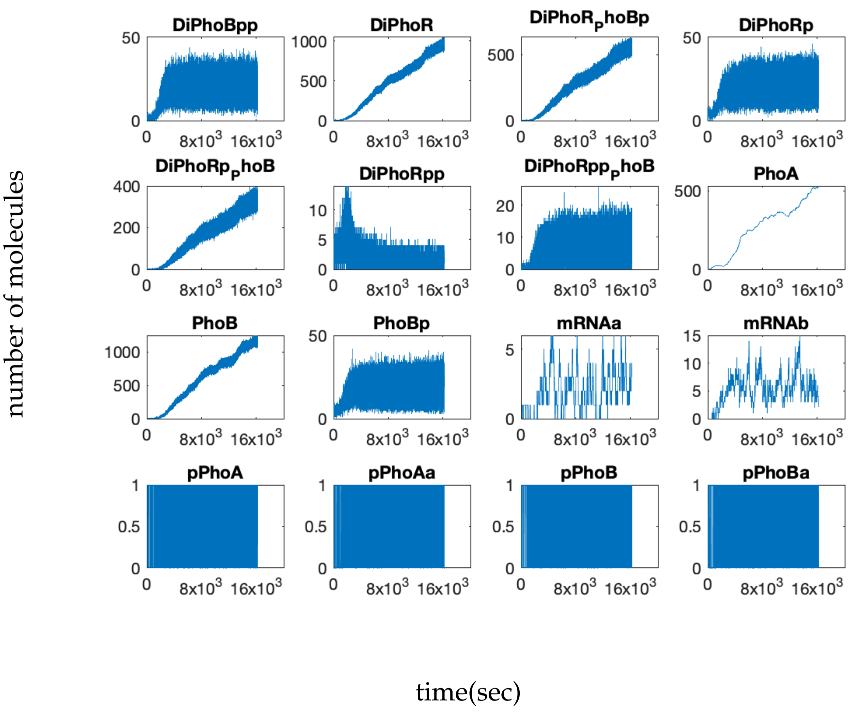

$$fc = 0.3, bf = 0.01, uf = 0.1$$

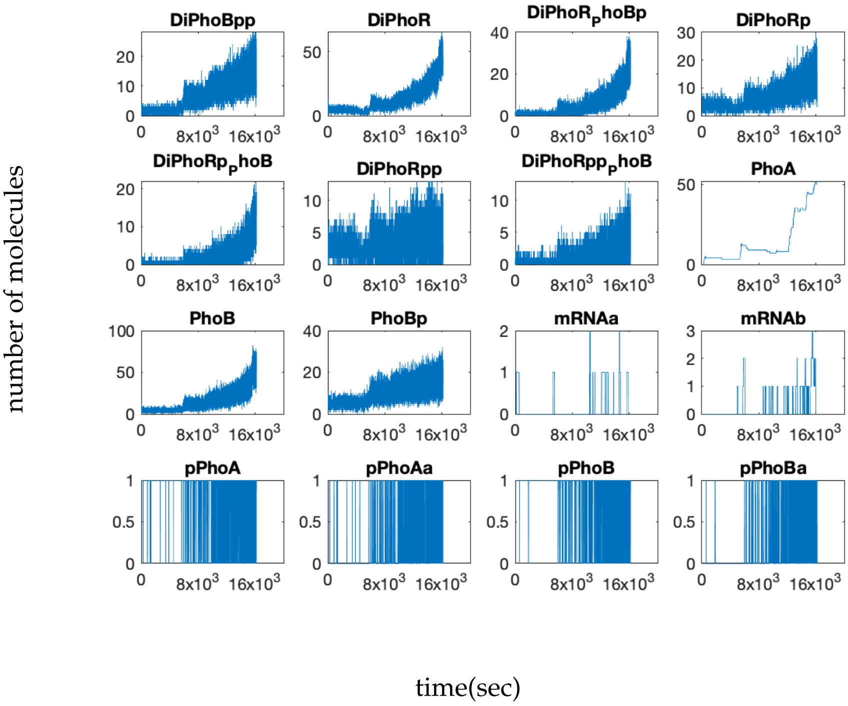

$$fc = 0.3, bf = 0.01, uf = 1$$

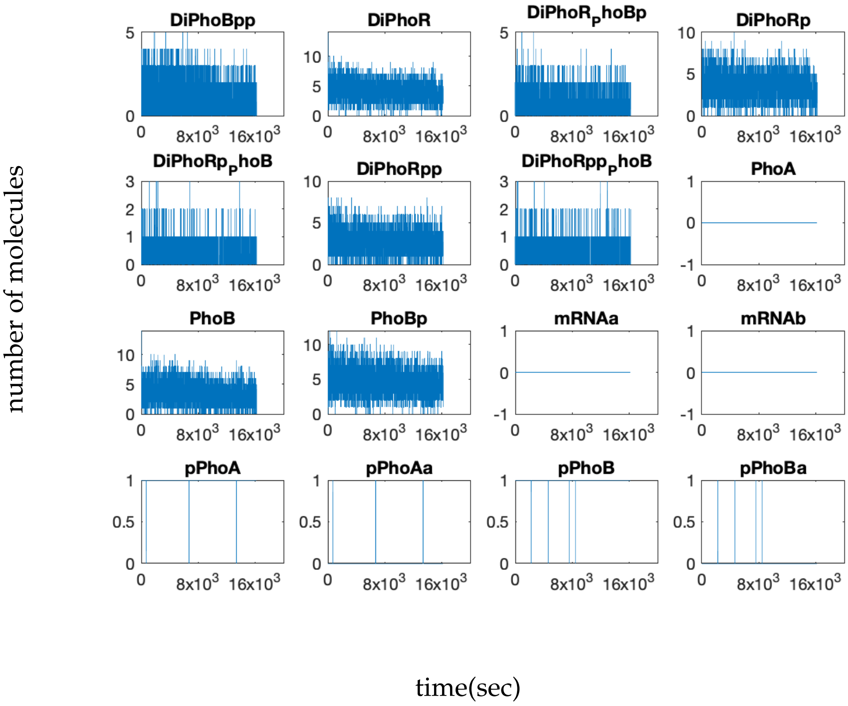

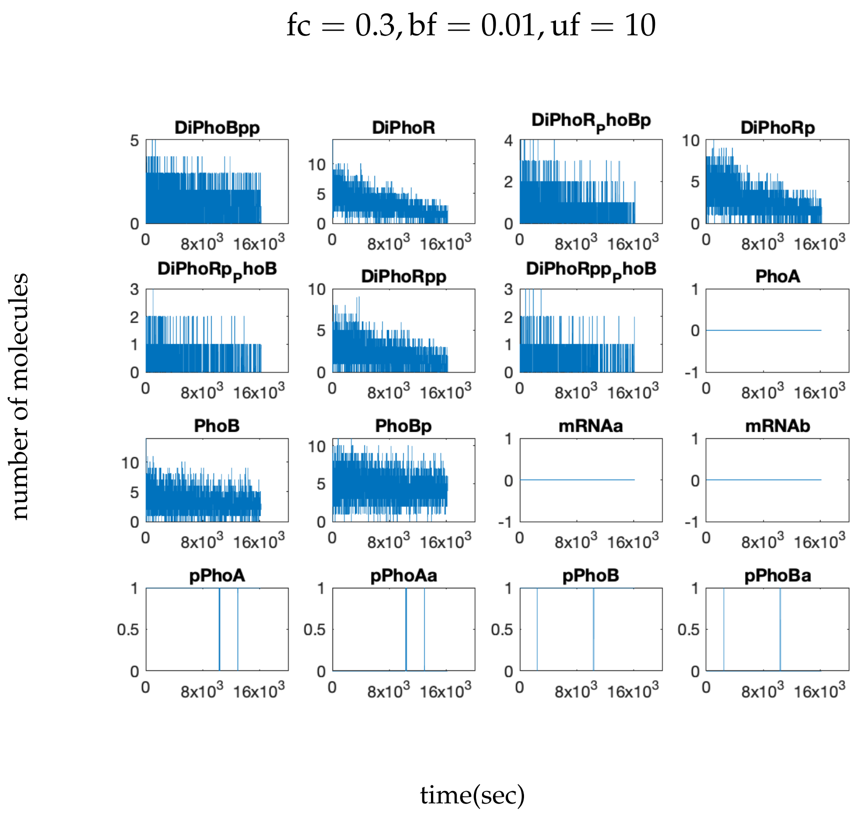
fc = 0.3, bf = 0.01, uf = 10
number of molecules
DiPhoBpp
DiPhoR
DiPhoR$_P$hoBp
DiPhoRp
DiPhoRp$_P$hoB
DiPhoRpp
DiPhoRpp$_P$hoB
PhoA
PhoB
PhoBp
mRNAa
mRNAb
pPhoA
pPhoAa
pPhoB
pPhoBa
0
8x10^3
16x10^3
time(sec)

$$\mathrm{fc} = 0.3, \mathrm{bf} = 0.01, \mathrm{uf} = 100$$

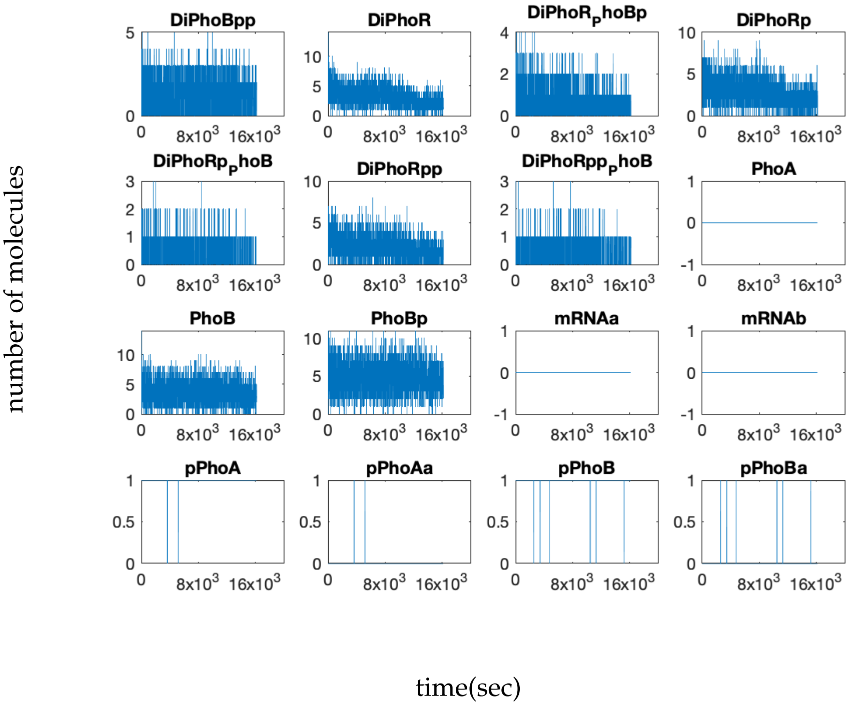

$fc = 0.3, bf = 0.1, uf = 0.01$

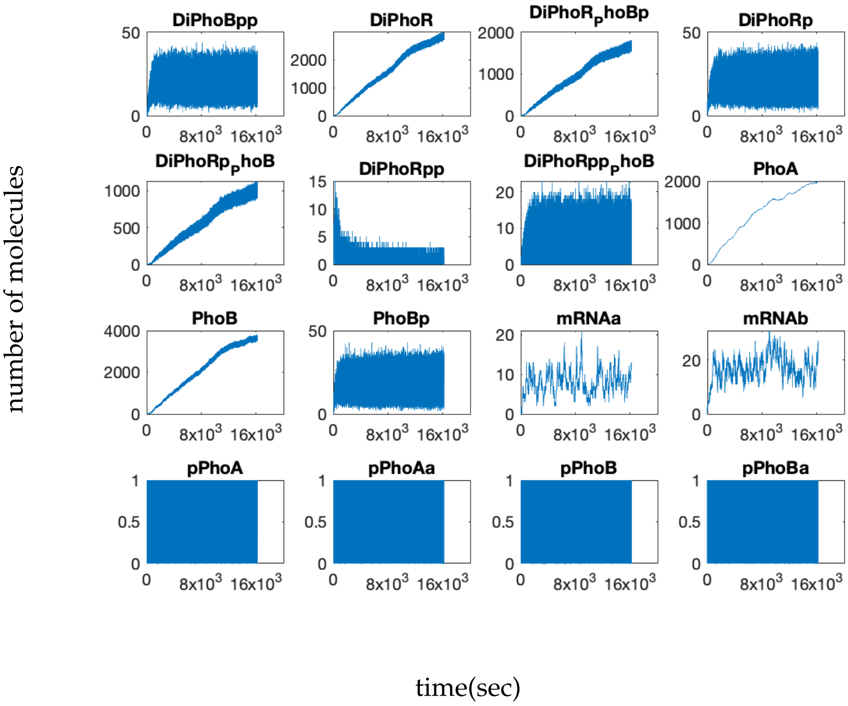

$$fc = 0.3, bf = 0.1, uf = 0.1$$

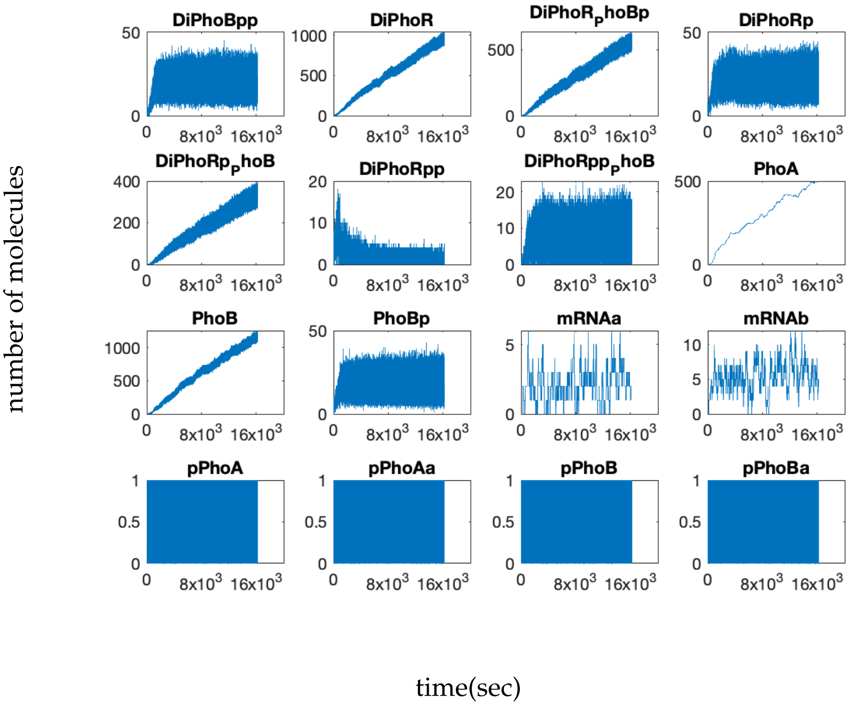

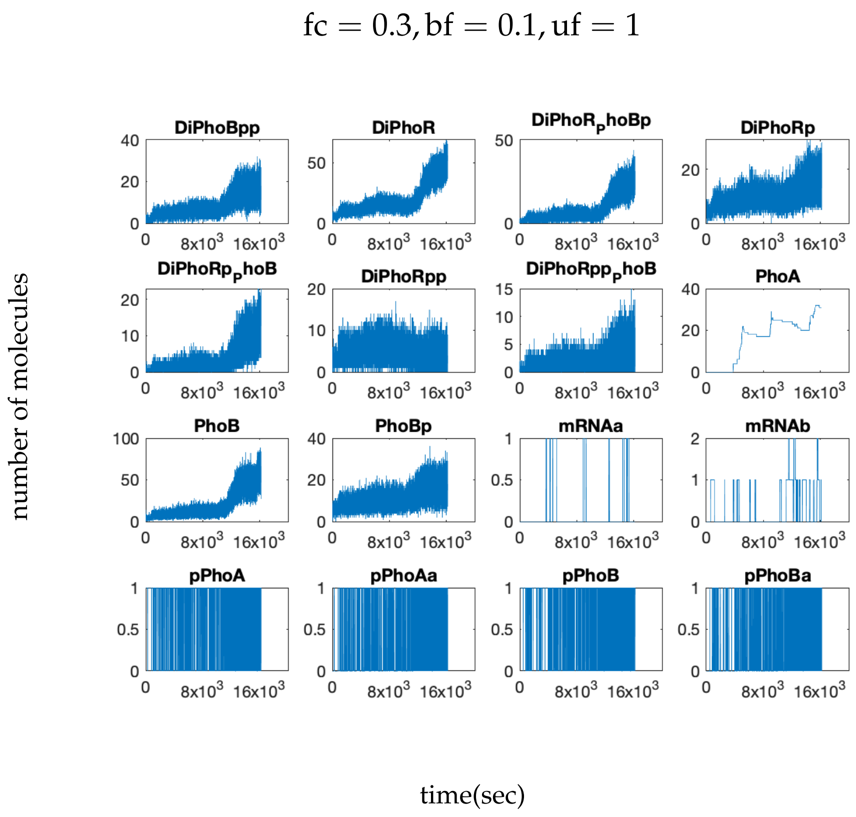

fc = 0.3, bf = 0.1, uf = 1
DiPhoBpp
DiPhoR
DiPhoR_PhoBp
DiPhoRp
DiPhoRp_PhoB
DiPhoRpp
DiPhoRpp_PhoB
PhoA
PhoB
PhoBp
mRNAa
mRNAb
pPhoA
pPhoAa
pPhoB
pPhoBa
number of molecules
time(sec)
8x10^3
16x10^3

$$\mathrm{fc} = 0.3, \mathrm{bf} = 0.1, \mathrm{uf} = 10$$

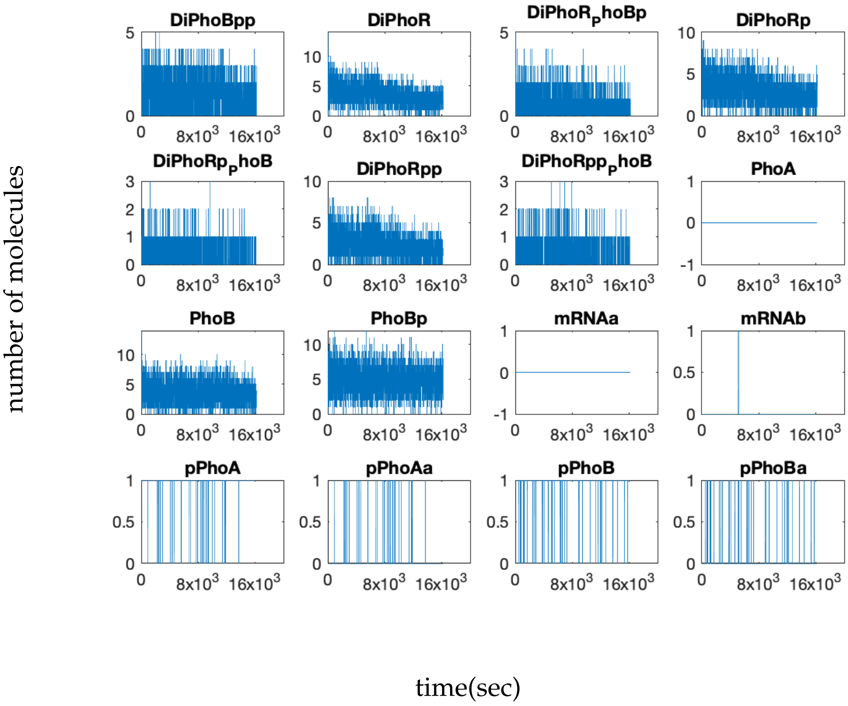

$$fc = 0.3, bf = 0.1, uf = 100$$

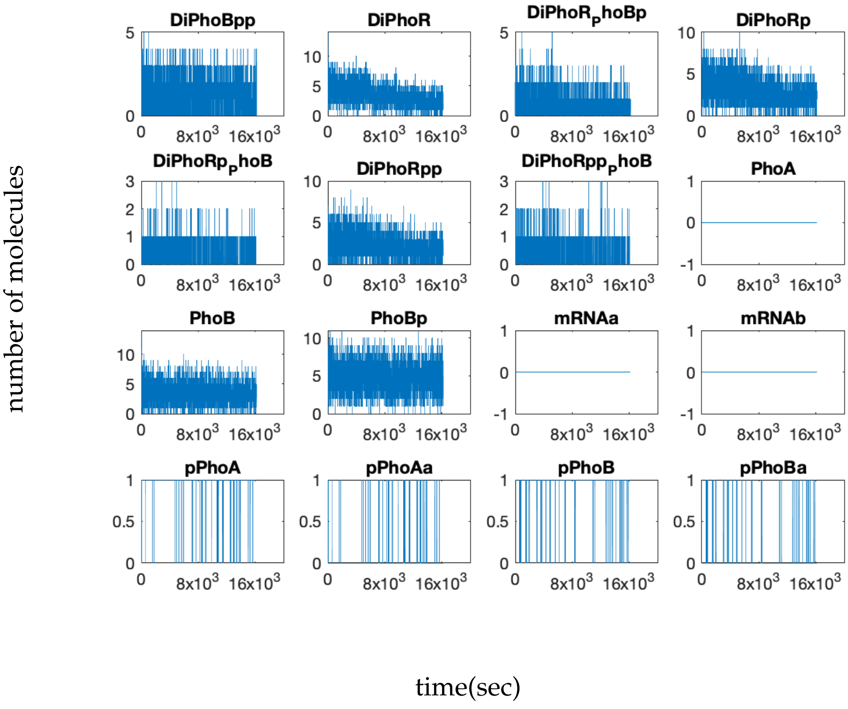

$$fc = 0.3, bf = 1, uf = 0.01$$

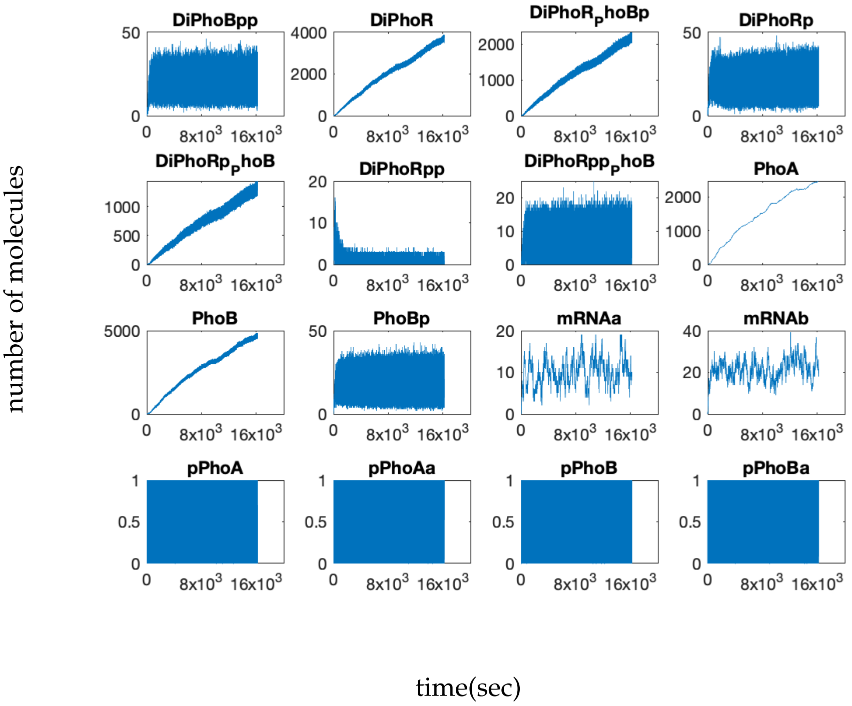

$$\mathrm{fc} = 0.3, \mathrm{bf} = 1, \mathrm{uf} = 0.1$$

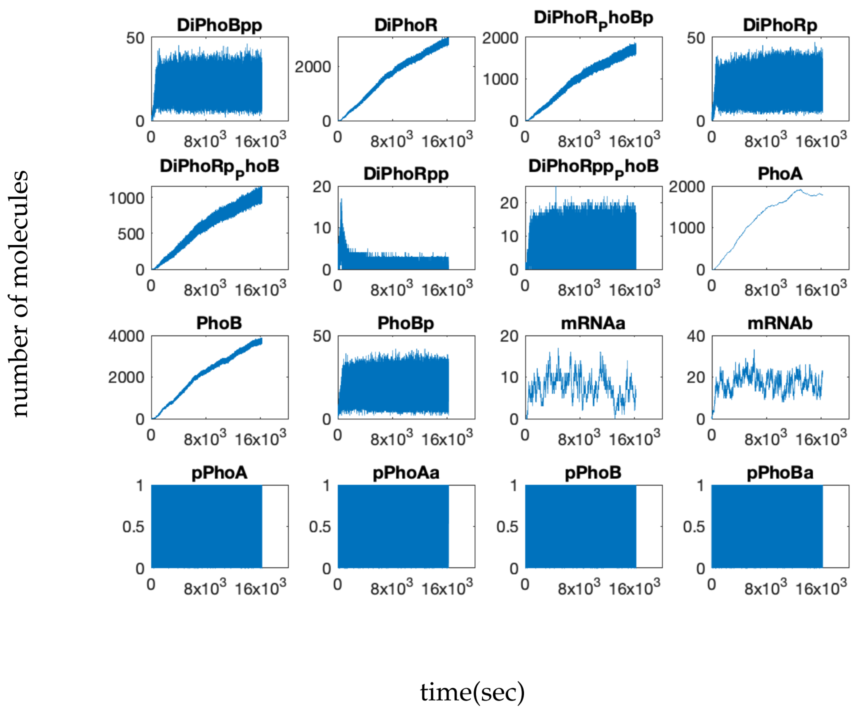

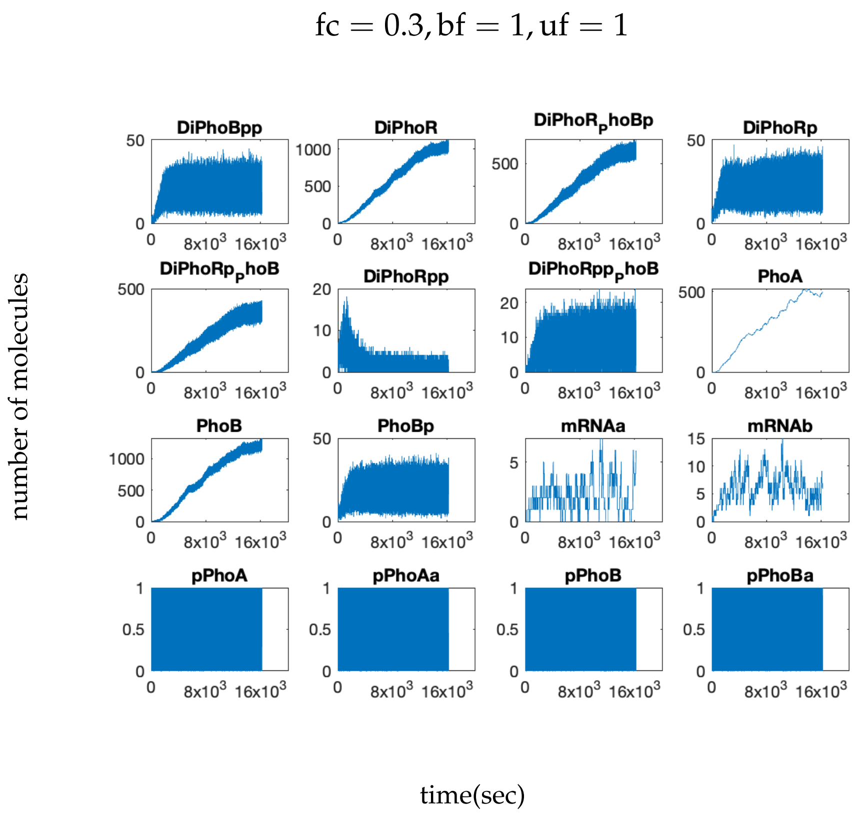
fc = 0.3, bf = 1, uf = 1
DiPhoBpp
DiPhoR
DiPhoR$_P$hoBp
DiPhoRp
DiPhoRp$_P$hoB
DiPhoRpp
DiPhoRpp$_P$hoB
PhoA
PhoB
PhoBp
mRNAa
mRNAb
pPhoA
pPhoAa
pPhoB
pPhoBa
number of molecules
time(sec)

$$\mathrm{fc} = 0.3, \mathrm{bf} = 1, \mathrm{uf} = 10$$

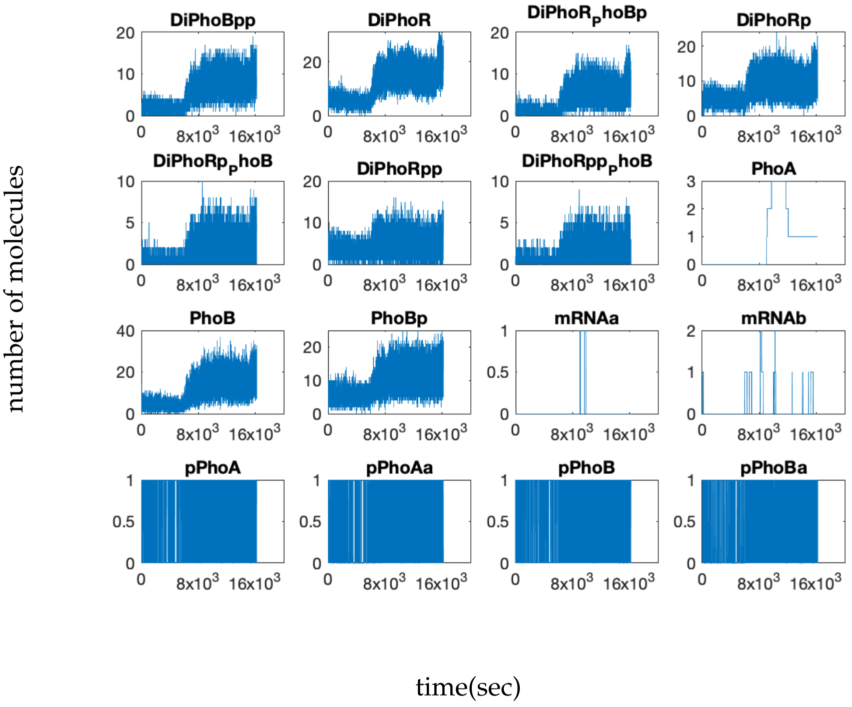

$$\mathrm{fc} = 0.3, \mathrm{bf} = 1, \mathrm{uf} = 100$$

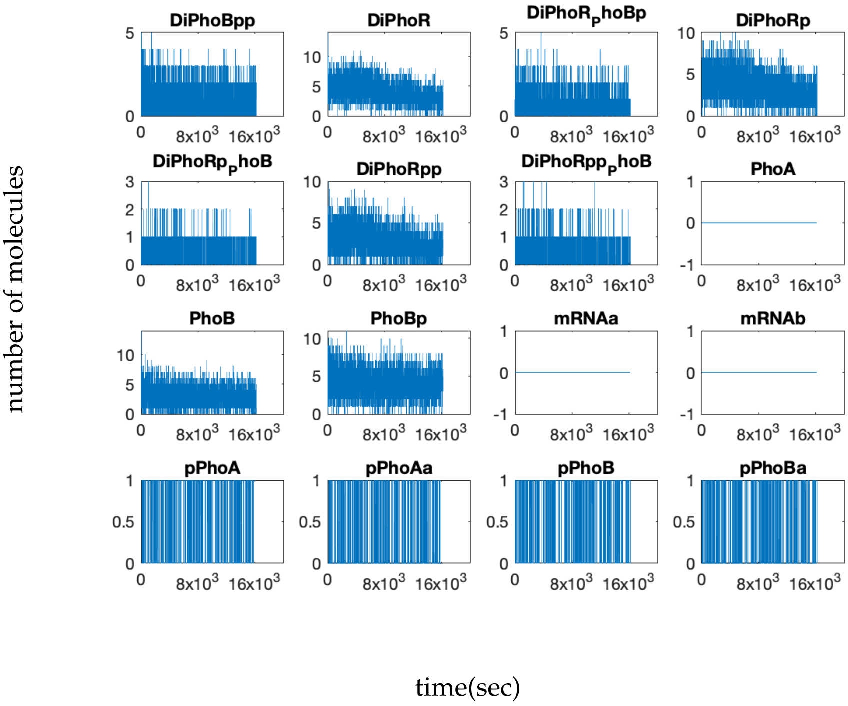

$$fc = 0.3, bf = 10, uf = 0.01$$

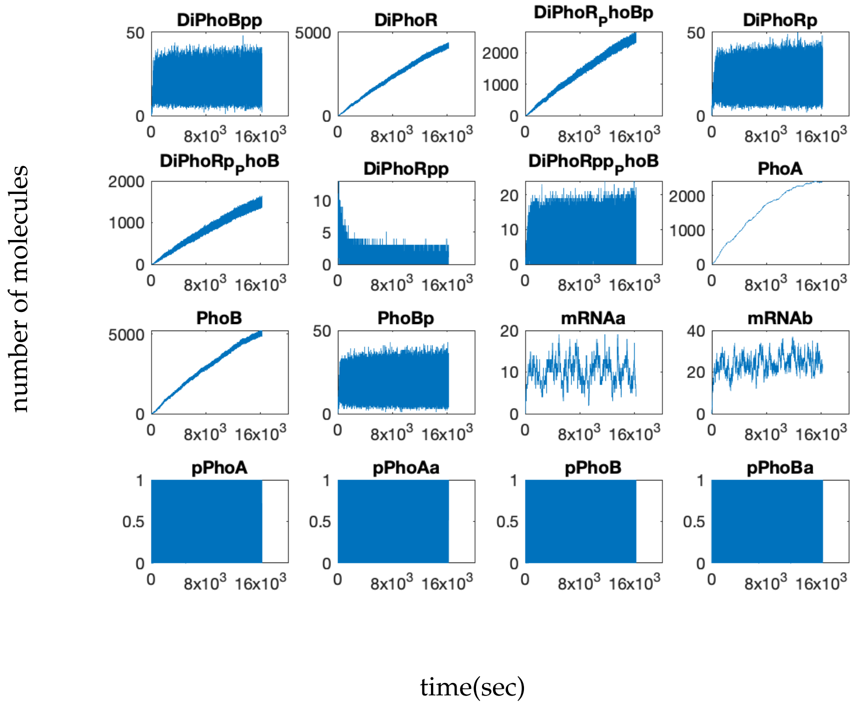

$$\mathrm{fc} = 0.3, \mathrm{bf} = 10, \mathrm{uf} = 0.1$$

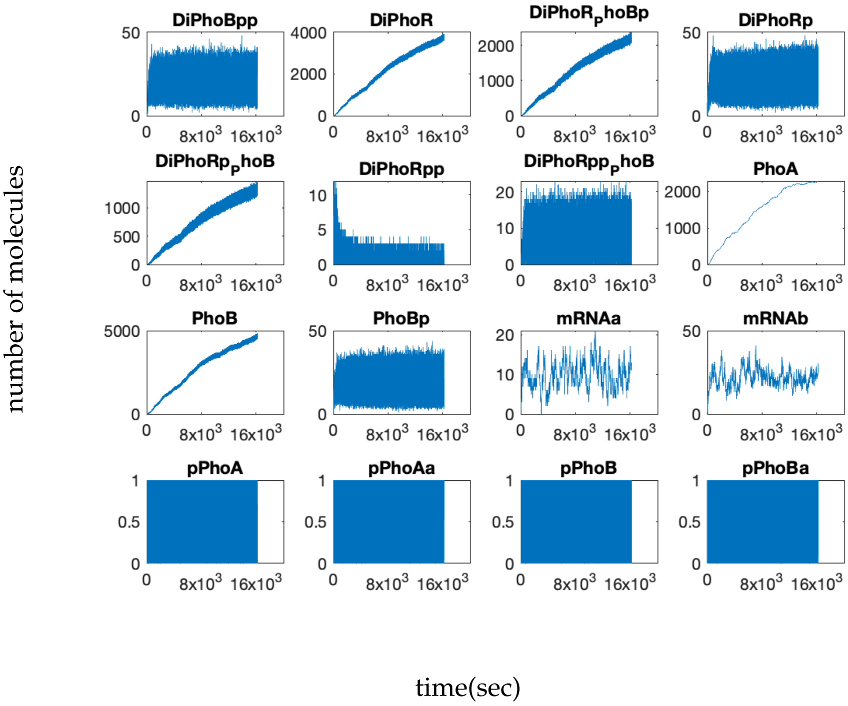

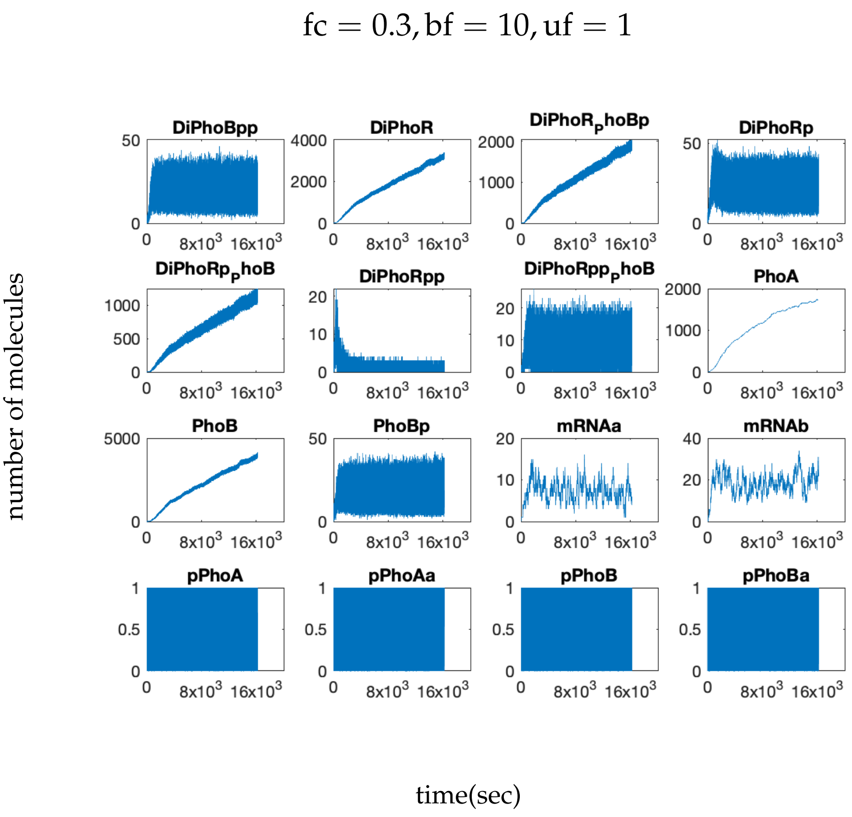

fc = 0.3, bf = 10, uf = 1
DiPhoBpp
DiPhoR
DiPhoR$_p$hoBp
DiPhoRp
DiPhoRp$_p$hoB
DiPhoRpp
DiPhoRpp$_p$hoB
PhoA
PhoB
PhoBp
mRNAa
mRNAb
pPhoA
pPhoAa
pPhoB
pPhoBa
number of molecules
time(sec)
8x10^3
16x10^3

$$\mathrm{fc} = 0.3, \mathrm{bf} = 10, \mathrm{uf} = 10$$

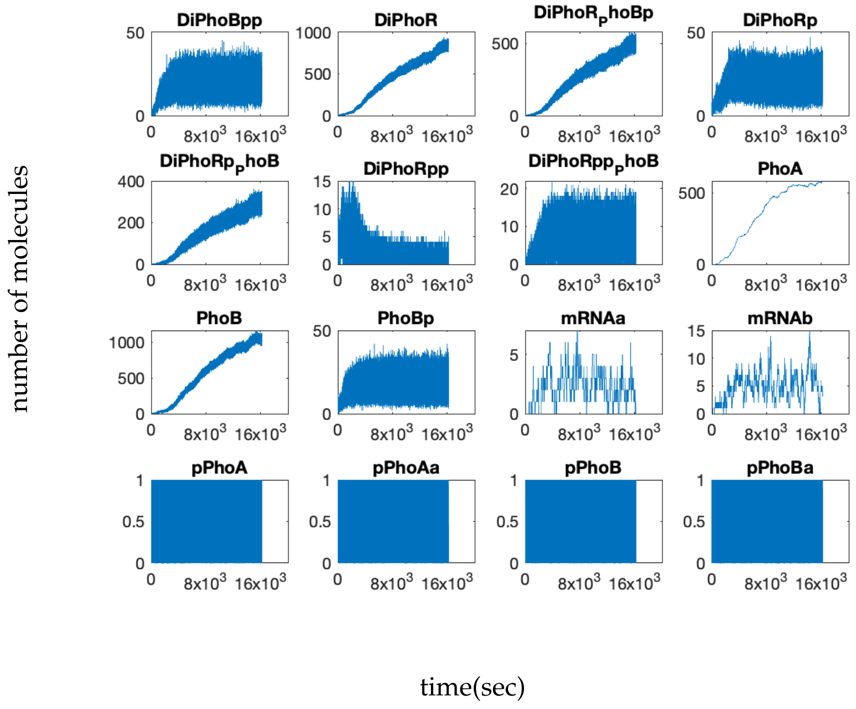

$$fc = 0.3, bf = 10, uf = 100$$

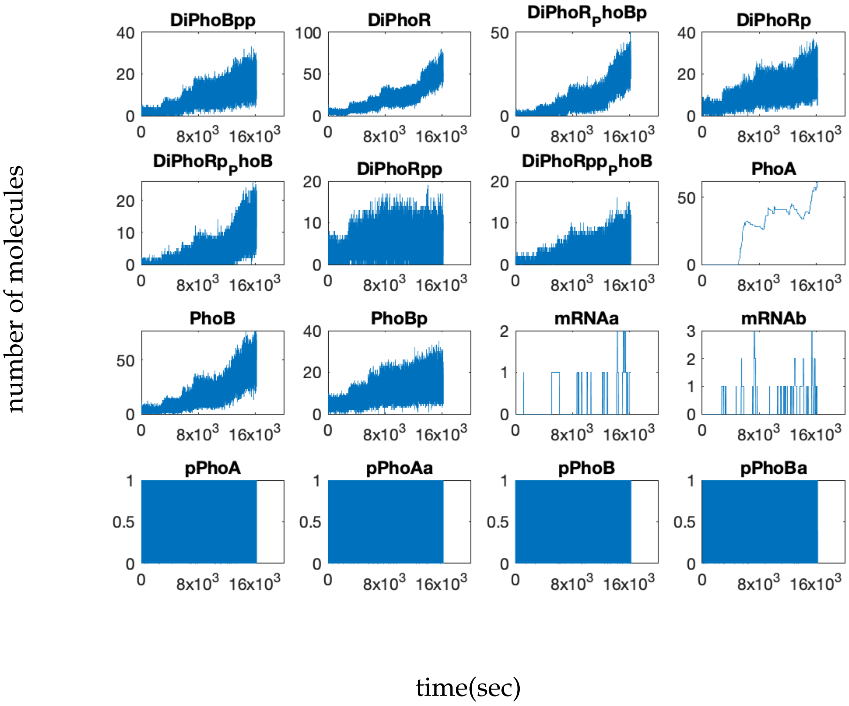

$$\mathrm{fc} = 0.3, \mathrm{bf} = 100, \mathrm{uf} = 0.01$$

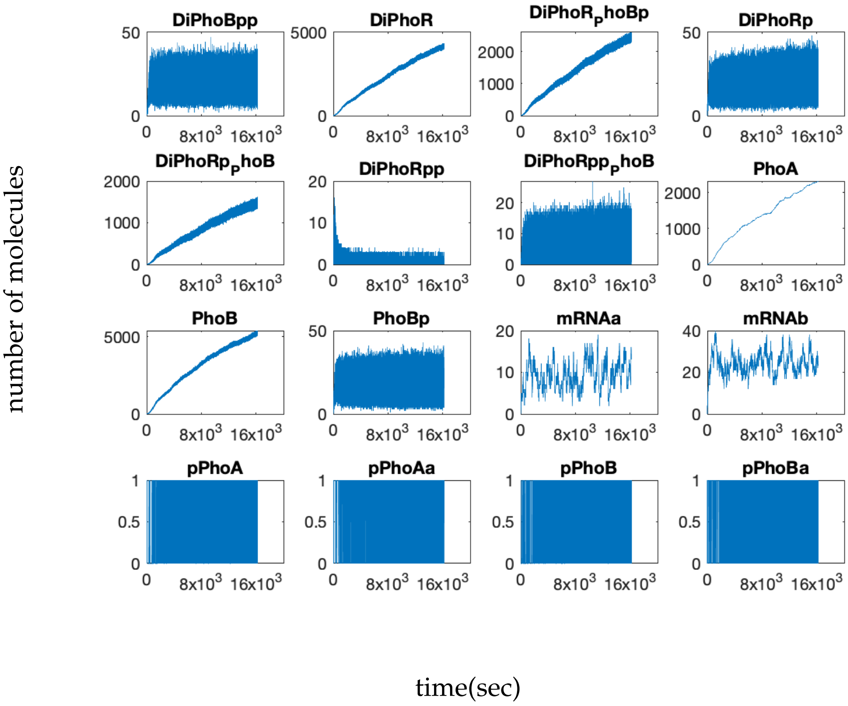

$$\mathrm{fc} = 0.3, \mathrm{bf} = 100, \mathrm{uf} = 0.1$$

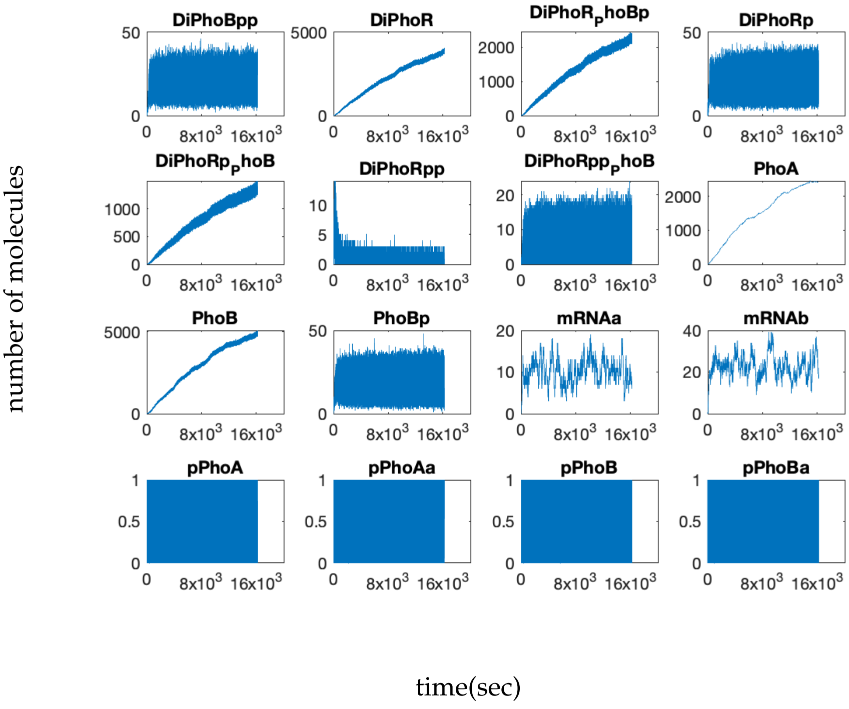

$$\mathrm{fc} = 0.3, \mathrm{bf} = 100, \mathrm{uf} = 1$$

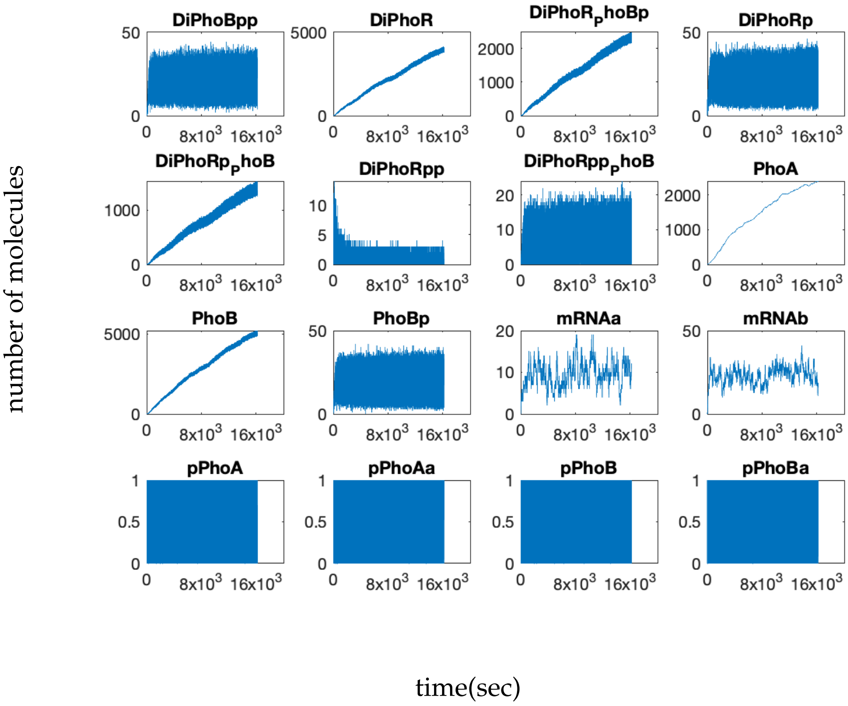

$$\mathrm{fc} = 0.3, \mathrm{bf} = 100, \mathrm{uf} = 10$$

number of molecules

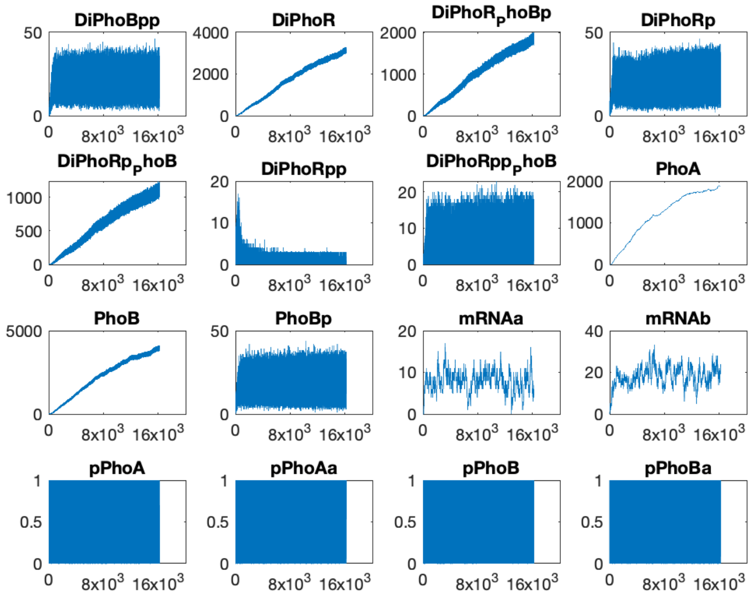

time(sec)

$$\mathrm{fc} = 0.3, \mathrm{bf} = 100, \mathrm{uf} = 100$$

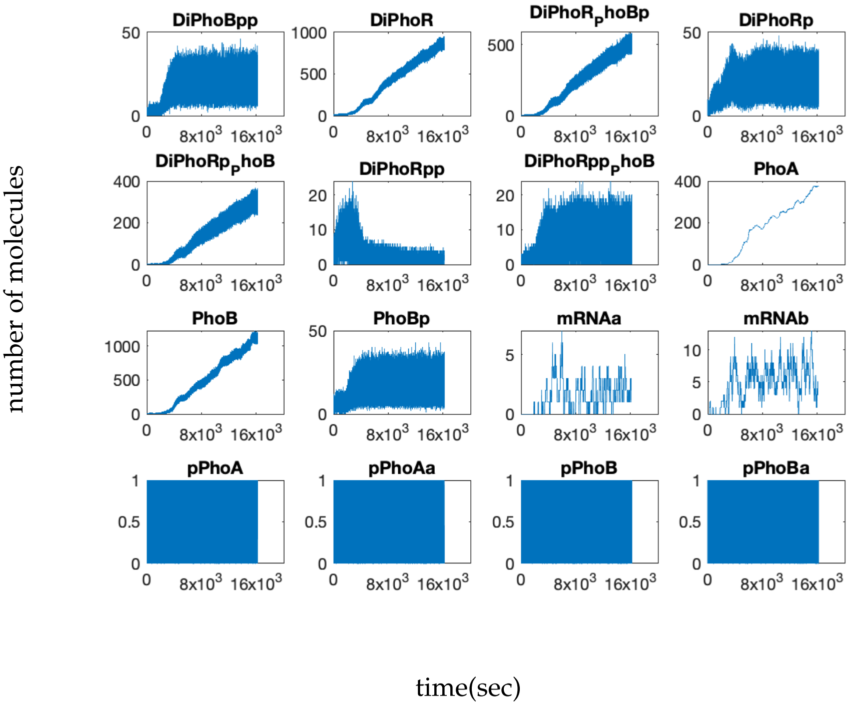

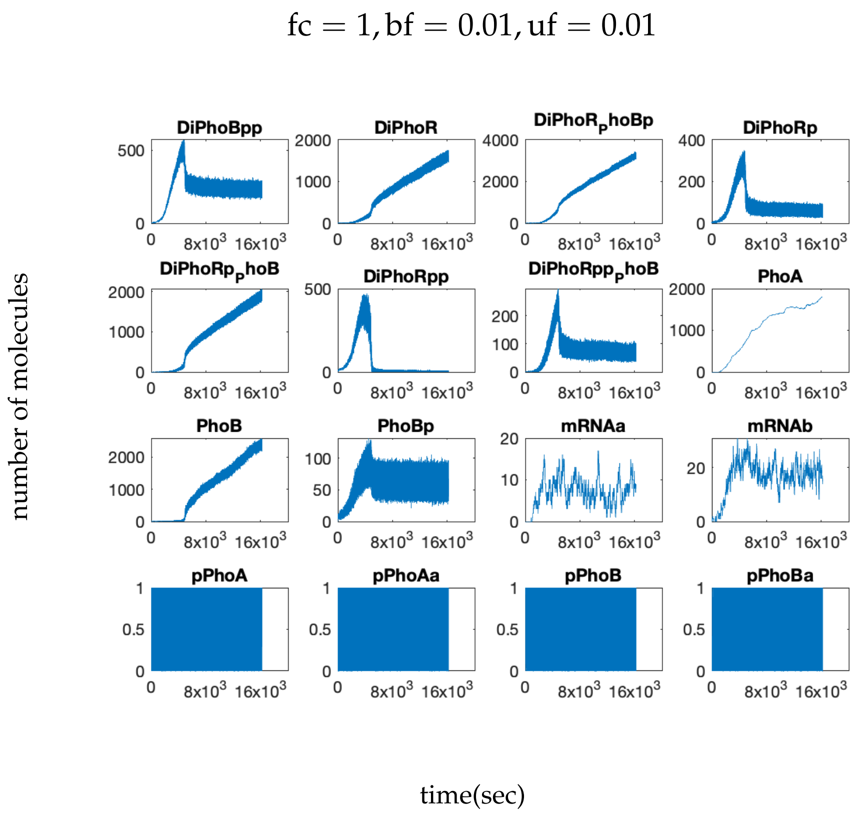
fc = 1, bf = 0.01, uf = 0.01
DiPhoBpp
DiPhoR
DiPhoR_PhoBp
DiPhoRp
DiPhoRp_PhoB
DiPhoRpp
DiPhoRpp_PhoB
PhoA
PhoB
PhoBp
mRNAa
mRNAb
pPhoA
pPhoAa
pPhoB
pPhoBa
number of molecules
time(sec)

$$fc = 1, bf = 0.01, uf = 0.1$$

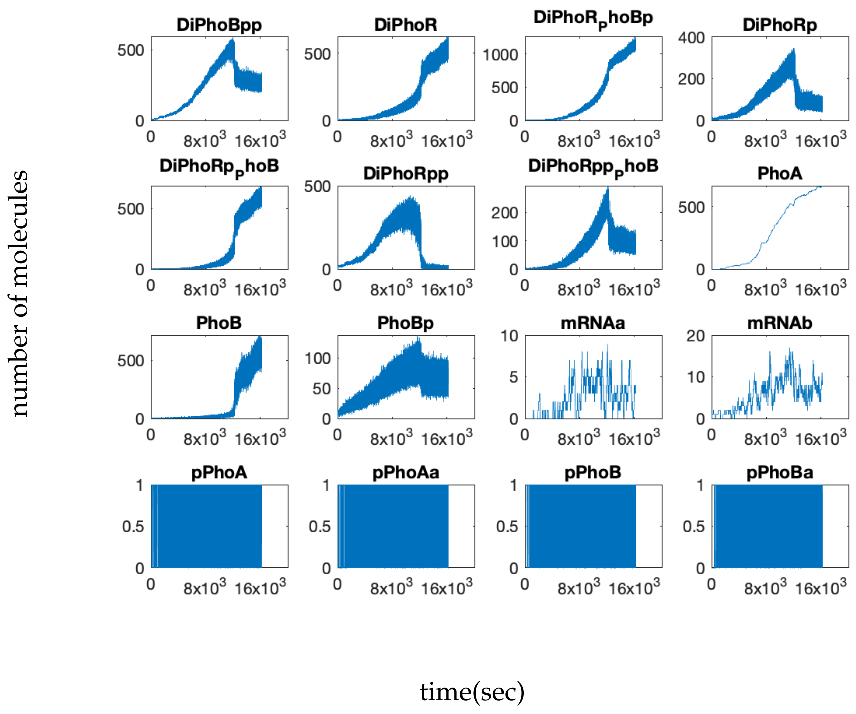

$$fc = 1, bf = 0.01, uf = 1$$

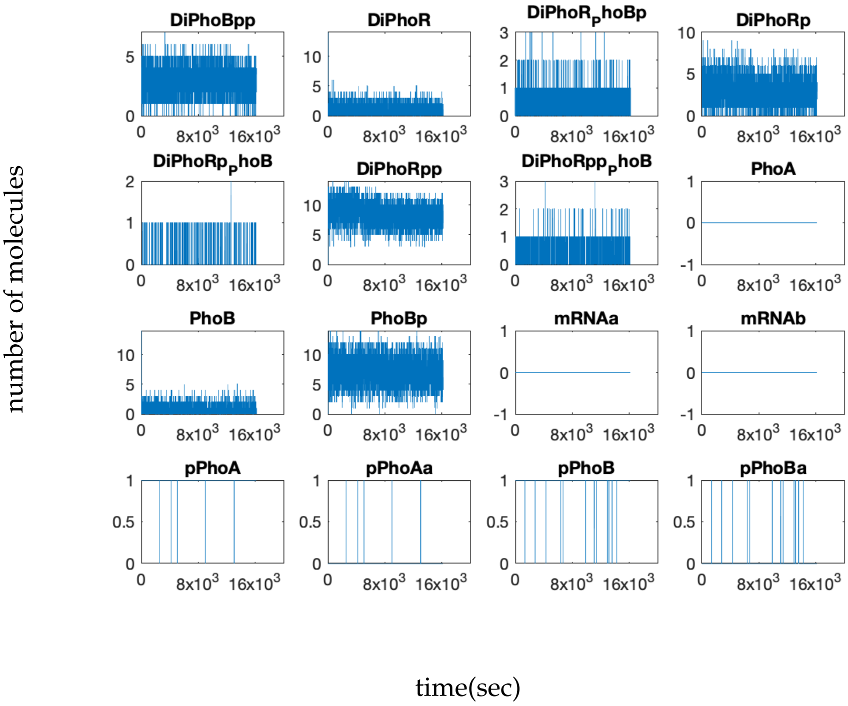

$$\mathrm{fc} = 1, \mathrm{bf} = 0.01, \mathrm{uf} = 10$$

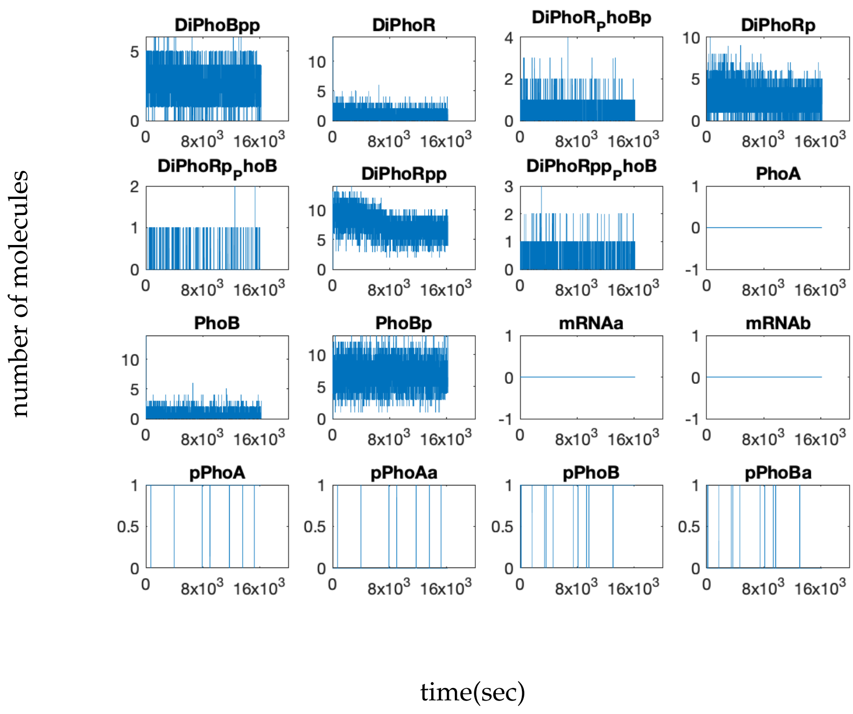

$$\mathrm{fc} = 1, \mathrm{bf} = 0.01, \mathrm{uf} = 100$$

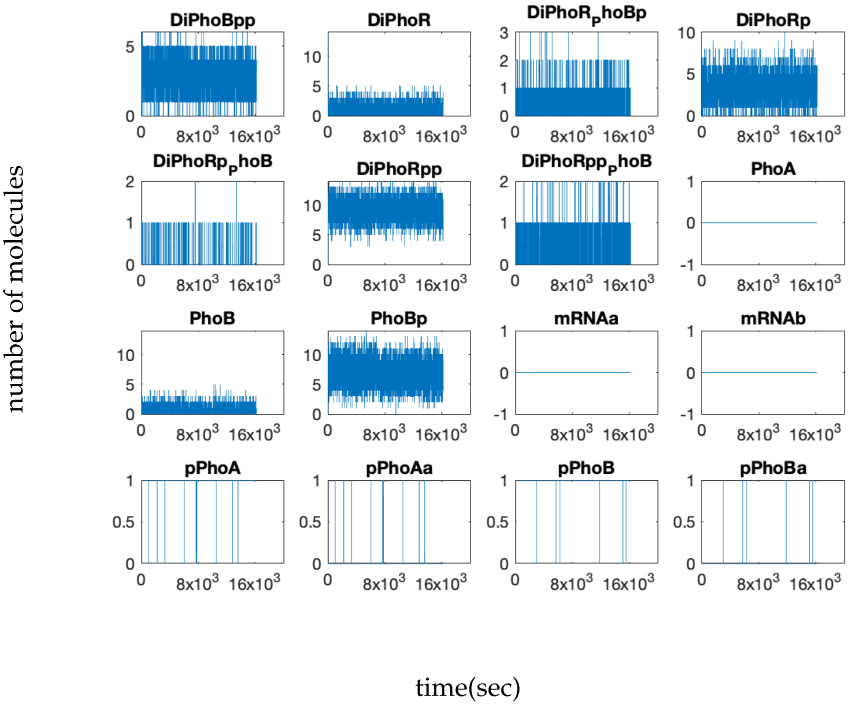

$$\mathrm{fc} = 1, \mathrm{bf} = 0.1, \mathrm{uf} = 0.01$$

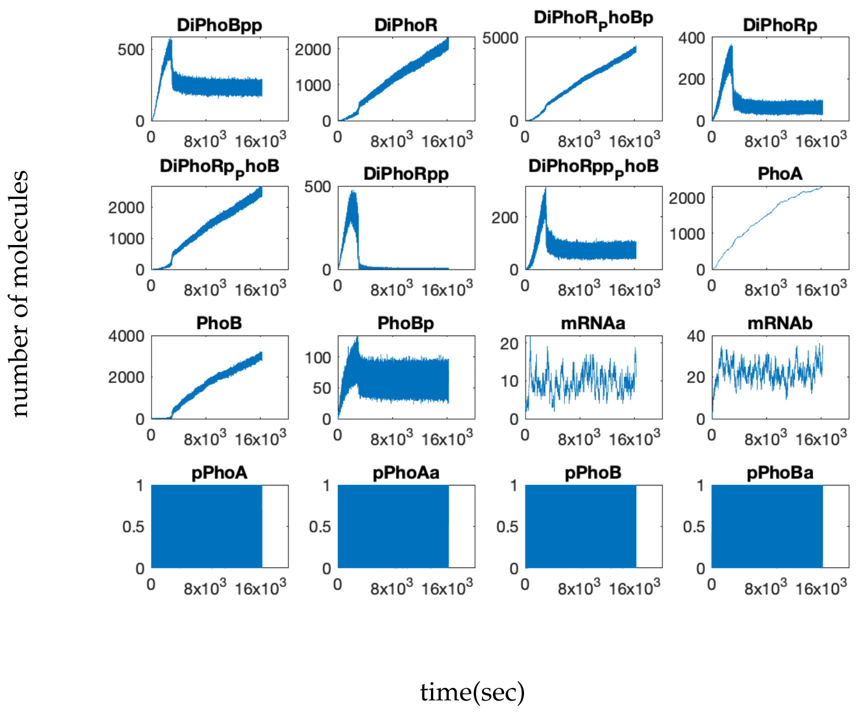

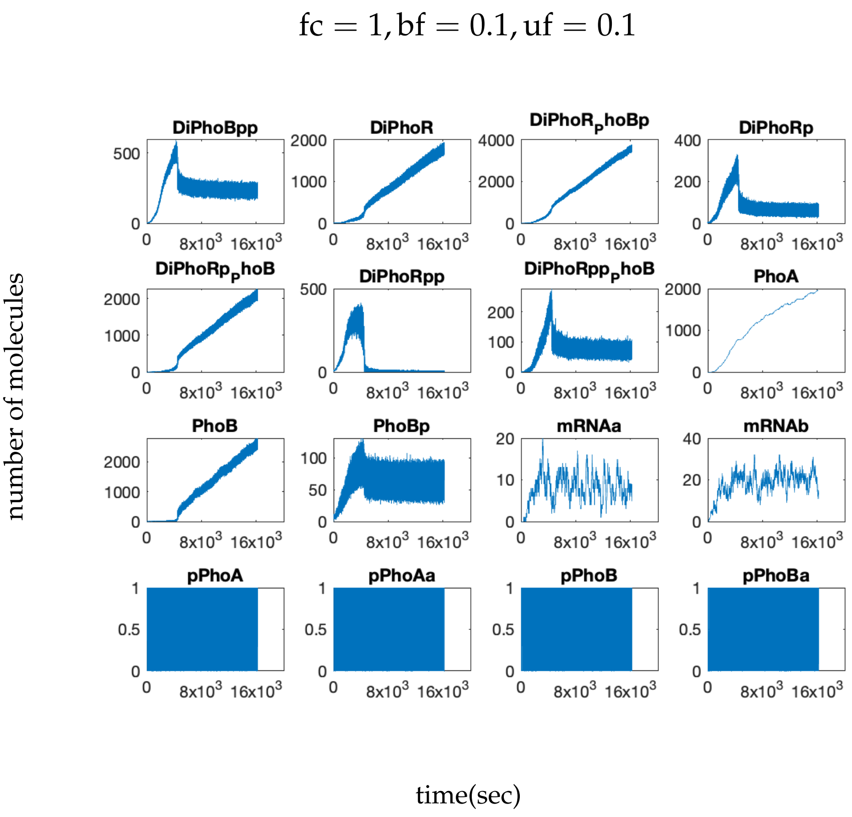
fc = 1, bf = 0.1, uf = 0.1
number of molecules
DiPhoBpp
DiPhoR
DiPhoR_PhoBp
DiPhoRp
DiPhoRp_PhoB
DiPhoRpp
DiPhoRpp_PhoB
PhoA
PhoB
PhoBp
mRNAa
mRNAb
pPhoA
pPhoAa
pPhoB
pPhoBa
0
8x10^3
16x10^3
time(sec)

$$fc = 1, bf = 0.1, uf = 1$$

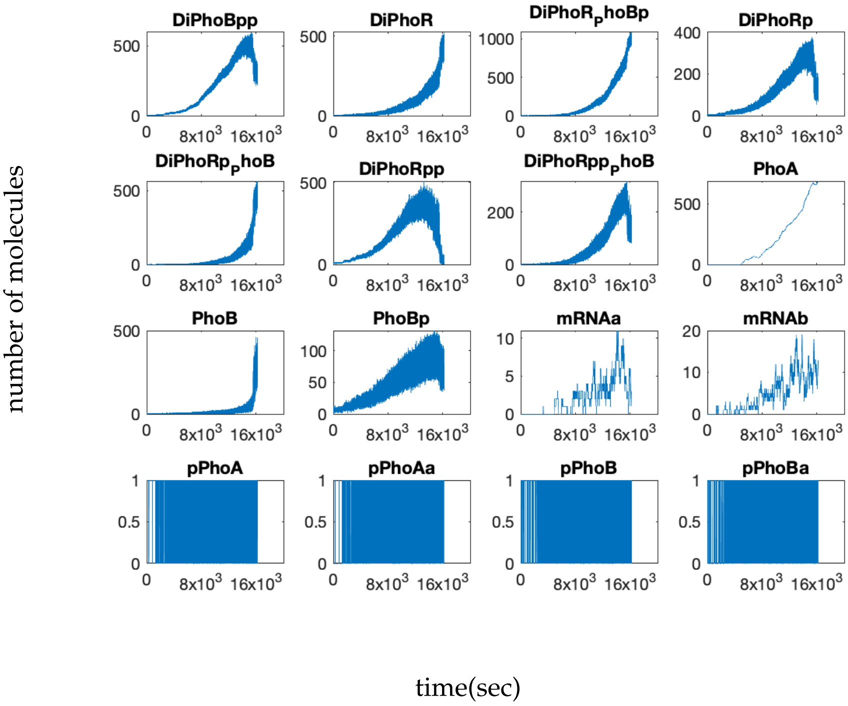
DiPhoBpp
DiPhoR
DiPhoR$_P$hoBp
DiPhoRp
DiPhoRp$_P$hoB
DiPhoRpp
DiPhoRpp$_P$hoB
PhoA
PhoB
PhoBp
mRNAa
mRNAb
pPhoA
pPhoAa
pPhoB
pPhoBa
0
8x10^3
16x10^3
number of molecules
time(sec)

$$\mathrm{fc} = 1, \mathrm{bf} = 0.1, \mathrm{uf} = 10$$

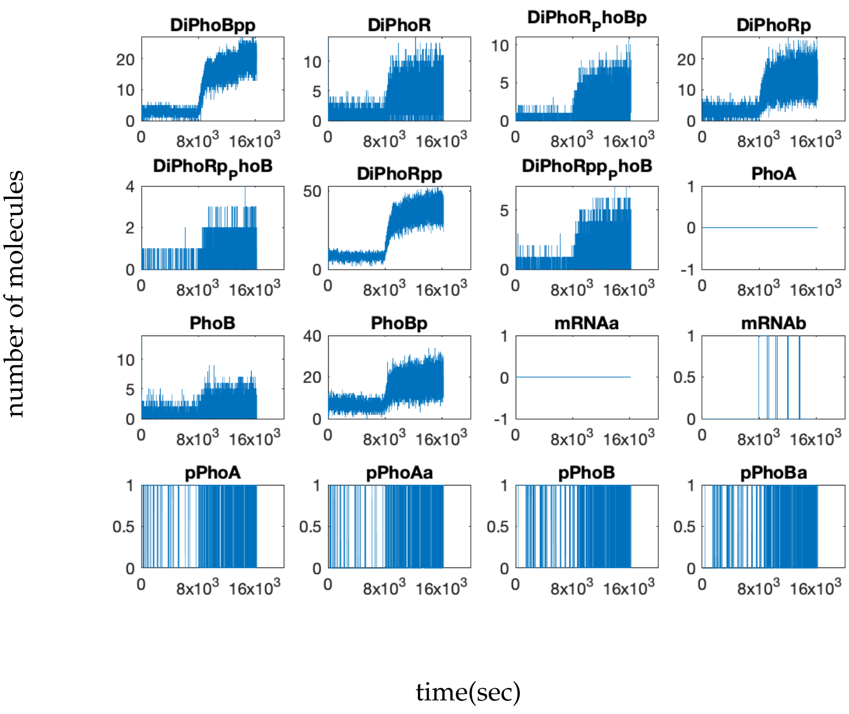

$$fc = 1, bf = 0.1, uf = 100$$

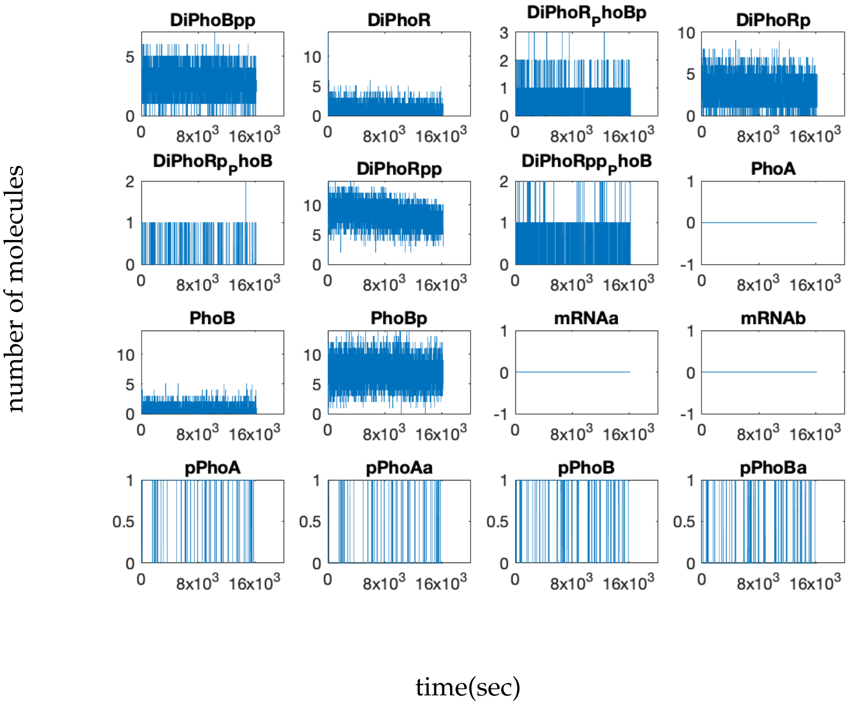

$fc = 1, bf = 1, uf = 0.01$

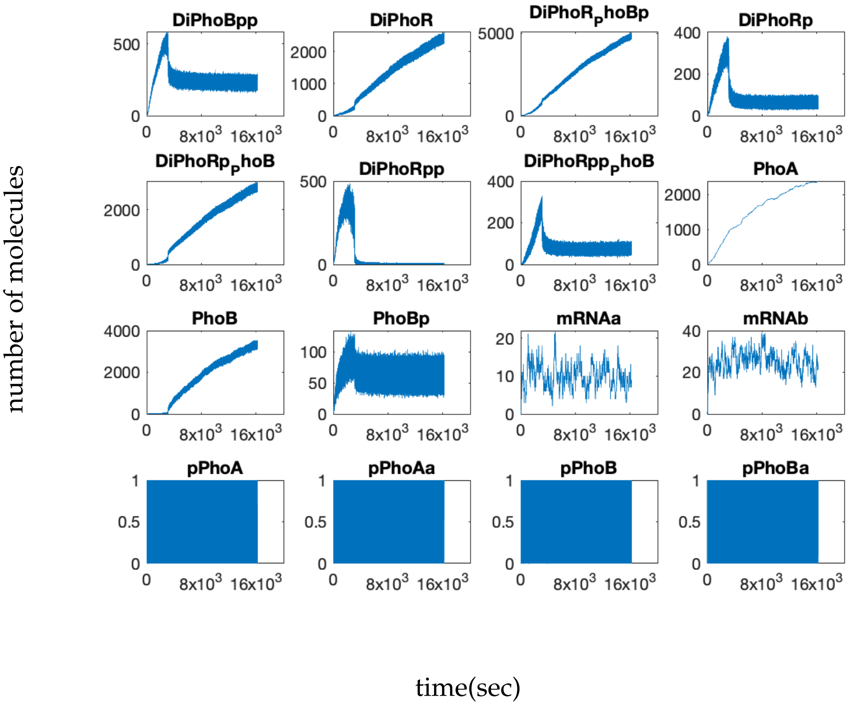

$$\mathrm{fc} = 1, \mathrm{bf} = 1, \mathrm{uf} = 0.1$$

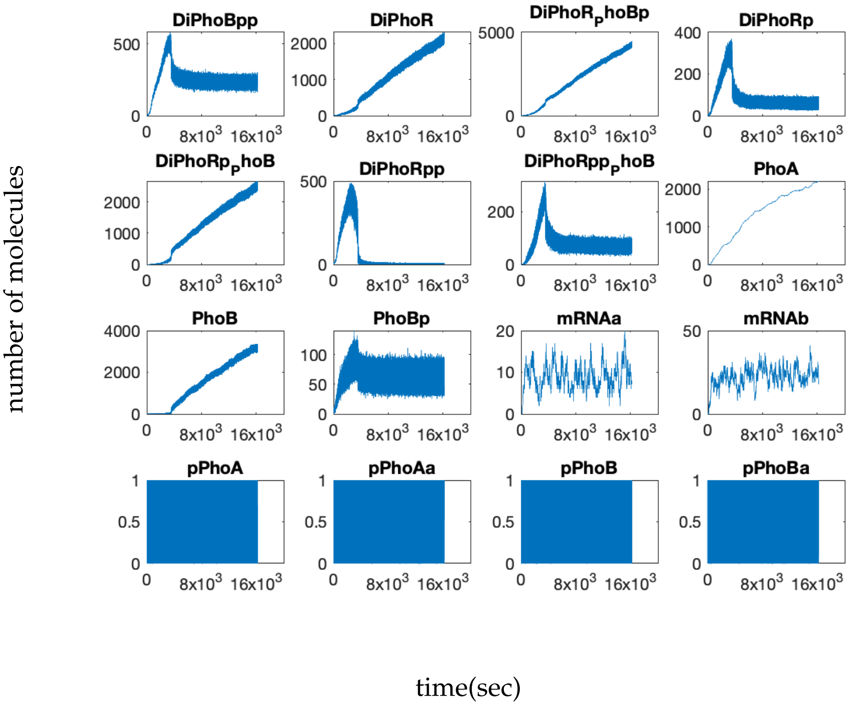

$$fc = 1, bf = 1, uf = 1$$

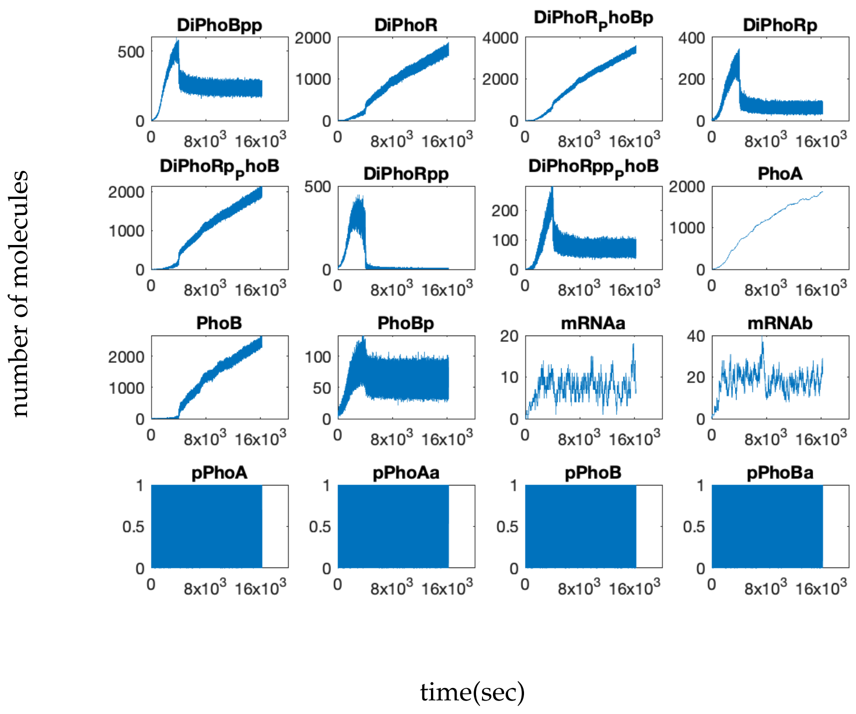
DiPhoBpp
DiPhoR
DiPhoR_PhoBp
DiPhoRp
DiPhoRp_PhoB
DiPhoRpp
DiPhoRpp_PhoB
PhoA
PhoB
PhoBp
mRNAa
mRNAb
pPhoA
pPhoAa
pPhoB
pPhoBa
number of molecules
time(sec)

$\mathrm{fc} = 1, \mathrm{bf} = 1, \mathrm{uf} = 10$

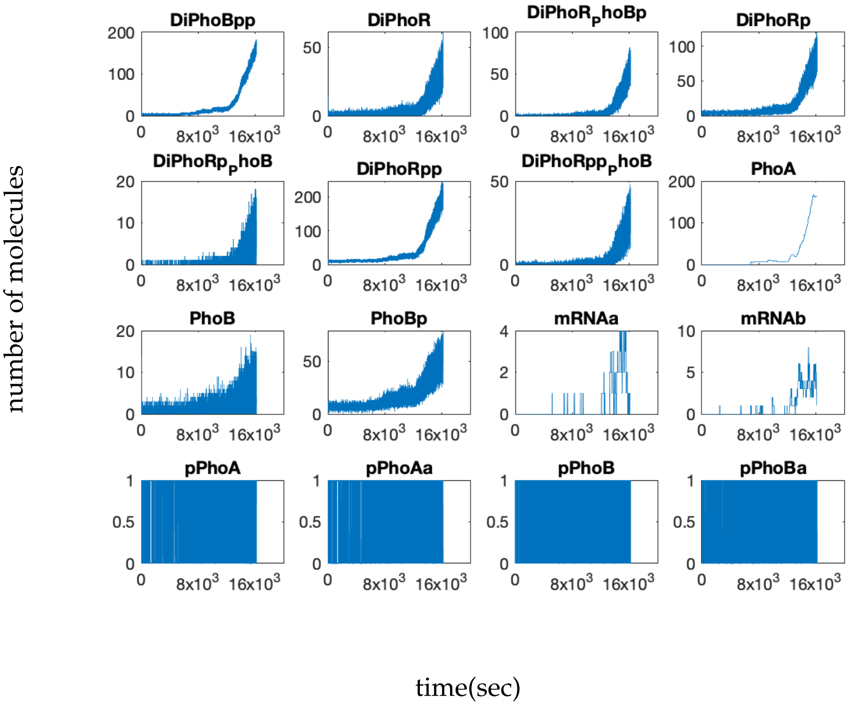

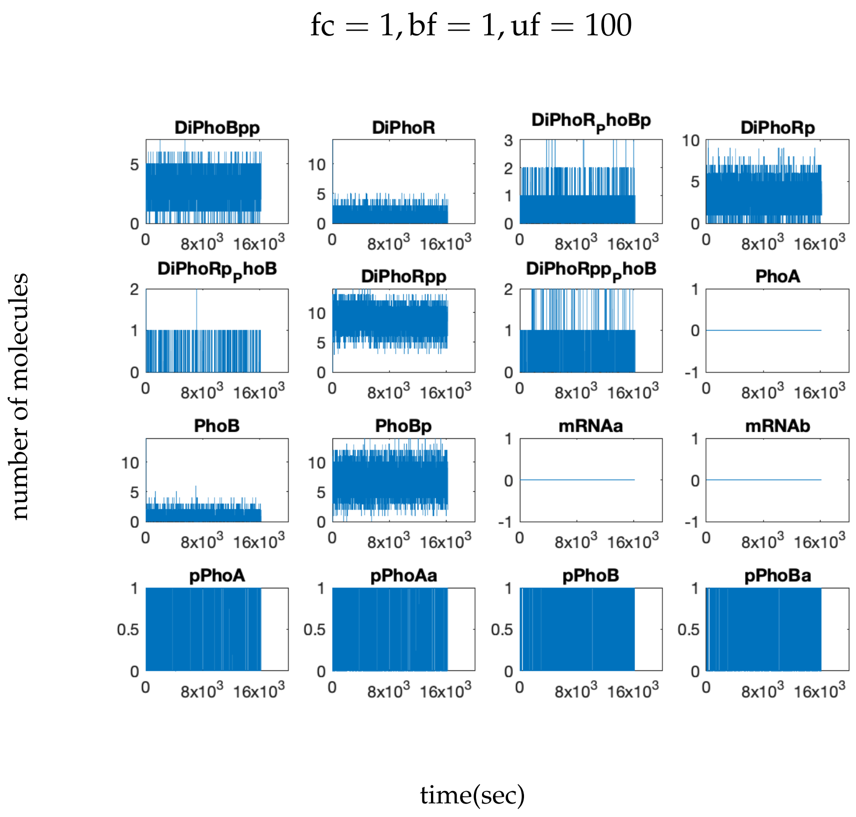

fc = 1, bf = 1, uf = 100
number of molecules
DiPhoBpp
DiPhoR
DiPhoR$_P$hoBp
DiPhoRp
DiPhoRp$_P$hoB
DiPhoRpp
DiPhoRpp$_P$hoB
PhoA
PhoB
PhoBp
mRNAa
mRNAb
pPhoA
pPhoAa
pPhoB
pPhoBa
8x10^3
16x10^3
time(sec)

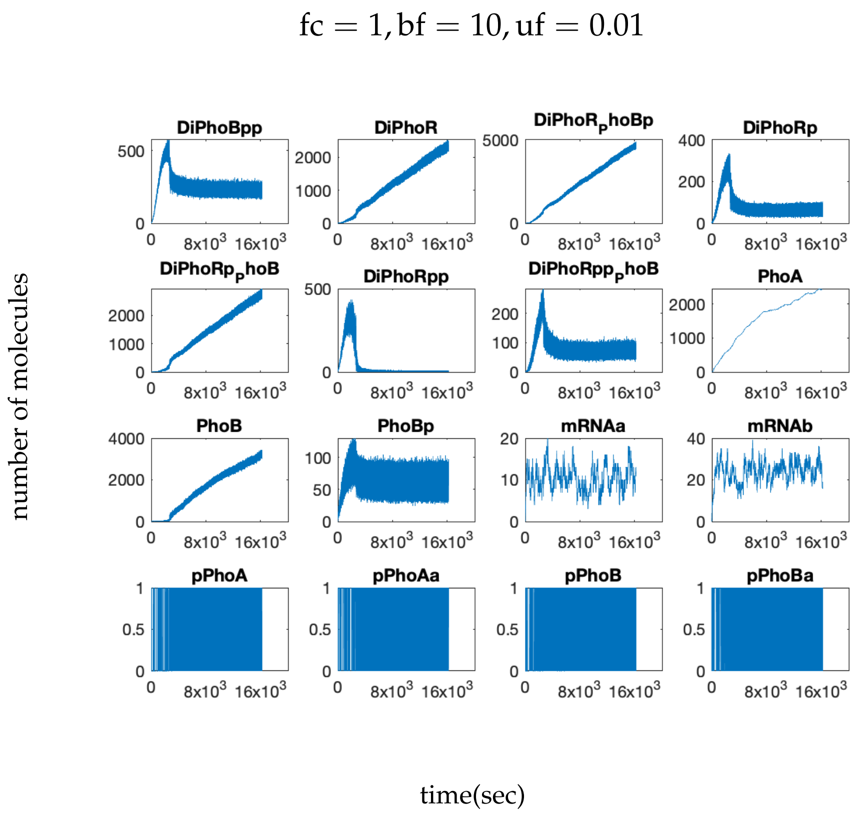
fc = 1, bf = 10, uf = 0.01
DiPhoBpp
DiPhoR
DiPhoR_PhoBp
DiPhoRp
DiPhoRp_PhoB
DiPhoRpp
DiPhoRpp_PhoB
PhoA
PhoB
PhoBp
mRNAa
mRNAb
pPhoA
pPhoAa
pPhoB
pPhoBa
number of molecules
time(sec)

$$fc = 1, bf = 10, uf = 0.1$$

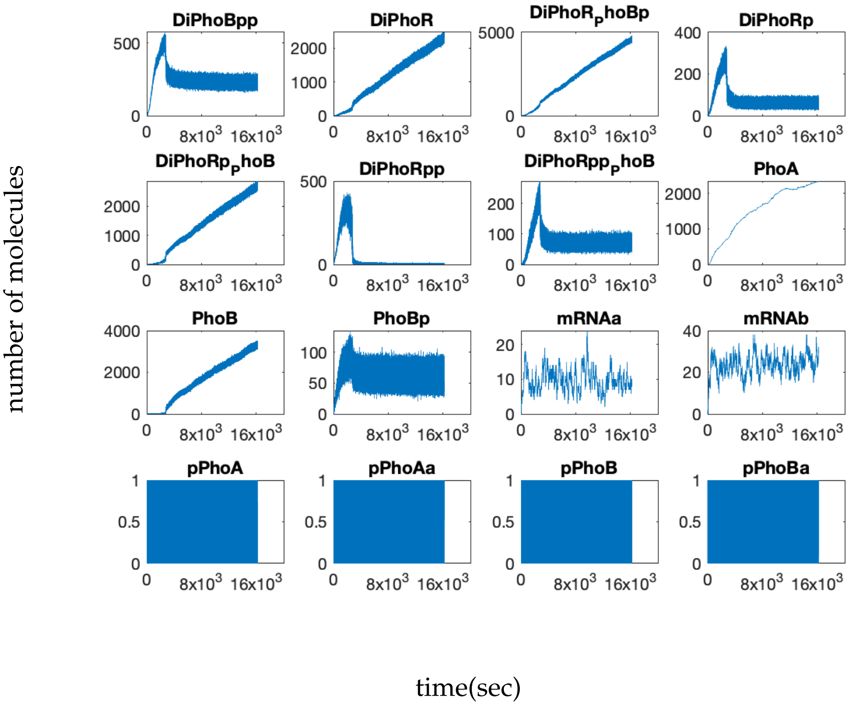

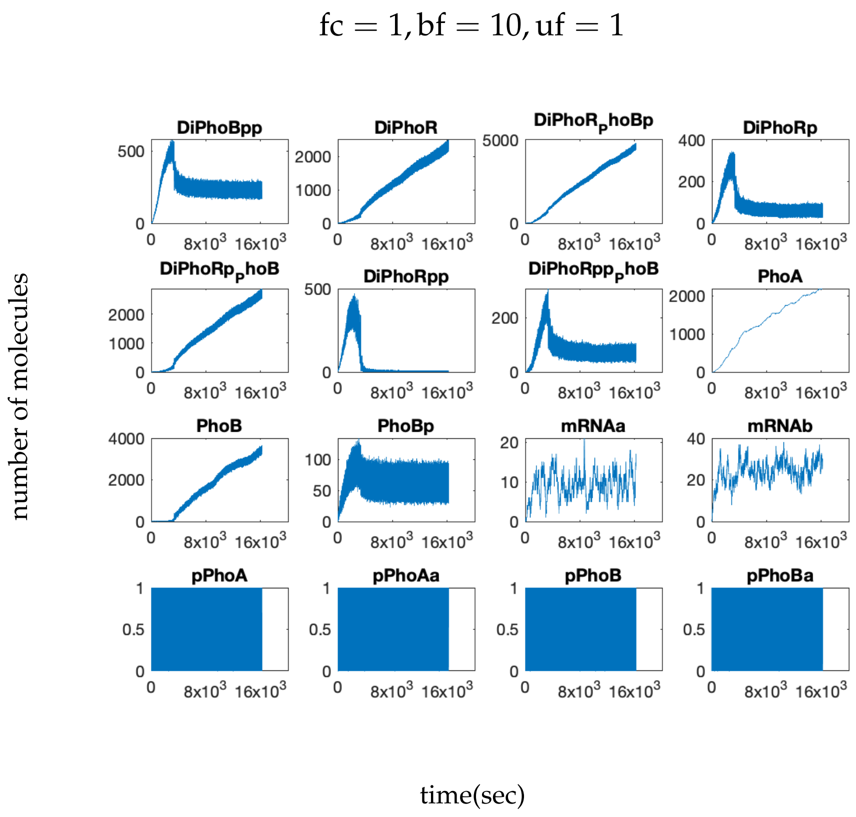
fc = 1, bf = 10, uf = 1
DiPhoBpp
DiPhoR
DiPhoR$_P$hoBp
DiPhoRp
DiPhoRp$_P$hoB
DiPhoRpp
DiPhoRpp$_P$hoB
PhoA
PhoB
PhoBp
mRNAa
mRNAb
pPhoA
pPhoAa
pPhoB
pPhoBa
number of molecules
time(sec)

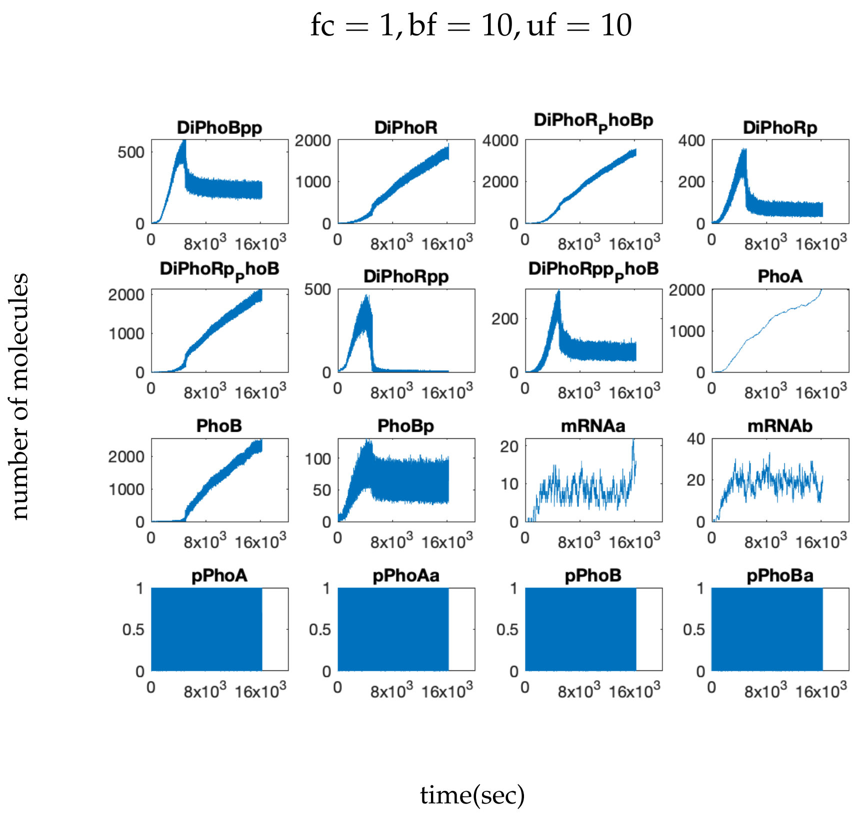
fc = 1, bf = 10, uf = 10
DiPhoBpp
DiPhoR
DiPhoR_PhoBp
DiPhoRp
DiPhoRp_PhoB
DiPhoRpp
DiPhoRpp_PhoB
PhoA
PhoB
PhoBp
mRNAa
mRNAb
pPhoA
pPhoAa
pPhoB
pPhoBa
number of molecules
time(sec)

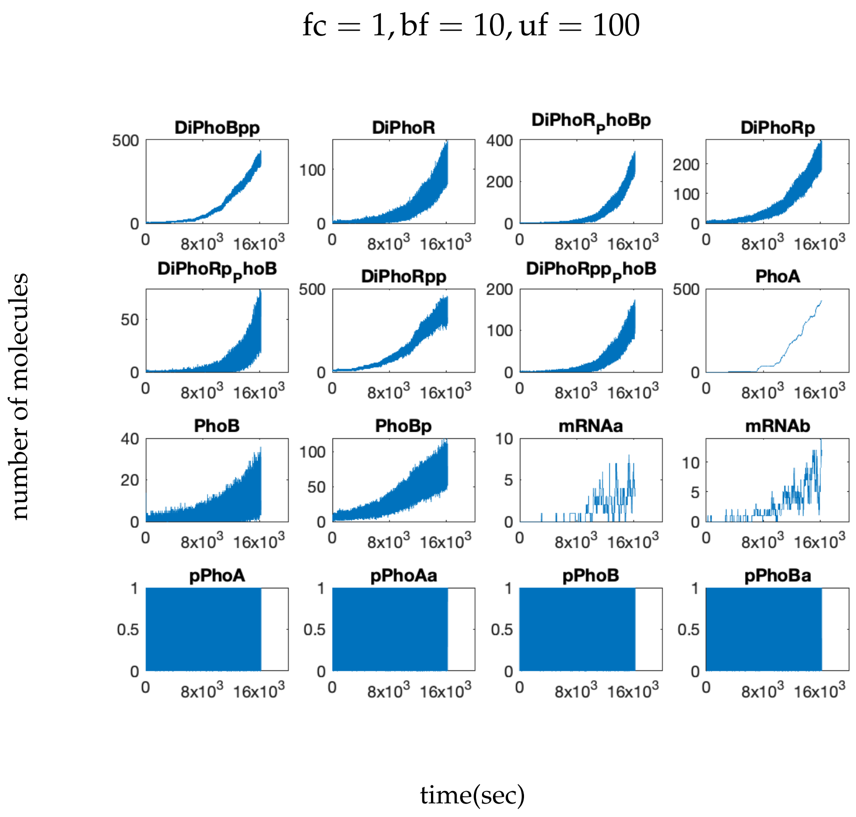
fc = 1, bf = 10, uf = 100
DiPhoBpp
DiPhoR
DiPhoR_PhoBp
DiPhoRp
DiPhoRp_PhoB
DiPhoRpp
DiPhoRpp_PhoB
PhoA
PhoB
PhoBp
mRNAa
mRNAb
pPhoA
pPhoAa
pPhoB
pPhoBa
number of molecules
time(sec)

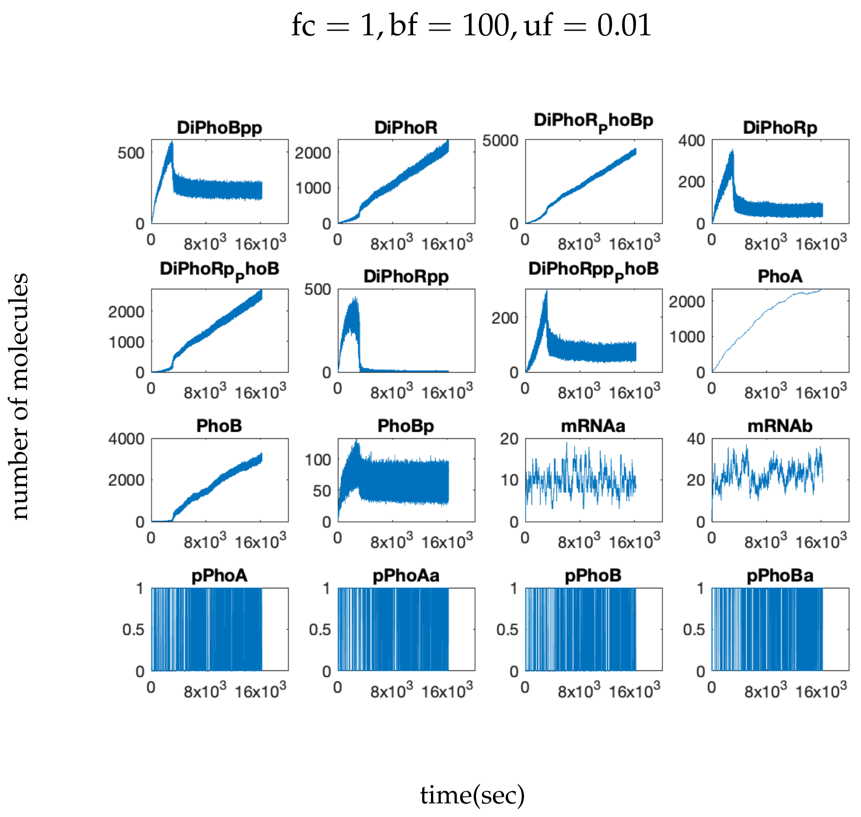
fc = 1, bf = 100, uf = 0.01
number of molecules
time(sec)
DiPhoBpp
DiPhoR
DiPhoRPhoBp
DiPhoRp
DiPhoRpPhoB
DiPhoRpp
DiPhoRppPhoB
PhoA
PhoB
PhoBp
mRNAa
mRNAb
pPhoA
pPhoAa
pPhoB
pPhoBa
0
8x10^3
16x10^3

$$fc = 1, bf = 100, uf = 0.1$$

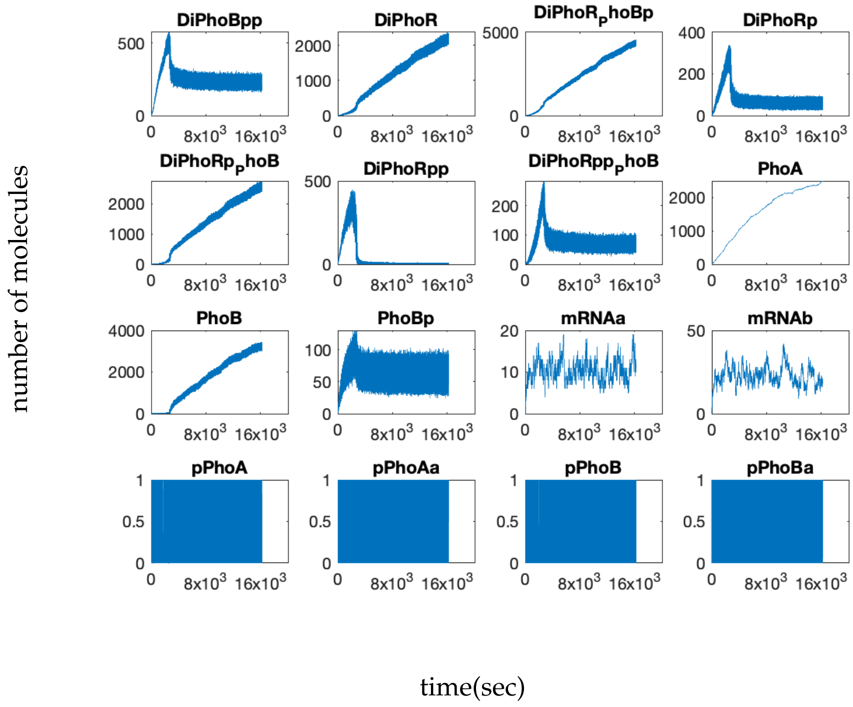

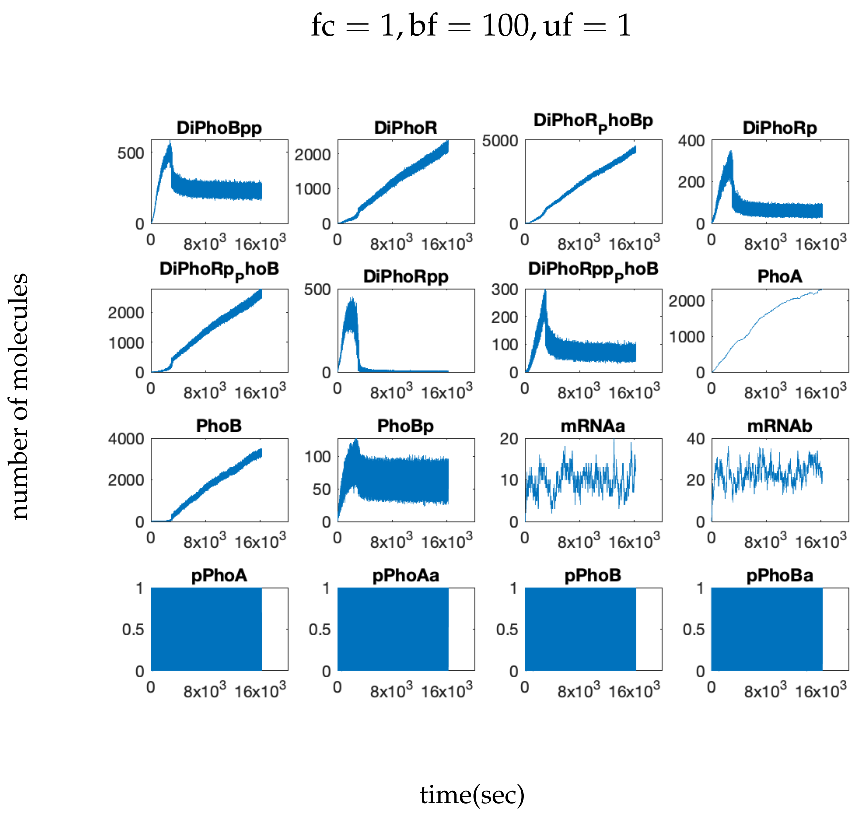
fc = 1, bf = 100, uf = 1
DiPhoBpp
DiPhoR
DiPhoR_PhoBp
DiPhoRp
DiPhoRp_PhoB
DiPhoRpp
DiPhoRpp_PhoB
PhoA
PhoB
PhoBp
mRNAa
mRNAb
pPhoA
pPhoAa
pPhoB
pPhoBa
number of molecules
time(sec)

$$\mathrm{fc} = 1, \mathrm{bf} = 100, \mathrm{uf} = 10$$

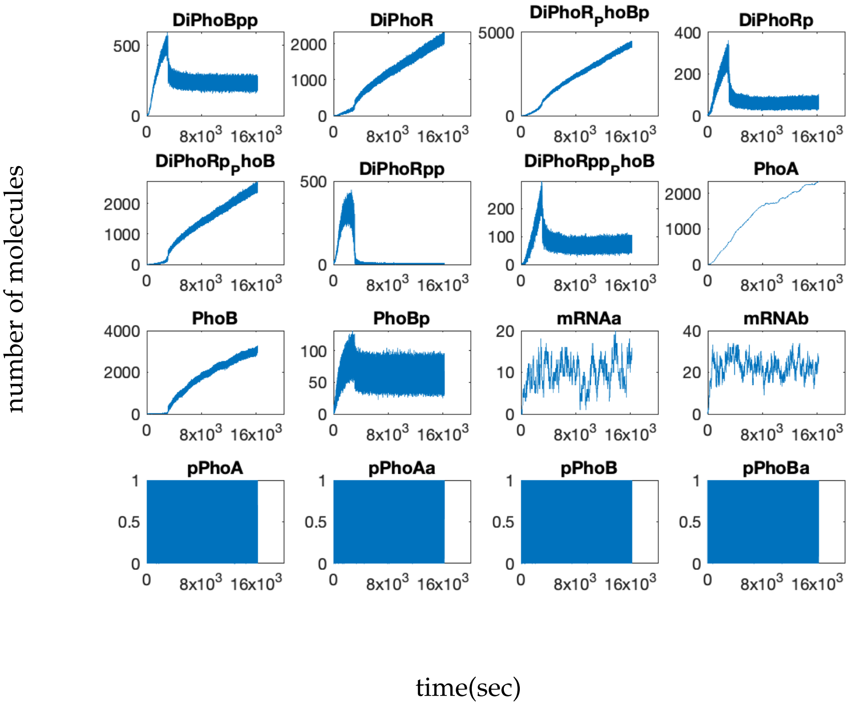

$$\mathrm{fc} = 1, \mathrm{bf} = 100, \mathrm{uf} = 100$$

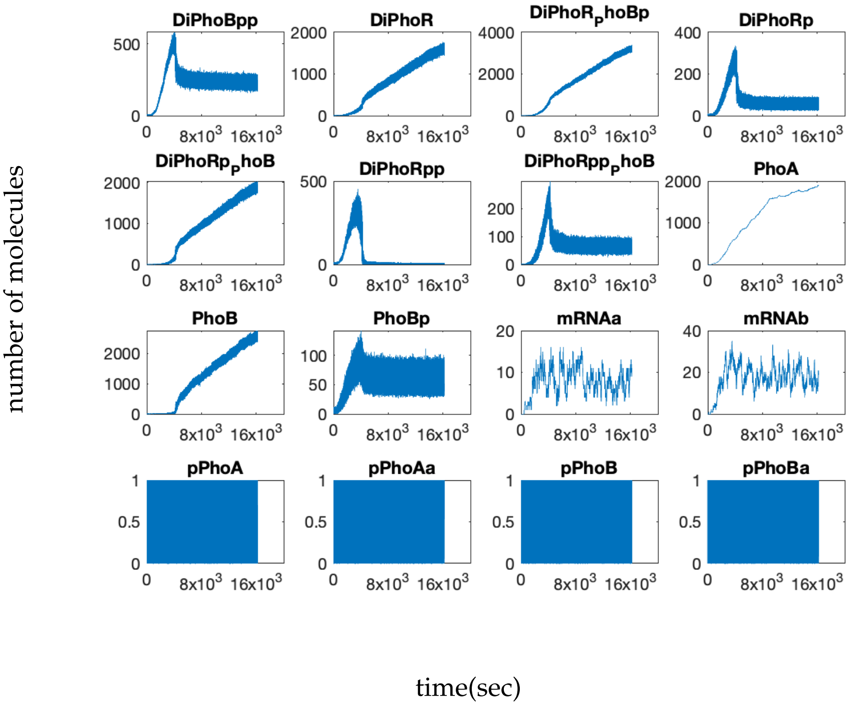

References

1. Gillespie, D.T. Exact stochastic simulation of coupled chemical reactions. *The journal of physical chemistry* **1977**, *81*, 2340–2361.
2. Uluşeker, C.; Torres-Bacete, J.; García, J.L.; Hanczyc, M.M.; Nogales, J.; Kahramanoğulları, O. Quantifying dynamic mechanisms of auto-regulation in *Escherichia coli* with synthetic promoter in response to varying external phosphate levels. *Scientific reports* **2019**, *9*, 2076.
3. Ingalls, B.P. *Mathematical modeling in systems biology: an introduction*; MIT press, 2013.
4. Gardner, S.G.; McCleary, W.R. Control of the phoBR Regulon in *Escherichia coli*. *EcoSal Plus* **2019**, *6*.
